# Supplementary material for: Synthesis of Isomeric Phosphoubiquitin Chains Reveals that Phosphorylation Controls Deubiquitinase Activity and Specificity
Source: Cell Rep. 2016 Jul 14;16(4):1180–93. doi: 10.1016/j.celrep.2016.06.064 (PMC4967478; doi:10.1016/j.celrep.2016.06.064)
Supplement: Document S1. Supplemental Experimental Procedures, Figures S1–S5, and Tables S1 and S3 [file mmc1.pdf]

Cell Reports, Volume *16*

Supplemental Information

Synthesis of Isomeric Phosphoubiquitin Chains Reveals that Phosphorylation Controls Deubiquitinase Activity and Specificity

Nicolas Huguenin-Dezot, Virginia De Cesare, Julien Peltier, Axel Knebel, Yosua Adi Kristaryianto, Daniel T. Rogerson, Yogesh Kulathu, Matthias Trost, and Jason W. Chin

1. Supplemental Figures and Legends

Supplemental Figure 1

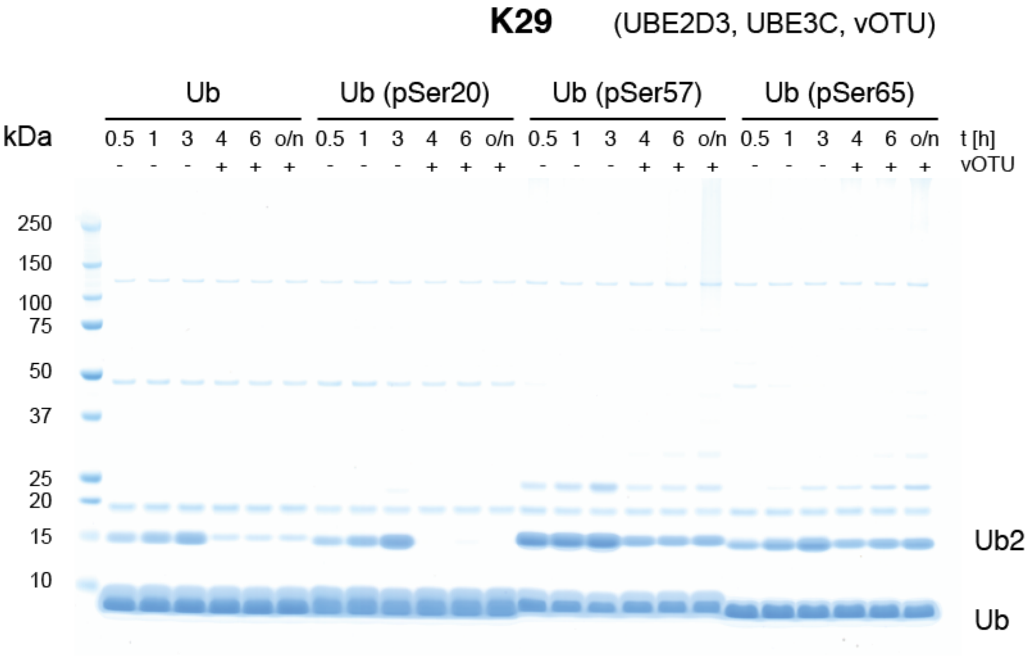

Figure S1, related to Figure 4. K29 chain assembly with phosphoubiquitins.
Full gel from **Figure 4**. K29 chains assembled with Ube1, UBE2D3, UBE3C and followed by coomassie staining. The DUB vOTU was added after the 3h time point. No more Ub dimer is detectable after addition of vOTU for Ub (20pSer).

Supplemental Figure 2

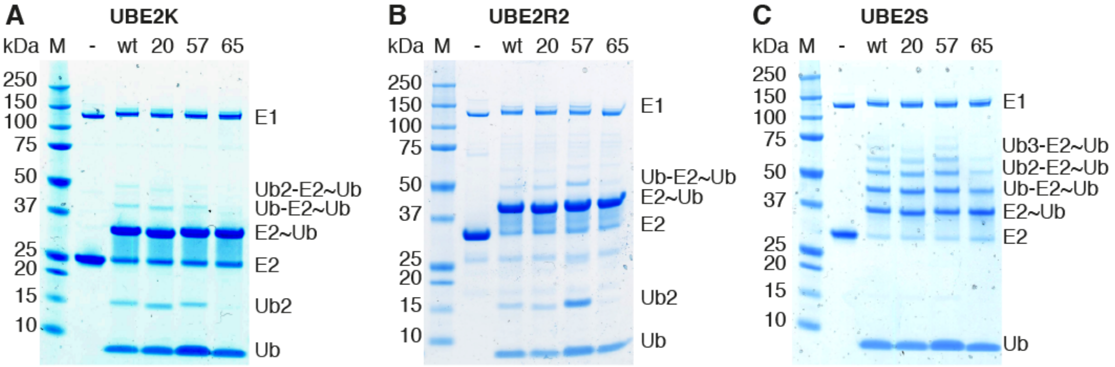

Figure S2, related to Figure 2. E2 charging with phosphoubiquitins.
Coommassie staining of reaction E2-charging reactions containing an E1 (Ube1), ATP, UBE2K **(A)** or UBE2R2 **(B)** or UBE2S **(C)** and either Ub, Ub (pSer20), Ub57(pSer) or Ub65(pSer) after 1h incubation at 30˚C. Ub: Ubiquitin. E2~Ub: Thioester-linked E2-ubiquitin pair. Ub-E2~Ub: Thioester-linked E2-ubiquitin pair with one covalently E2 attached ubiquitin. 20, 57 and 65 respectively designate the phosphorylated serine residue on ubiquitin.

Supplemental Figure 3

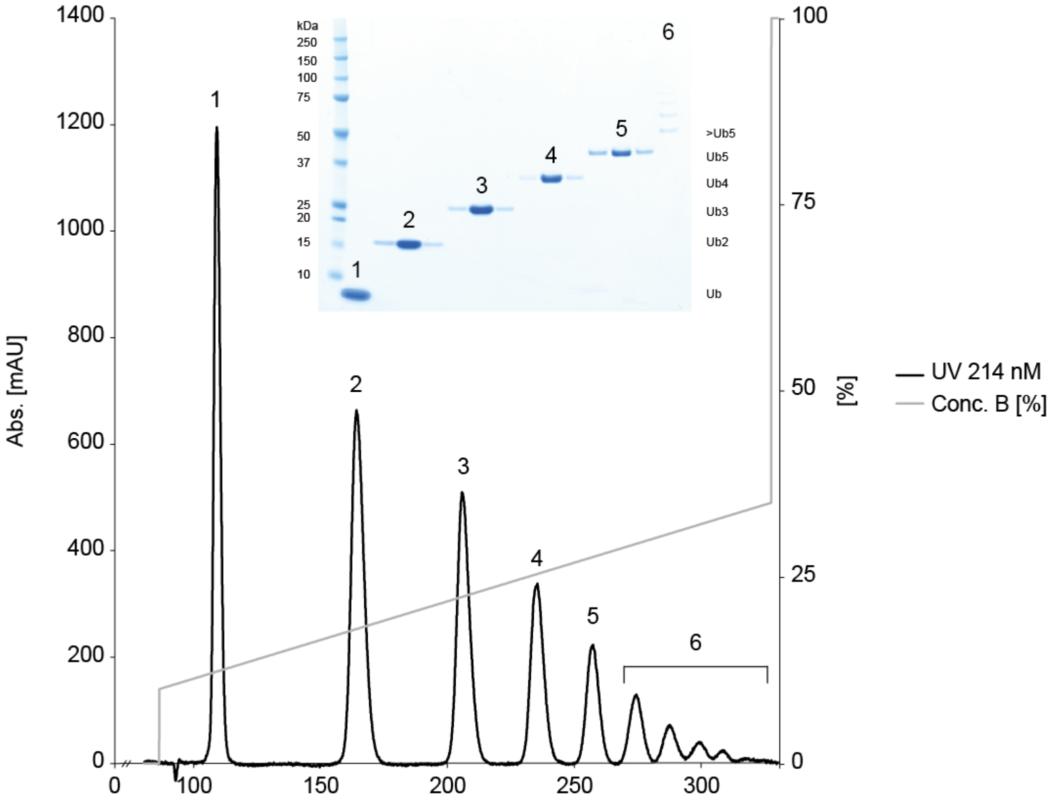

Figure S3, related to Figures 3 and 5. Example of ion-exchange chromatogram and purification of M1-linked Ub (pSer20) oligomers.
1: monomer, **2:** dimer, **3:** trimer, **4:** tetramer, **5:** pentamer, **6:** hexamer and higher molecular weight.

Supplemental Figure 4

A

M1				
**Phosphorylation**	–	**pSer20**	**pSer57**	**pSer65**
**Correct linkage**	96%	84%	94%	79%
**Other linkages**	–	9% K11	–	10% K33
**Deubiquitinase**	**% max**	**Activity on phosphorylated vs unphosphorylated dimers (%)**		
A20				
AMSH				
AMSH-LP				
CEZANNE				
CYLD	42 ± 7	20 ± 2	20 ± 4	92 ± 4
OTUB1				
OTUB2				
OTUD1				
OTUD3				
OTUD5				
OTULIN	92 ± 5	66 ± 5	41 ± 3	114 ± 5
TRABID				
USP1				
USP10				
USP15				
USP16	93 ± 4	61 ± 5	54 ± 7	88 ± 1
USP2	13 ± 8	76 ± 2	105 ± 8	0 ± 0
USP20				
USP21	97 ± 3	63 ± 12	45 ± 5	110 ± 3
USP25				
USP27x				
USP28				
USP30				
USP36				
USP4	31 ± 5	38 ± 5	39 ± 5	101 ± 3
USP5	100 ± 6	57 ± 8	40 ± 8	104 ± 4
USP6				
USP7				
USP8				
USP9x				
VCPIP1				

B

K6				
**Phosphorylation**	–	**pSer20**	**pSer57**	**pSer65**
**Correct linkage**	96%	89%	86%	77%
**Other linkages**	–	7% K11	–	11% K63
**Deubiquitinase**	**% max**	**Activity on phosphorylated vs unphosphorylated dimers (%)**		
A20				
AMSH				
AMSH-LP				
CEZANNE				
CYLD	95 ± 13	60 ± 4	41 ± 4	22 ± 1
OTUB1				
OTUB2				
OTUD1				
OTUD3	79 ± 5	84 ± 13	49 ± 2	91 ± 1
OTUD5				
OTULIN				
TRABID				
USP1	83 ± 6	83 ± 10	47 ± 4	33 ± 1
USP10	34 ± 8	72 ± 6	48 ± 3	25 ± 2
USP15	75 ± 2	57 ± 7	42 ± 4	0 ± 0
USP16	93 ± 5	76 ± 7	47 ± 3	127 ± 10
USP2	73 ± 10	75 ± 6	43 ± 3	9 ± 1
USP20	100 ± 3	60 ± 7	40 ± 9	41 ± 1
USP21	82 ± 4	72 ± 6	63 ± 1	123 ± 4
USP25	0			
USP27x	76 ± 15	48 ± 5	38 ± 6	0 ± 0
USP28	41 ± 4	53 ± 7	34 ± 2	13 ± 1
USP30				
USP36				
USP4	86 ± 8	70 ± 9	42 ± 6	43 ± 1
USP5	77 ± 8	68 ± 7	45 ± 4	111 ± 10
USP6	46 ± 9	43 ± 5	60 ± 3	0 ± 0
USP7	84 ± 5	69 ± 2	40 ± 1	0 ± 0
USP8	12 ± 7	127 ± 17	113 ± 7	0 ± 0
USP9x	16 ± 16	61 ± 10	60 ± 18	0 ± 0
VCPIP1				

C

K11				
**Phosphorylation**	–	**pSer20**	**pSer57**	**pSer65**
**Correct linkage**	95%	81%	95%	33%
**Other linkages**	–	Mix	–	62% K63
**Deubiquitinase**	**% max**	**Activity on phosphorylated vs unphosphorylated dimers (%)**		
A20				
AMSH				
AMSH-LP				
CEZANNE	48 ± 7	75 ± 9	88 ± 11	113 ± 2
CYLD	62 ± 25	69 ± 5	82 ± 3	19 ± 1
OTUB1				
OTUB2	21 ± 4	69 ± 3	71 ± 4	78 ± 1
OTUD1				
OTUD3	34 ± 9	136 ± 14	79 ± 9	39 ± 3
OTUD5				
OTULIN				
TRABID				
USP1	49 ± 4	96 ± 8	97 ± 9	28 ± 1
USP10	40 ± 3	90 ± 3	89 ± 10	28 ± 2
USP15	74 ± 3	90 ± 4	65 ± 5	11 ± 1
USP16	44 ± 4	111 ± 12	91 ± 18	97 ± 0
USP2	53 ± 17	80 ± 4	91 ± 5	8 ± 3
USP20	74 ± 2	51 ± 4	63 ± 2	72 ± 2
USP21	77 ± 8	82 ± 3	58 ± 3	92 ± 6
USP25	48 ± 7	71 ± 8	87 ± 14	11 ± 3
USP27x	18 ± 6	93 ± 8	71 ± 10	0 ± 0
USP28	43 ± 21	85 ± 8	103 ± 14	34 ± 1
USP30	64 ± 7	50 ± 5	29 ± 3	12 ± 8
USP36	100 ± 9	65 ± 9	49 ± 4	96 ± 3
USP4	41 ± 5	94 ± 13	127 ± 20	136 ± 15
USP5	47 ± 3	82 ± 4	106 ± 11	152 ± 9
USP6	39 ± 6	100 ± 10	119 ± 10	0 ± 0
USP7				
USP8	49 ± 6	74 ± 4	85 ± 5	0 ± 0
USP9x	61 ± 5	66 ± 3	54 ± 6	0 ± 0
VCPIP1	77 ± 4	71 ± 6	52 ± 5	52 ± 1

D

K29			
**Phosphorylation**	–	**pSer57**	**pSer65**
**Correct linkage**	–	75%	91%
**Other linkages**	–	17% K48	–
**Deubiquitinase**	**% max**	**Activity on phosphorylated vs unphosphorylated dimers (%)**	
A20			
AMSH			
AMSH-LP			
CEZANNE			
CYLD			
OTUB1			
OTUB2			
OTUD1			
OTUD3			
OTUD5			
OTULIN			
TRABID	100 ± 9	49 ± 4	98 ± 4
USP1			0
USP10			
USP15	33 ± 5	50 ± 3	3 ± 6
USP16	89 ± 8	32 ± 3	70 ± 2
USP2	23 ± 8	135 ± 11	0 ± 0
USP20	21 ± 17	76 ± 13	0 ± 0
USP21	83 ± 4	52 ± 2	90 ± 4
USP25			
USP27x			
USP28			
USP30			
USP36	30 ± 15	54 ± 8	0 ± 0
USP4	38 ± 4	73 ± 3	55 ± 3
USP5	82 ± 11	55 ± 6	96 ± 6
USP6			
USP7			
USP8			
USP9x			
VCPIP1			

E

K33				
**Phosphorylation**	–	**pSer20**	**pSer57**	**pSer65**
**Correct linkage**	99%	86%	99%	87%
**Other linkages**	–	9% K11	–	10% K63
**Deubiquitinase**	**% max**	**Activity on phosphorylated vs unphosphorylated dimers (%)**		
A20				
AMSH				
AMSH-LP				
CEZANNE				
CYLD	12 ± 16	92 ± 15	74 ± 4	0 ± 0
OTUB1				
OTUB2				
OTUD1				
OTUD3				
OTUD5				
OTULIN				
TRABID	51 ± 5	52 ± 5	55 ± 4	32 ± 1
USP1	100 ± 5	54 ± 5	37 ± 7	0 ± 0
USP10	13 ± 7	76 ± 10	54 ± 14	43 ± 8
USP15	37 ± 6	51 ± 5	47 ± 4	16 ± 1
USP16	39 ± 17	100 ± 9	62 ± 5	204 ± 4
USP2	44 ± 4	96 ± 17	73 ± 1	0 ± 0
USP20	21 ± 9	70 ± 8	59 ± 5	0 ± 0
USP21	69 ± 11	58 ± 3	46 ± 5	128 ± 2
USP25	38 ± 10	17 ± 2	14 ± 1	0 ± 0
USP27x				
USP28				
USP30	33 ± 10	56 ± 6	32 ± 16	59 ± 4
USP36	38 ± 8	97 ± 9	71 ± 11	81 ± 5
USP4	23 ± 7	60 ± 5	57 ± 6	11 ± 6
USP5	54 ± 4	66 ± 7	54 ± 4	103 ± 3
USP6	8 ± 8	24 ± 6	43 ± 19	0 ± 0
USP7	17 ± 11	71 ± 2	63 ± 1	0 ± 0
USP8	11 ± 16	64 ± 13	57 ± 8	0 ± 0
USP9x				
VCPIP1				

F

K48				
**Phosphorylation**	–	**pSer20**	**pSer57**	**pSer65**
**Correct linkage**	95%	80%	97%	86%
**Other linkages**	–	8% K6	–	6% K63
**Deubiquitinase**	**% max**	**Activity on phosphorylated vs unphosphorylated dimers (%)**		
A20	61 ± 8	48 ± 5	18 ± 2	34 ± 3
AMSH				
AMSH-LP				
CEZANNE	7			
CYLD	100 ± 8	39 ± 5	29 ± 1	17 ± 1
OTUB1	85 ± 4	41 ± 3	38 ± 2	179 ± 7
OTUB2	36 ± 9	36 ± 2	27 ± 3	121 ± 2
OTUD1				
OTUD3	89 ± 5	47 ± 2	32 ± 2	102 ± 15
OTUD5				
OTULIN				
TRABID				
USP1	93 ± 5	44 ± 3	37 ± 5	30 ± 1
USP10	66 ± 11	45 ± 2	26 ± 6	27 ± 1
USP15	88 ± 13	46 ± 9	34 ± 4	17 ± 1
USP16	83 ± 13	53 ± 8	47 ± 5	135 ± 9
USP2	80 ± 5	45 ± 3	39 ± 4	12 ± 1
USP20	88 ± 8	42 ± 6	40 ± 2	78 ± 3
USP21	96 ± 7	41 ± 5	39 ± 4	191 ± 13
USP25	78 ± 7	43 ± 2	27 ± 2	3 ± 1
USP27x	84 ± 20	45 ± 6	35 ± 3	0 ± 0
USP28	79 ± 4	42 ± 7	29 ± 3	34 ± 2
USP30	87 ± 9	34 ± 2	26 ± 6	48 ± 3
USP36	88 ± 6	51 ± 7	42 ± 5	54 ± 1
USP4	83 ± 8	50 ± 5	41 ± 4	122 ± 5
USP5	81 ± 11	41 ± 2	39 ± 2	133 ± 9
USP6	80 ± 10	45 ± 3	37 ± 4	0 ± 0
USP7	81 ± 10	45 ± 5	37 ± 3	12 ± 0
USP8	75 ± 5	53 ± 3	49 ± 4	0 ± 0
USP9x	41 ± 7	37 ± 2	28 ± 7	0 ± 0
VCPIP1	70 ± 12	35 ± 5	17 ± 2	112 ± 4

G

K63				
**Phosphorylation**	–	**pSer20**	**pSer57**	**pSer65**
**Correct linkage**	91%	93%	91%	94%
**Other linkages**	6% K6	–	5.5% K33	–
**Deubiquitinase**	**% max**	**Activity on phosphorylated vs unphosphorylated dimers (%)**		
A20				
AMSH	54 ± 3	64 ± 7	61 ± 5	0 ± 0
AMSH-LP	42 ± 10	53 ± 2	76 ± 9	20 ± 1
CEZANNE				
CYLD	75 ± 4	70 ± 6	52 ± 11	0 ± 0
OTUB1				
OTUB2	89 ± 4	47 ± 3	39 ± 4	83 ± 7
OTUD1	90 ± 10	54 ± 9	44 ± 12	10 ± 1
OTUD3	75 ± 6	93 ± 6	61 ± 7	8 ± 0
OTUD5	28 ± 6	72 ± 7	48 ± 1	0 ± 0
OTULIN				
TRABID	48 ± 6	38 ± 6	51 ± 2	0 ± 0
USP1	93 ± 5	74 ± 4	61 ± 7	0 ± 0
USP10	55 ± 4	91 ± 12	62 ± 8	21 ± 9
USP15	71 ± 3	88 ± 7	64 ± 5	0 ± 0
USP16	98 ± 13	93 ± 24	59 ± 7	94 ± 4
USP2	80 ± 5	76 ± 2	68 ± 8	27 ± 1
USP20	89 ± 10	78 ± 3	55 ± 9	0 ± 0
USP21	74 ± 6	93 ± 8	81 ± 13	93 ± 1
USP25	71 ± 3	56 ± 22	49 ± 4	0 ± 0
USP27x	73 ± 8	73 ± 2	56 ± 4	0 ± 0
USP28	74 ± 4	66 ± 9	56 ± 9	0 ± 0
USP30	32 ± 6	62 ± 6	56 ± 6	29 ± 5
USP36	53 ± 7	102 ± 5	79 ± 8	0 ± 0
USP4	80 ± 6	83 ± 8	53 ± 6	91 ± 3
USP5	77 ± 5	81 ± 4	68 ± 6	81 ± 2
USP6	83 ± 6	74 ± 2	70 ± 4	0 ± 0
USP7	100 ± 8	55 ± 3	36 ± 9	0 ± 0
USP8	53 ± 10	104 ± 16	95 ± 10	0 ± 0
USP9x	32 ± 5	50 ± 5	51 ± 8	0 ± 0
VCPIP1				

Figure S4, related to Figure 5. Quantification of DUB activity on phosphorylated ubiquitin dimers.

Purified dimers of Ub, Ub (pSer20), Ub (pSer57), and Ub (pSer65) from each linkage assembly reaction (**Figure 3**) were used to profile deubiquitnase activity using MALDI-TOF mass spectrometry with a ^{15}N-labelled Ub / phosphoubiquitin internal standard. **(A)** M1 Ub dimers, **(B)** K6 Ub dimers, **(C)** K11 Ub dimers, **(D)** K29 Ub dimers, **(E)** K33 Ub dimers, **(F)** K48 Ub dimers, **(G)** K63 Ub dimers. Relevant quantities of other linkages are indicated. The activity of each DUB is normalised to the activity of the most active DUB within the set of active DUBs (against a specific linkage) and displayed as yellow bars. The activity of the DUBs towards phosphorylated Ub dimers is expressed in % of the activity towards unmodified Ub dimers and colour coded from blue (no cleavage) to white (same activity as on unmodified Ub) to red (increased cleavage compared to unmodified Ub).

Supplemental Figure 5

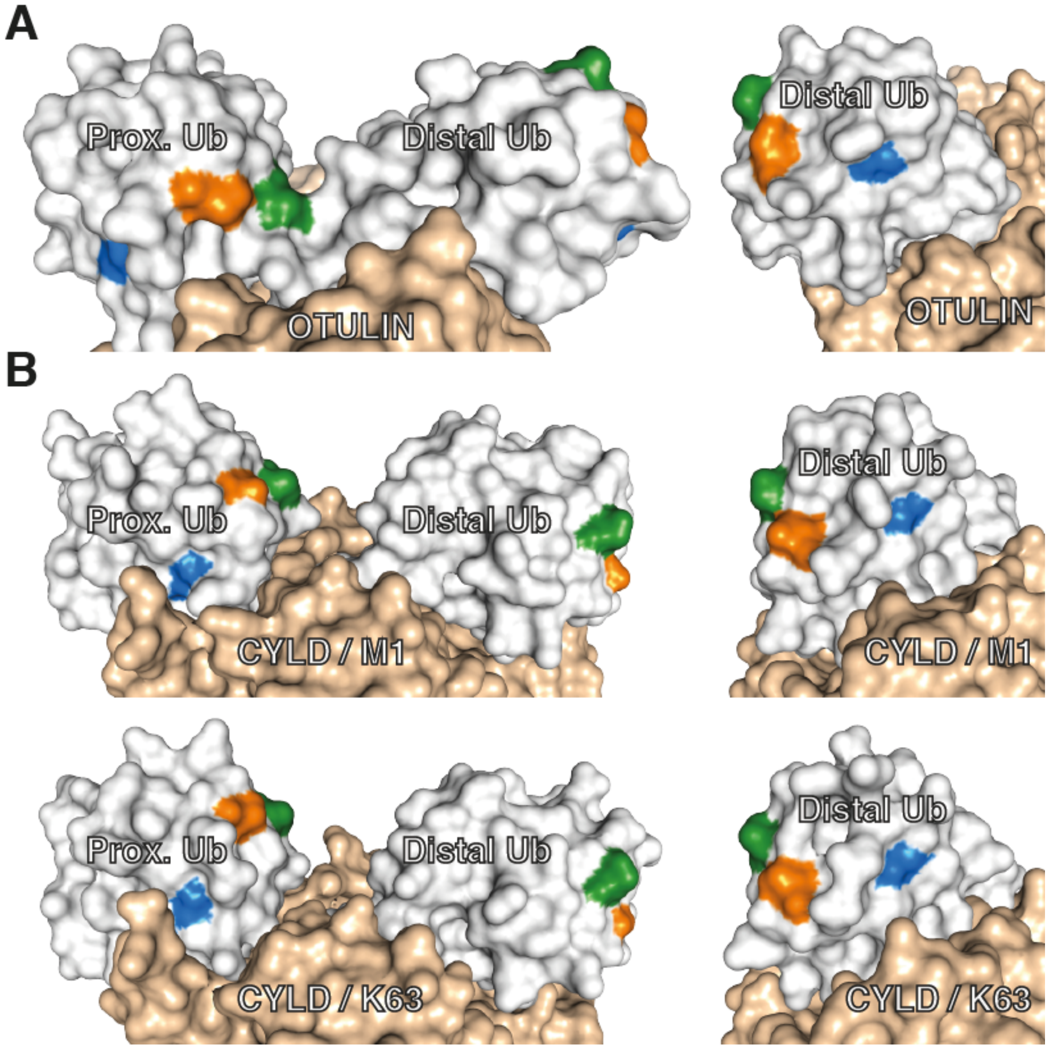

Figure S5, related to Figure 7. Structural models for the control of deubiquitinase specificity by phosphorylation.
(A) Structure of OTULIN in complex with a linear Ub dimer (PDB ID: 3ZNS (Keusekotten et al., 2013)). The position of each phosphorylation on the proximal and distal Ub is colour coded (**Figure 7A**). **(B)** Structure of CYLD in complex with a linear Ub dimer (PDB ID: 3WXF (Sato et al., 2015)) and a K63-linked ubiquitin dimer (PDB: 3WXG (Sato et al., 2015)). The position of each phosphorylation on the proximal and distal Ub is colour coded.

2. Supplemental Tables and Legends

Table S1, related to Figure 1. Data collection and refinement statistics for Ub (20pSer)

Table S2, related to Figures 2, 3 and 5. List of E2, E3 and DUB constructs used.

Table S3, related to Figure 5. DUB dilution table.

Supplemental Table 1, related to Figure 1. Data collection and refinement statistics for Ub (20pSer)

Ub20pSer	
**Wavelength [Å]**	0.96999
**Resolution range [Å]**	33.5 - 1.55 (1.58 - 1.55)
**Space group**	P 31 2 1
**Unit cell (a, b, c) (α, ß, γ) [°]**	(49.5205 49.5205 53.642) (90 90 120)
**Total reflections**	11448
**Unique reflections**	10779
**Multiplicity**	3.5 (3.2)
**Completeness (%)**	95.3 (91.3)
**Mean I/σ(I)**	6.4 (1.9)
**Wilson B-factor**	19.596
**Rmerge (all I+ and I-)**	0.102 (0.578)
**R-meas**	0.12 (0.69)
**Reflections used in refinement**	10754
**R-work**	0.1902
**R-free**	0.2194
**Number of non-hydrogen atoms**	691
Protein	638
**Protein residues**	76
**RMS(bonds)**	0.015
**RMS(angles)**	1.421
**Ramachandran favored (%)**	97.44
**Ramachandran allowed (%)**	2.56
**Ramachandran outliers (%)**	0
**Rotamer outliers (%)**	1.4
**Clashscore**	5.35
**Average B-factor**	27.32
Macromolecules	26.28
Solvent	39.82

Supplemental table 3, related to Figure 5. DUB dilution table.

**USP1**	**USP2**	**USP6**	**OTUB2**
240 ng/ul, K63	60 ng/ul, K63	3 ng/ul, K63	30 ng/ul, K63
**USP8**	**USP5**	**USP20**	**OTUD1**
144 ng/ul, K63	24 ng/ul, K63	60 ng/ul, K63	6 ng/ul, K63
**CYLD**	**OTUD5**	**AMSH**	**AMSH-LP**
240 ng/ul, K63	300 ng/ul, K63	60 ng/ul, K63	24 ng/ul, K63
**USP7**	**USP27x**	**Cezanne**	**USP21**
30 ng/ul, K11	120 ng/ul, K11	12 ng/ul, K11	58.4 ng/ul,K11
**USP9x**	**USP28**	**OTUD3**	**USP25**
170 ng/ul, K11	60 ng/ul, K11	60 ng/ul, K11	30 ng/ul, K11
**USP10**	**USP36**	**USP30**	**Otulin**
240 ng/ul, K11	750 ng/ul, K11	430 ng/ul, K48	1.2 ng/ul, M1
**VCPIP**	**A20**	**TRABID**	**OTUB1**
500 ng/ul, K48	60 ng/ul, K48	240 ng/ul, K48	300 ng/ul, K48
**USP4**	**USP16**	**USP15**	
120 ng/ul, K48	60 ng/ul, K48	16 ng/ul, K48	

3. Supplemental Data 1

Supplemental Data 1, related to Figure 3 and 5. Targeted mass spectrometry data and peptide sequences.

Supplemental Data 1.1 related to Figure 3 and 5. Targeted mass spectrometry using AQUA peptides on purified trimers.
M1 (assembled with Ube1, UBE2L3 and HOIP) on either Ub **(A)**, Ub (pSer20) **(B)**, Ub (pSer57) **(C)** or Ub (pSer65) **(D).**

Supplemental Data 1.2 related to Figure 3 and 5. Targeted mass spectrometry using AQUA peptides on purified trimers.
K6 chains (assembled with Ube1, UBE2L3, NleL) on either UbK48R **(A)**, UbK48R (pSer20) **(B)**, UbK48R (pSer57) **(C)** or UbK48R (pSer65) **(D)**.

Supplemental Data 1.3 related to Figure 3 and 5. Targeted mass spectrometry using AQUA peptides on total reactions.
Reactions with Ube1, UBE2L3 and NleL with either Ub **(A)**, Ub (pSer20) **(B)**, Ub (pSer57) **(C)** or Ub (pSer65) **(D).**

Supplemental Data 1.4 related to Figure 3 and 5. Targeted mass spectrometry using AQUA peptides on purified trimers.
K11 chains (assembled with Ube1, UBE2S-UBP and AMSH) on either Ub **(A)**, Ub (pSer20) **(B)**, Ub (pSer57) **(C)** or Ub (pSer65) **(D).**

Supplemental Data 1.5 related to Figure 3 and 5. Targeted mass spectrometry using AQUA peptides on purified trimers.
K29 chains (assembled with Ube1, UBE2D3, UBE3C and vOTU) on either Ub **(A)**, Ub (pSer57) **(B)** or Ub (pSer65) **(C)**.

Supplemental Data 1.6 related to Figure 3 and 5. Targeted mass spectrometry using AQUA peptides on total reactions.
Reactions with Ube1, UBE2D3 and UBE3C with either Ub **(A)**, Ub (pSer20) **(B)**, Ub (pSer57) **(C)** or Ub (pSer65) **(D).**

Supplemental Data 1.7 related to Figure 3 and 5. Targeted mass spectrometry using AQUA peptides on purified trimers.
K33 chains (assembled with Ube1, UBE2D1, AREL1, OTUB1 and Cezanne-EK) on either Ub **(A)**, Ub (pSer20) **(B)**, Ub (pSer57) **(C)** or Ub (pSer65) **(D).**

Supplemental Data 1.8 related to Figure 3 and 5. Targeted mass spectrometry using AQUA peptides on purified trimers.
K48 chains (assembled with Ube1, UBE2R1 or UBE2K) on either Ub **(A)**, Ub (pSer20) **(B)**, Ub (pSer57) **(C)**, Ub (pSer65) with UBE2R1 **(D)** or with UBE2K **(E).**

Supplemental Data 1.9 related to Figure 3 and 5. Targeted mass spectrometry using AQUA peptides on purified trimers.

K63 chains (assembled with Ube1, UBE2N and UBE2V1) on either Ub **(A)**, Ub (pSer20) **(B)**, Ub (pSer57) **(C)** or Ub (pSer65) **(D)**.

Supplemental Data 1.10 related to Figure 3 and 5. Targeted mass spectrometry using AQUA peptides on total reactions.
Reaction with Ube1, UBE2L3 and HHARI with either Ub **(A)**, Ub (pSer20) **(B)**, Ub (pSer57) **(C)** or Ub (pSer65) **(D)**.

Supplemental Data 1.11 related to Figure 3 and 5. List of isotopically labelled peptides used to monitor polyUb and phosphorylated polyUb linkages.
Signature peptides representing isopeptide-linked polyUb linkages are denoted by a subscript "GG" adjacent to the modified lysine. Signature peptides representing phosphorylated peptides are denotes by a "p" adjacent to modified serine. Isotopically labelled amino acids are denoted in blue and the oxidation state of Met-containing peptides is denoted by "ox". For each peptide, the optimal precursor ions to the HCD collision energy of 30% were selected for the PRM analysis.

4. Supplemental Experimental Procedures

E2 charging assay

E2-charging assays were carried out in 1 mL reactions by incubating 0.21 µM UBE1, 5 µM E2 and 5.8 µM Ub in 50 mM HEPES pH 7.4, 7.5 mM NaCl, 2 mM magnesium acetate, and 0.2 mM ATP. Reactions were incubated for 5 or 60 min at 30°C and stopped by addition of non-reducing LDS sample buffer (Invitrogen). Samples were analyzed by SDS-PAGE using 4–12% Bis-Tris gels (Invitrogen) and visualised using InstantBlue (Expedeon).

Ub chain assembly and purification

Ub chains were assembled in buffer containing 40 mM Tris pH 7.5, 10 mM $MgCl_2$, 0.6 mM DTT and 10 mM ATP. M1 chains: 0.8 mM Ub, 1 µM UBE1, 10 µM UBE2L3 and 10 µM HOIP. K6 chains: 1 mM UbK48R, 0.5 µM UBE1, 9.5 µM UBE2L3 and 12.4 µM NleL. K11 chains: 1.2 mM Ub, 1 µM UBE1, 40 µM UBE2S-UBP and 2 µM AMSH (added after 6 h). K29 chains: 1 mM Ub, 0.64 µM UBE1, 9.5 µM UBE2D3, 3 µM UBE3C and 2 µM vOTU. K33 chains: 1.1 mM Ub, 0.5 µM UBE1, 9 µM UBE2D1, 6.2 µM KIAA0317 and 5 µM OTUB1 + 20 µM Cezanne EK (added after 6 h). K48 chains: 0.95 mM Ub, 1 µM UBE1, 25 µM UBE2R1 or 25 µM UBE2K. K63 chains: 0.9 mM Ub, 1 µM UBE1, 10 µM UBE2N and 20 µM UBE2V1. Reactions were incubated at 30°C and terminated either by addition of reducing LDS sample buffer (for time course experiments) or dilution in 50 mM ammonium acetate pH 4.5 (for further purification). For time course experiments, samples were analyzed by SDS-PAGE. For Ub chains purification, the reactions were diluted in 50 mM ammonium acetate, pH 4.5 (to precipitate the enzymes) and filtered through a 0.22 µm PES membrane before loading on an ion exchange chromatography (ResourceS column, GE Healthcare). Ub chains were eluted using a gradient from 0 to 1 M NaCl in 50 mM ammonium acetate pH 4.5. Purity of each oligomer was assessed by running the peak edge and centre fractions on SDS-PAGE gels. Pure fractions for each oligomer were pooled, concentrated and buffer exchanged with 20 mM tris-HC pH 7.5.

Purification of deubiquitinases, E1s, E2s and E3s and DUBs

E1 and E2s: UBE1 was expressed as a 6His-tagged fusion protein in Sf21 cells and purified using Ni-NTA agarose. UBE2J2 and UBE2Z were expressed as a GST-tagged fusion protein in BL21 cells and affinity purified via its tag. All other E2 conjugating enzymes were expressed as His-tagged fusion proteins in BL21 cells and affinity purified over Ni-NTA-agarose **(Table S2A)**.
Expression and purification of E3 ligases: AREL1, HOIP, NleL and HHARI were expressed as GST-fusion proteins in BL21 DE3 cells, purified over GSH-agarose and recovered by protease treatment (TEV-protease or Prescission Protease) to sever to bond to the GST-tag. UBE3C (641 –end) was expressed as a GST-tagged fusion protein in Sf21 cells and essentially purified like the other ligases **(Table S2B)**.
Expression of DUB enzymes was performed as previously described (Ritorto et al., 2014). The proteins were produced in BL21 DE3 cells or Sf21 insect cells using standard procedures **(Table S2C)**.

Expression and purification of ^{15}N-Ub (pSer65)

Untagged full-length human Ub (1-76) was cloned into the pET-24 vector (DU20027) and expressed in E. coli BL21 (DE3) cells grown in 50 mM Na_2HPO_4, 22

mM KH_2PO_4, 8.5 mM NaCl, 1 g/L (18.3 mM) $^{15}NH_4Cl$, 3 g/L glucose, 0.1 mM $CaCl_2$, 1 mM $MgSO_4$, 5 µM $ZnCl_2$, 1 x MEM Vitamin solution (Life Technologies), trace minerals, 50 µg/L kanamycin. The cells were grown to OD_{600} = 0.6 and induced with 1 mM IPTG for 3h at 37ºC. The cells were collected in MilliQ water and frozen in liquid nitrogen. After thawing, sonicating and sedimenting the insoluble material the soluble phase was diluted with MilliQ water to 80 mL/L equivalent of culture. The pH was adjusted to 4.5 with perchloric acid and the proteins were left to precipitate at 4ºC overnight. The insoluble phase was sedimented by centrifugation at 38000 x *g* for 20 min. The supernatant containing Ub and a small amount of contaminants was subjected to chromatography on a Source 15 S column (8 mL bed) in 10 mM Ammonium acetate (pH 4.5) and eluted with a gradient of NaCl in 10 mM Ammonium acetate. The equivalent of 1 L culture was separated in each run. Ub elutes at around 20 mS/cm conductivity. The protein was concentrated and washed in 10 mM HEPES pH 7.5 using Millipore filter Unit (3000 MW cut off). 5 mg of ^{15}N-Ub was phosphorylated with 0.2 mg GST-PINK1 (pediculus humanus) at 30ºC in the presence of 50 mM Tris pH 7.5, 50 mM NaCl, 0.5 mM ATP, 10 mM $MgCl_2$. The kinase was removed with Q-Sepharose FF, and the phospho Ub was desalted and purified to homogeneity on a MonoQ column. Phospho-Ub can bind to MonoQ in 5 mM Tris pH 7.5 (conductivity < 2 mS/cm) and eluted with a shallow gradient of NaCl at about 7 mS/cm. Unphosphorylated Ub does not bind to Q at this pH.

MALDI TOF DUB assays

Analysis of DUB assays was performed similar to described previously (Ritorto et al., 2014), 31 human DUBs were freshly diluted in the reaction buffer (40 mM Tris–HCl, pH 7.6, 5 mM DTT, 0.005% (w/v) BSA) at different concentrations **(Table S3)**. All diubiquitin isomers (phosphorylated and unmodified) were diluted to 0.2 µg/µl in the Ub dimer buffer (40 mM Tris–HCl, pH 7.6, 0.005% (w/v) BSA) and used as substrates at a fixed concentration (1.5 µM). Reaction buffer, DUBs and Ub dimers were aliquoted in a 384 well plate; the plate was sealed and kept in ice until use. A Mosquito nanoliter pipetting system (Labcyte, Sunnyvale, CA, USA) was programmed to aliquot 1.92 µL of reaction buffer and 1.2 µL of enzyme in duplicate from a new master plate. Enzymes were pre-incubated in the reaction buffer for 5 min and afterwards 0.48 µL of diubiquitin topoisomers were added to the reaction mixture with a 3 times mixing cycle to assure homogeneity within the reaction volume. The reaction was sealed and incubated for 60 min at room temperature. The reaction was stopped by adding 10% TFA to a final concentration of 2% (v/v). Pipetting time was calculated in order to avoid incubation time biases between the first and the last column of the plate. 1.050 µL of each reaction was transferred to a new plate and spiked with 0.15 µL of 16 µM ^{15}N-labelled unmodified or Ser65 phosphorylated Ub as internal standard and mixed 1:1 with freshly prepared 2,5-dihydroxyacetophenone (DHAP) matrix (7.6 mg of 2,5-DHAP in 375 mL ethanol and 125 mL of an aqueous 12 mg/mL diammonium hydrogen citrate). Sample and matrix were mixed 20 times and 200 nL of the mixture was spotted in duplicate onto MTP AnchorChip 1,536 TF targets (600 mm anchor, Bruker Daltonics).

Mass spectrometry data was acquired on an UltrafleXtreme MALDI-TOF mass spectrometer (Bruker Daltonics) with Compass 1.3 control and processing software. The sample carrier was taught before each analysis to optimize and centre laser shooting. Internal calibration was performed before each analysis using the ^{15}N-Ub peak $[M+H]^+$ average = 8,569.3). Samples were run in automatic mode (AutoXecute, Bruker Daltonics). Ionization was achieved by a 2-kHz smartbeam-II solid state laser

with a fixed initial laser power of 60% (laser attenuator offset 68%, range 30%) and detected by the FlashDetector at detector gain of x10. Reflector mode was used with optimized voltages for reflector-1 (26.45 kV) and reflector-2 (13.40 kV), ion sources (IonSource-1: 25.0 kV, IonSource-2: 22.87 kV) and pulsed ion extraction (320 ns). An amount of 3,500 shots were summed up in 'random walk' and with 'large' smartbeam laser focus. Spectra were automatically calibrated on the ^{15}N-Ub m/z and processed using smoothing (Savitzky–Golay algorithm) and baseline subtraction ('TopHat') for reproducible peak annotation on non-resolved isotope distributions: one cycle, 0.2 m/z for the width. For area calculation, the complete isotopic distribution was taken into account. For comparison between phosphoubiquitin isomers, equal ionization efficiencies in MALDI were assumed. An in-house made script was used to report ^{15}N/phospho-^{15}N and monoubiquitin/phospho-monoubiquitin areas; plotting of graphs, calculation of standard deviation and coefficient of variation (%) were processed in Microsoft Excel.

Targeted mass spectrometry using AQUA peptides

Sample preparation
10 µg of each purified Ub trimers as well as 10 µg of K63-linked Ub dimer phosphorylated at position 65 were run on SDS-PAGE gels and briefly stained using InstantBlue (Expedeon). The bands were excised and cut into small pieces. Gel pieces were sequentially washed in water, 50% (v/v) acetonitrile, 0.1 M ammonium bicarbonate and overnight in 50% (v/v) acetonitrile in 25 mM ammonium bicarbonate at 4˚C. Gel pieces were dehydrated by addition of acetonitrile and solvents evaporated using a SpeedVac Concentrator (Eppendorf). The gel pieces were then rehydrated in 25 mM triethylammonium bicarbonate buffer pH 8.5 (Sigma-Aldrich) containing 4 µg of trypsin (Pierce) per 10 µg of Ub and incubated overnight at 30˚C with intermittent shaking. An equivalent volume of acetonitrile was added to the solution and incubated at room temperature for an additional 15 min. The solution was transferred to a new tube, flash frozen, and sublimated using a SpeedVac Concentrator. Simultaneously the gel pieces were incubated with 50% (v/v) acetonitrile in 2.5% (v/v) formic acid. This solution was added to the lyophilized first extract, flash frozen and evaporated using a SpeedVac Concentrator.

Preparation of Ub-AQUA Peptide Mixtures
Concentrated stocks of isotopically labelled internal standard (heavy) peptides and light synthetic peptides (M1, K6, K11, K27, K29, K33, K48, K63) were purchased from Cell Signaling Technologies, whereas phosphorylated and GG modified peptides were purchased from CRB Cambridge Research biochemical **(Data S1)**. All stock solutions were stored at -80°C, working stock solutions of individual peptides were prepared at 25 pmol/µL in 2% (v/v) ACN, 0.1% (v/v) FA and used to prepare an experimental mixture consisting of 25 peptides at 1 pmol/µL in 2% (v/v) ACN, 0.1% (v/v) FA. Experimental mixtures were frozen at -80°C in duplicate use aliquots for direct addition to samples.

Absolute quantitation by Parallel Reaction Monitoring (PRM)
PRM quantitation was performed on an Orbitrap Fusion mass spectrometer (Thermo-Fisher Scientific) with an Easy-Spray source coupled to an Ultimate 3000 Rapid Separation LC system (Thermo Fischer Scientific). Samples were loaded via a 5 µL full loop injection directly onto an EASY-Spray column (15 cm x 75 µm ID, PepMap

C18, 3 µm particles, 100 Å pore size, Thermo-Fisher Scientific) and separated by reverse phase chromatography at a flow rate of 1.00 µL/min where solvent A was 98% (v/v) H_2O, 2% (v/v) ACN, (v/v) 0.1% FA and solvent B was 98% (v/v) ACN, 2% (v/v) H_2O, 0.1% (v/v) FA. Upon LC direct injection, peptides were resolved with an isocratic gradient of 0.1% of solvent B over 10 min, followed by a step from 0.1% to 25.5% of solvent B over 41 min, 5 min of high organic wash (90% solvent B) and 12 min re-equilibration at 0.1% of solvent B. The Orbitrap Fusion mass spectrometer was operated in targeted mode "tMS2" for the detection of light and synthetic heavy peptides. The included m/z values were selected by the quadrupole, with 4 m/z isolation window, a maximum injection time of 100 ms and a maximum AGC target of 5e4. HCD fragmentation was performed at 30% collision energy for all included peptides and MS/MS fragments were detected in the Orbitrap mass analyzer at a FMWH resolution of 30,000 (at m/z 200). Peak integration of MS/MS spectra and quantification of Ub peptides were performed on Skyline (version 3.5.0.9191) (http://proteome.gs.washington.edu/software/skyline) (MacLean et al., 2010). Confident peak integration corresponding to the extracted ion chromatogram of MS/MS fragments for a selected precursor was adjusted manually to avoid potential interferences and quantitation was performed by integrating areas of peaks corresponding to the endogenous and heavy *m/z* values.

5. Supplemental References

Keusekotten, K., Elliott, P.R., Glockner, L., Fiil, B.K., Damgaard, R.B., Kulathu, Y., Wauer, T., Hospenthal, M.K., Gyrd-Hansen, M., Krappmann, D., et al. (2013). OTULIN antagonizes LUBAC signaling by specifically hydrolyzing Met1-linked polyubiquitin. Cell *153*, 1312–1326.

MacLean, B., Tomazela, D.M., Shulman, N., Chambers, M., Finney, G.L., Frewen, B., Kern, R., Tabb, D.L., Liebler, D.C., and MacCoss, M.J. (2010). Skyline: an open source document editor for creating and analyzing targeted proteomics experiments. Bioinformatics *26*, 966–968.

Ritorto, M.S., Ewan, R., Perez-Oliva, A.B., Knebel, A., Buhrlage, S.J., Wightman, M., Kelly, S.M., Wood, N.T., Virdee, S., Gray, N.S., et al. (2014). Screening of DUB activity and specificity by MALDI-TOF mass spectrometry. Nat Comms *5*, 4763.

Sato, Y., Goto, E., Shibata, Y., Kubota, Y., Yamagata, A., Goto-Ito, S., Kubota, K., Inoue, J.-I., Takekawa, M., Tokunaga, F., et al. (2015). Structures of CYLD USP with Met1- or Lys63-linked diubiquitin reveal mechanisms for dual specificity. Nat Struct Mol Biol *22*, 222–229.
