# Supplementary material for: Synthesis of Isomeric Phosphoubiquitin Chains Reveals that Phosphorylation Controls Deubiquitinase Activity and Specificity
Source: Cell Rep. 2016 Jul 14;16(4):1180–93. doi: 10.1016/j.celrep.2016.06.064 (PMC4967478; doi:10.1016/j.celrep.2016.06.064)
Supplement: Data S1. Targeted MS Data and Peptide Sequences, Related to Figures 3 and 5 [file mmc3.pdf]

Supplemental Data 1.1

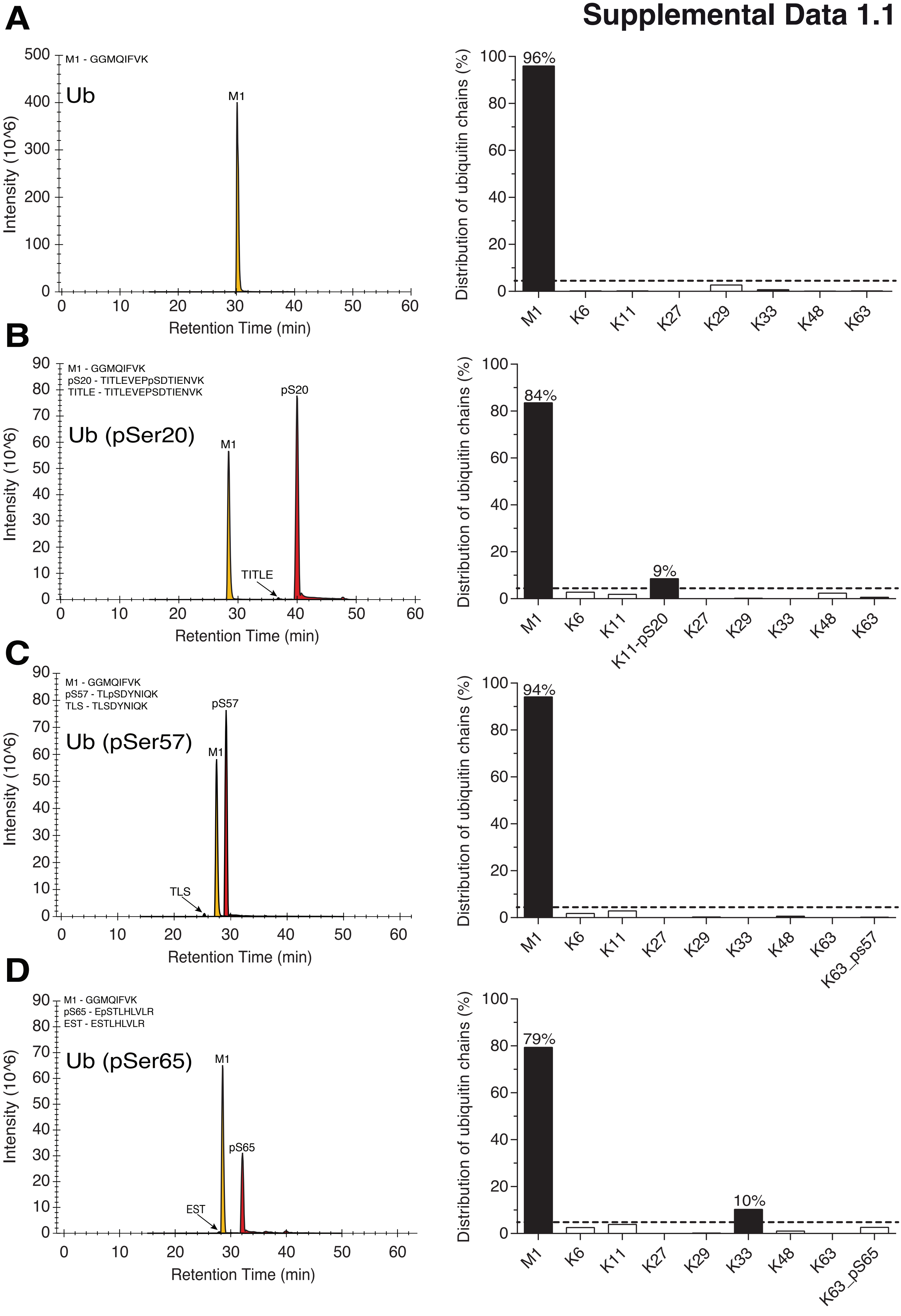

Supplemental Data 1.2

A

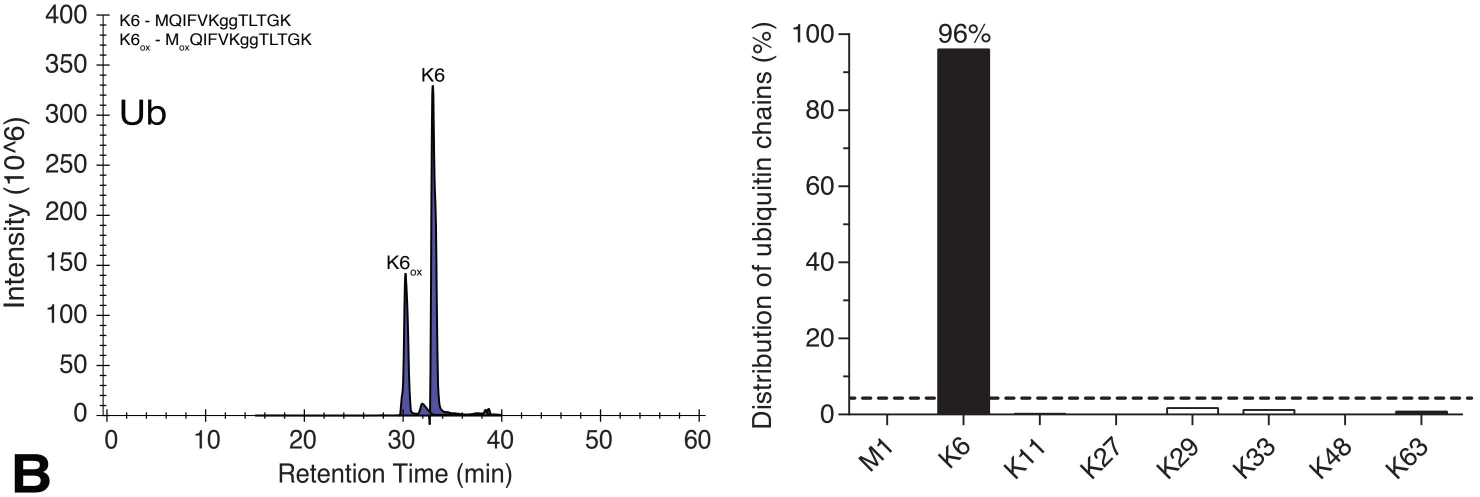

B

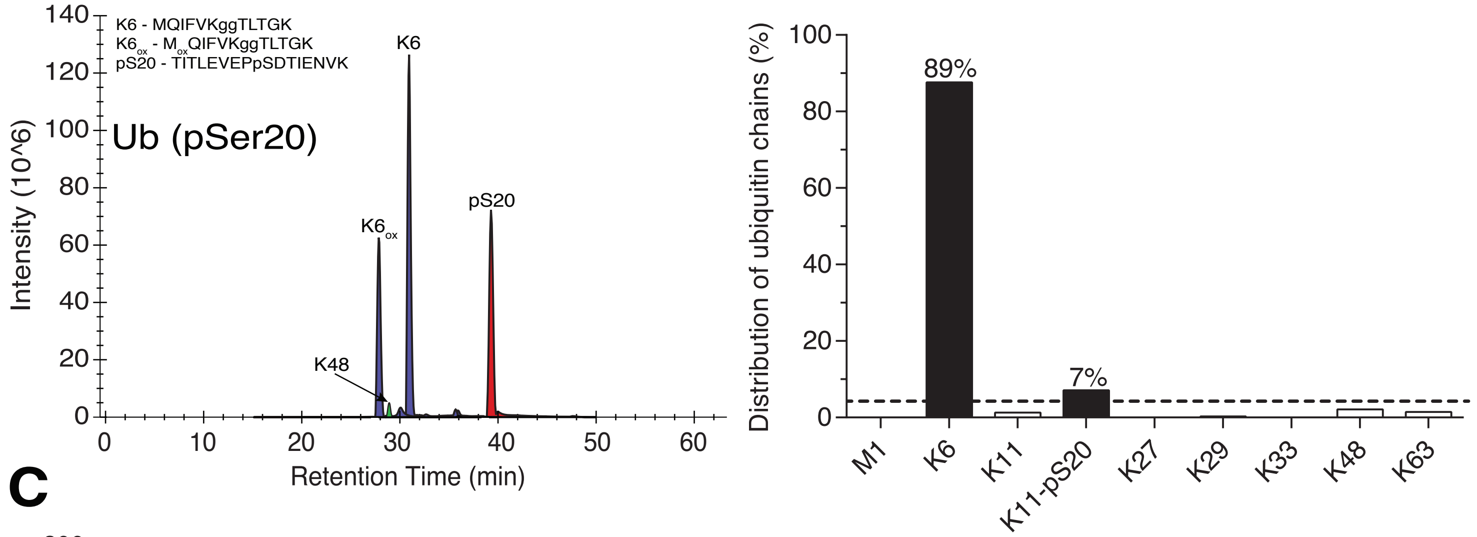

C

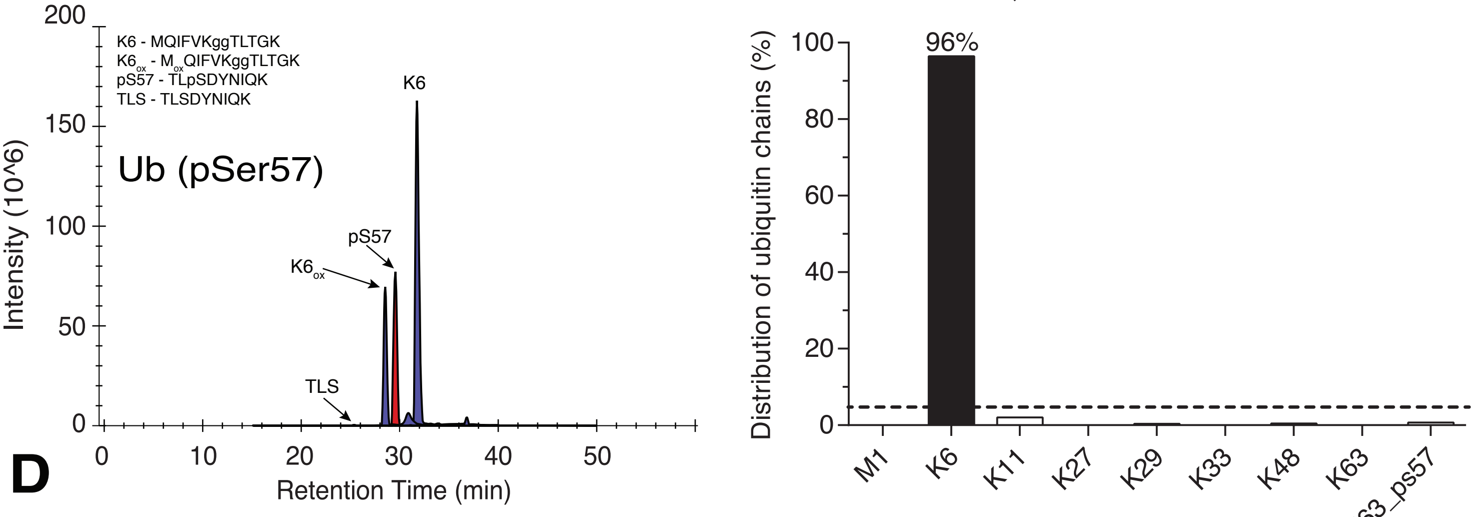

D

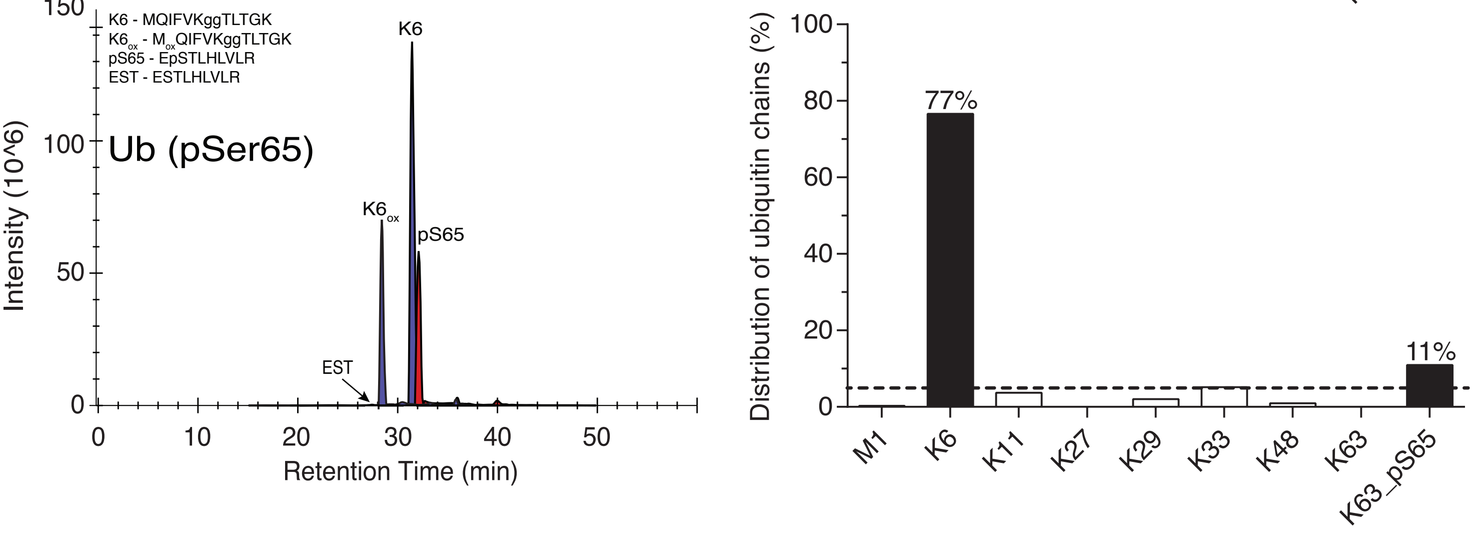

Supplemental Data 1.3

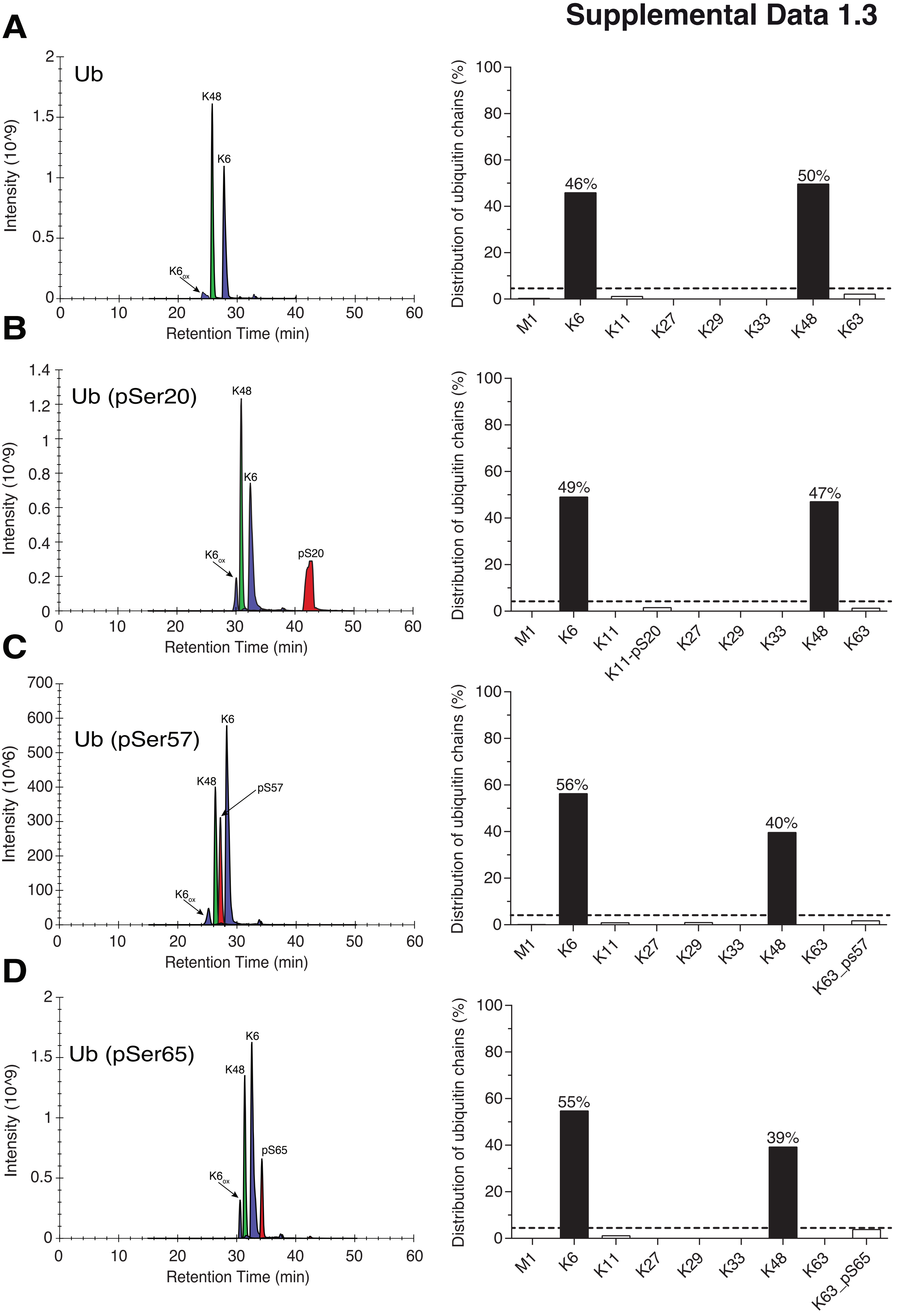

Supplemental Data 1.4

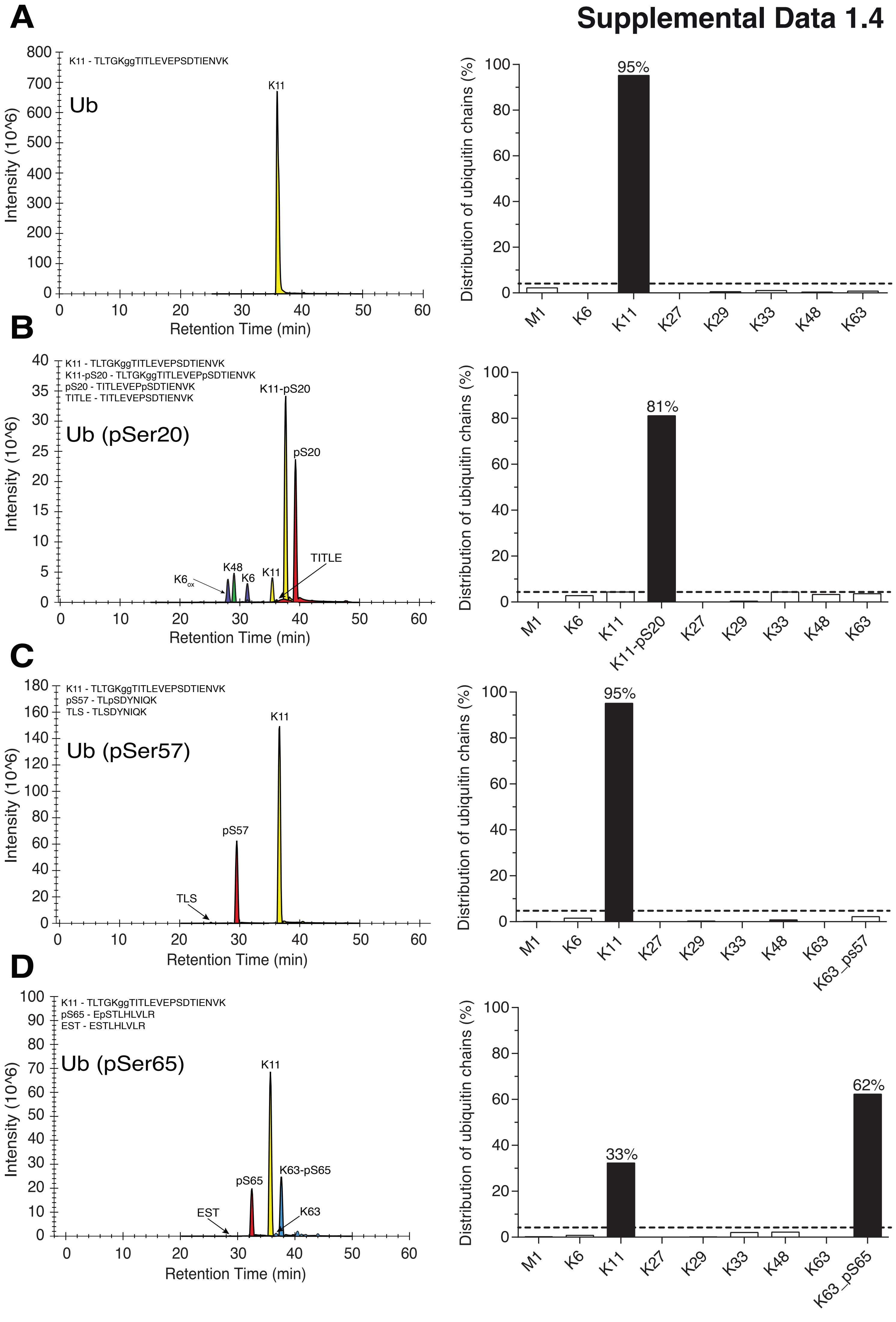

Supplemental Data 1.5

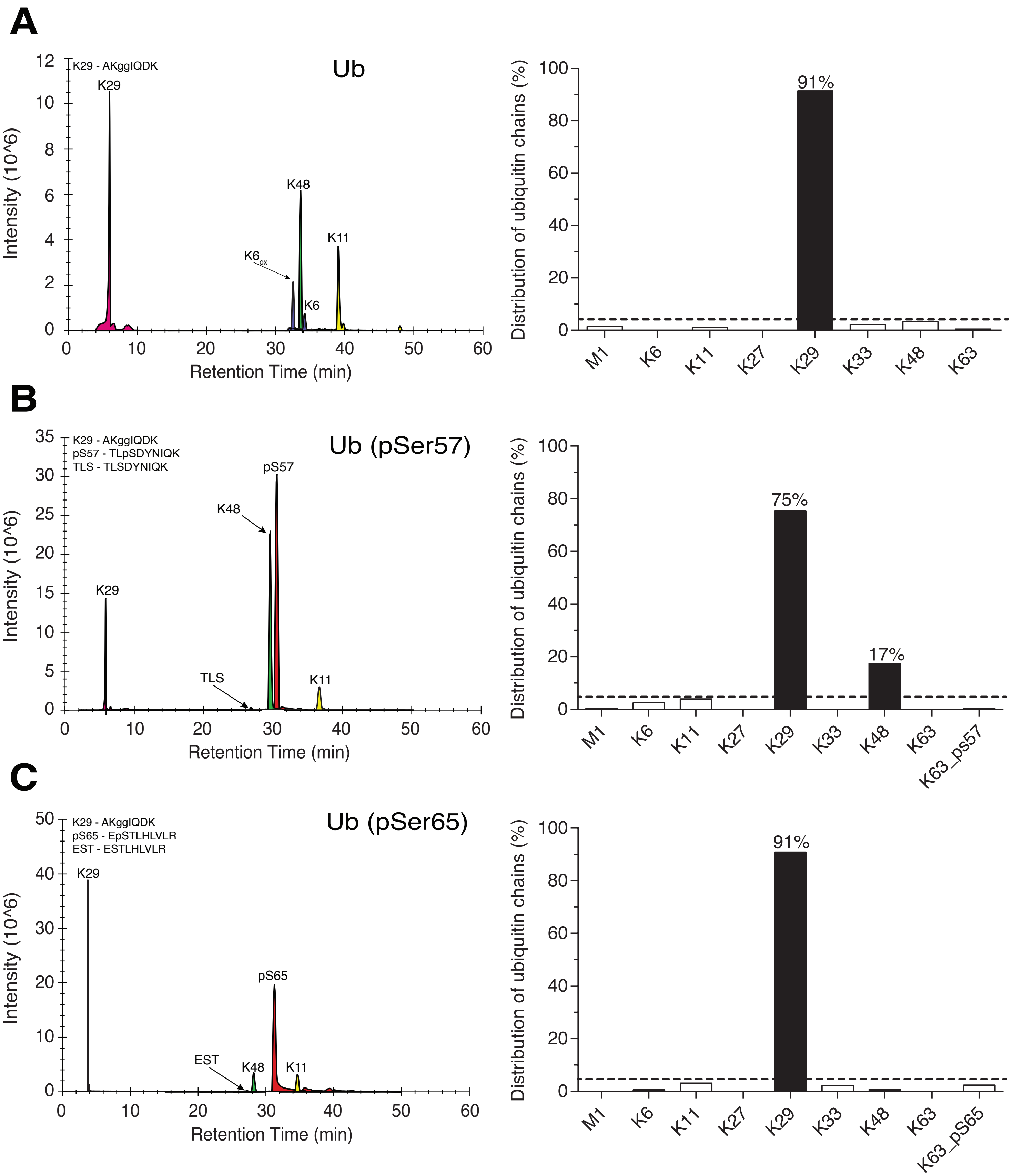

Supplemental Data 1.6

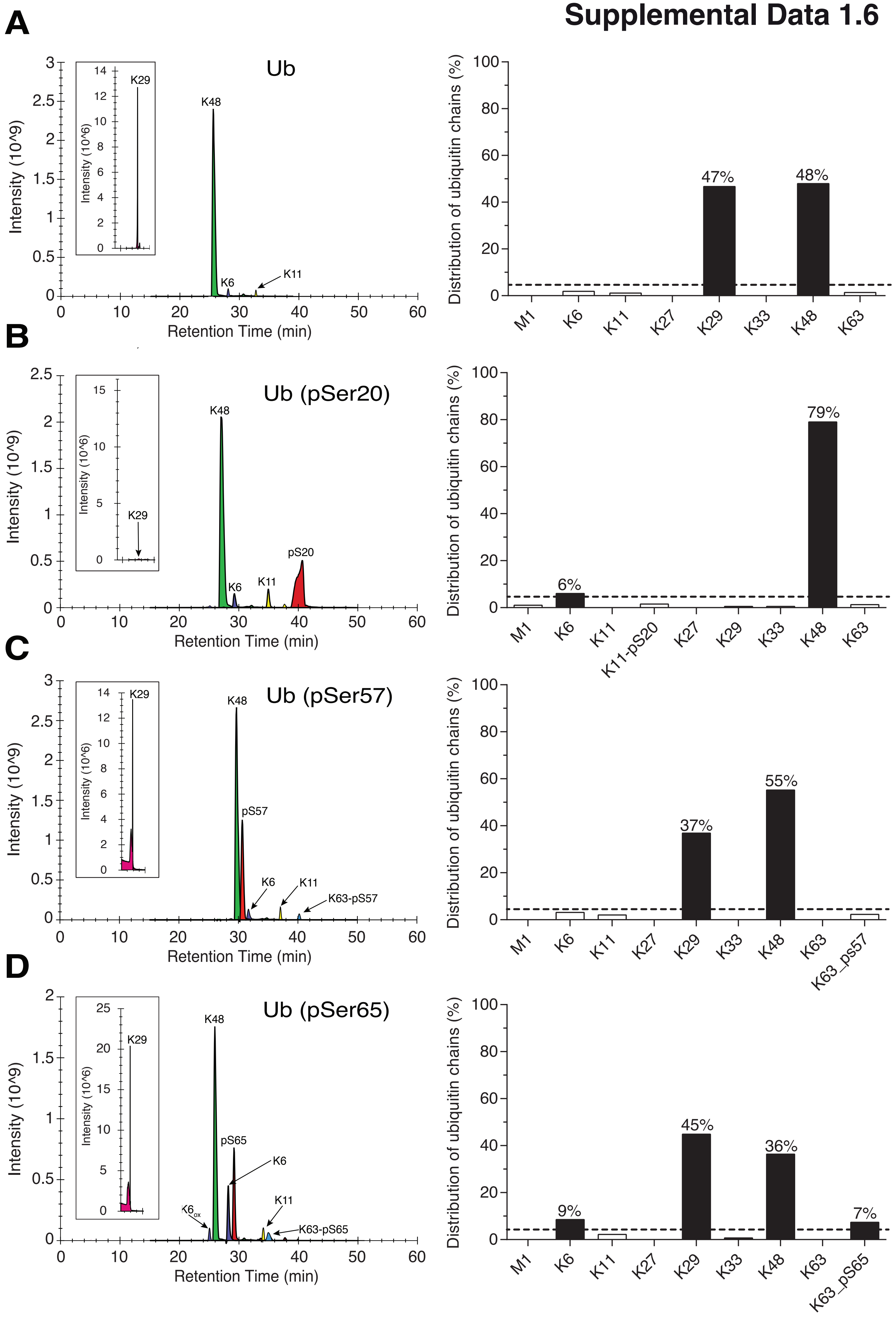

Supplemental Data 1.7

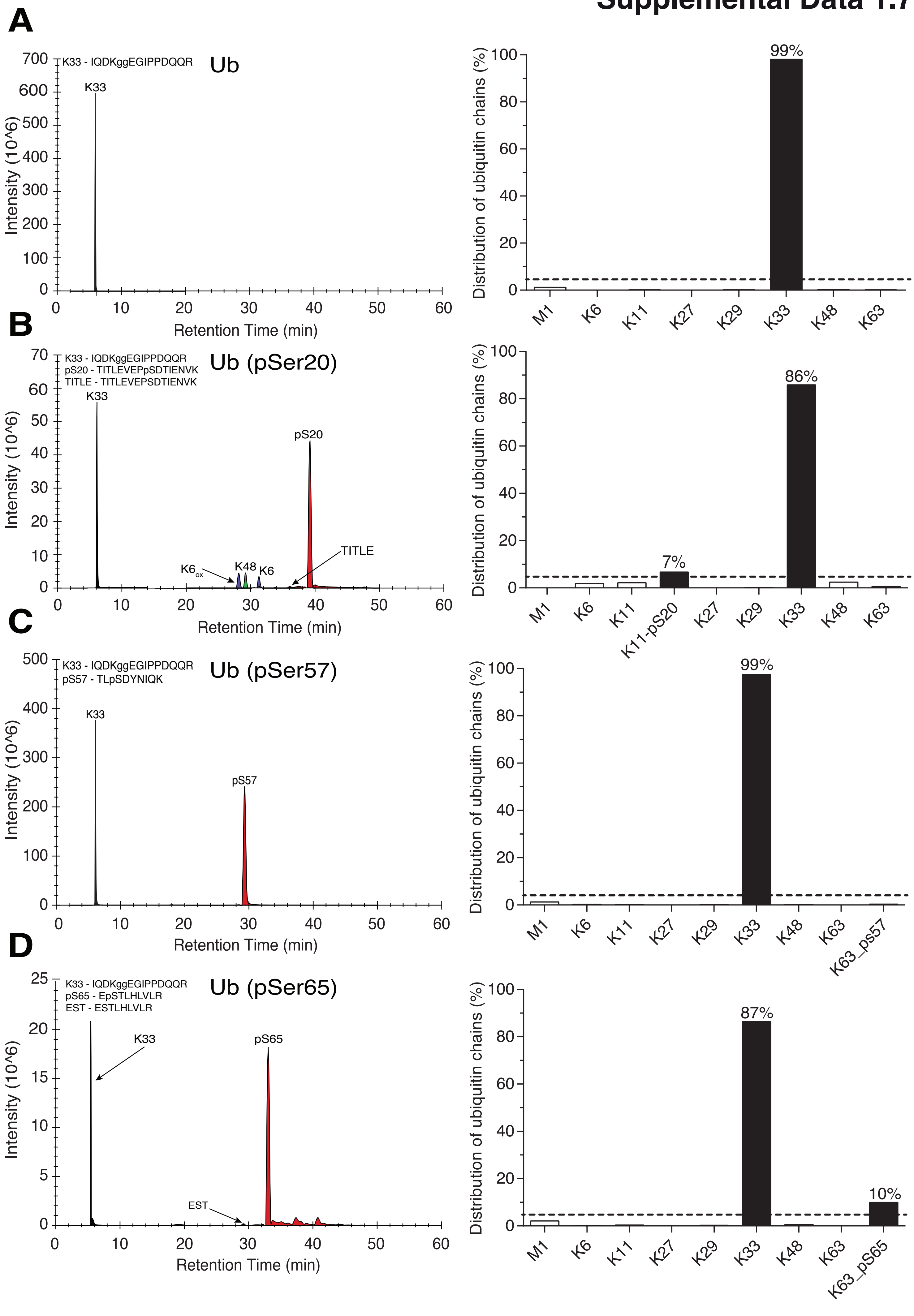

Supplemental Data 1.8

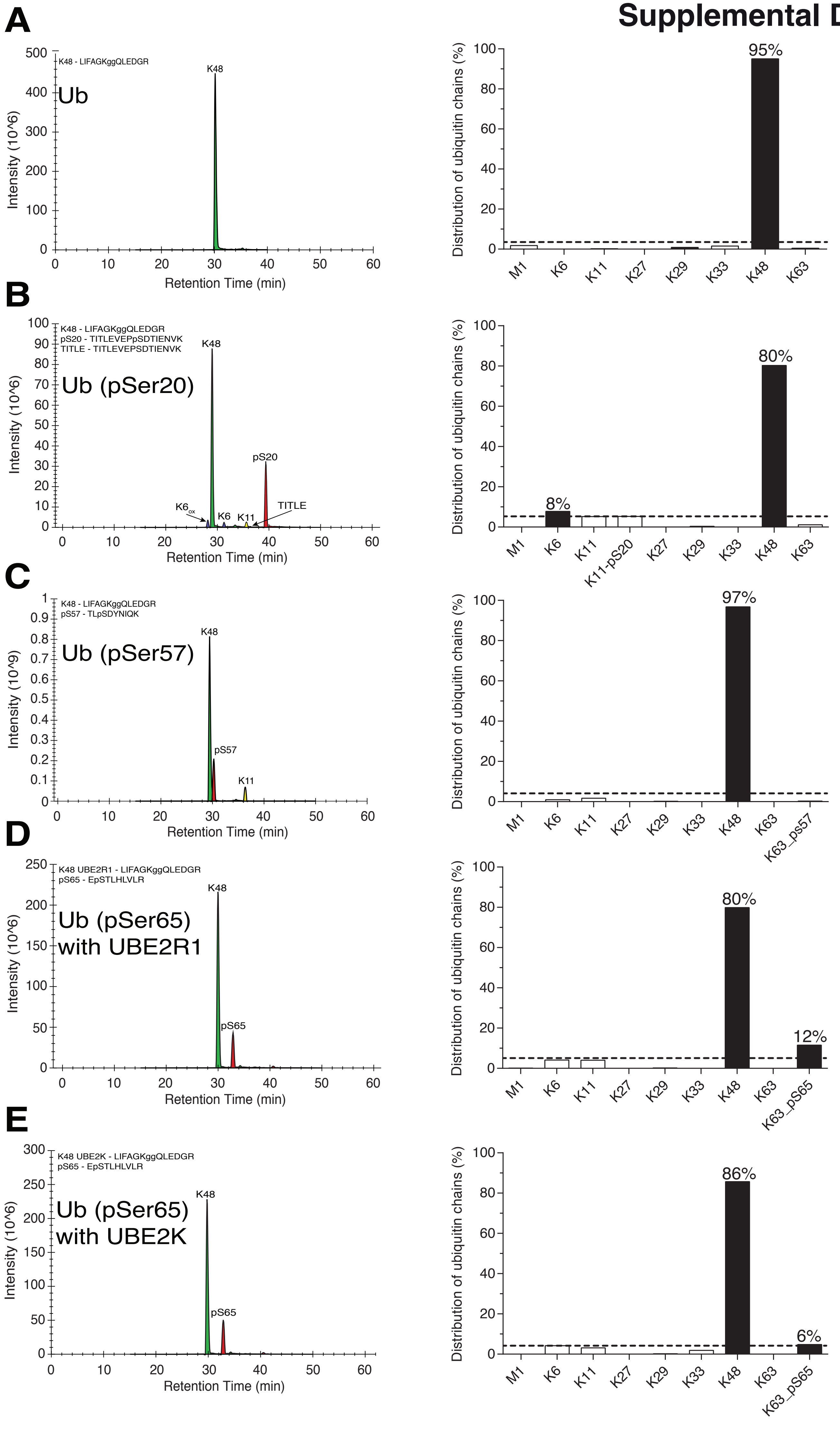

Supplemental Data 1.9

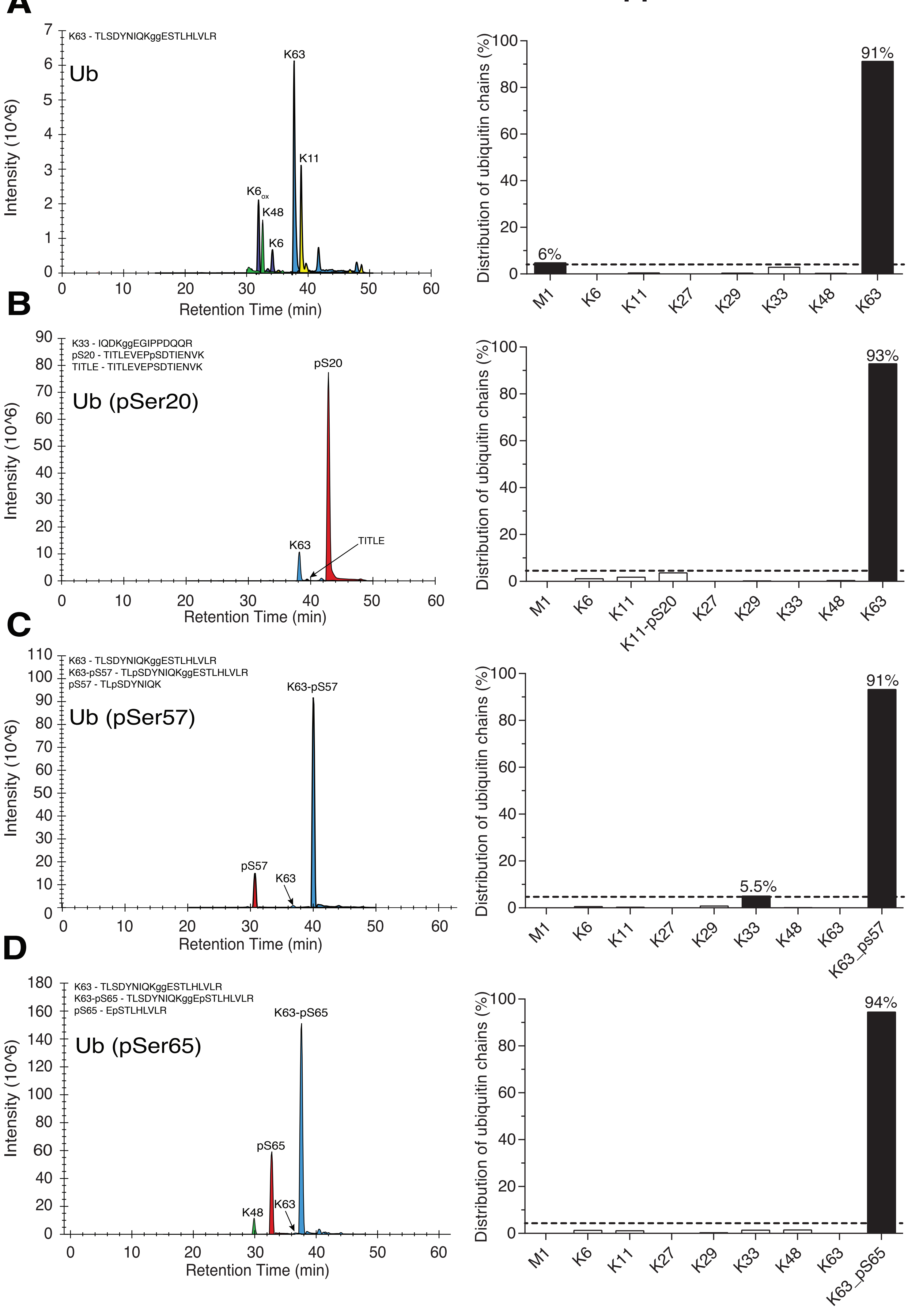

Supplemental Data 1.10

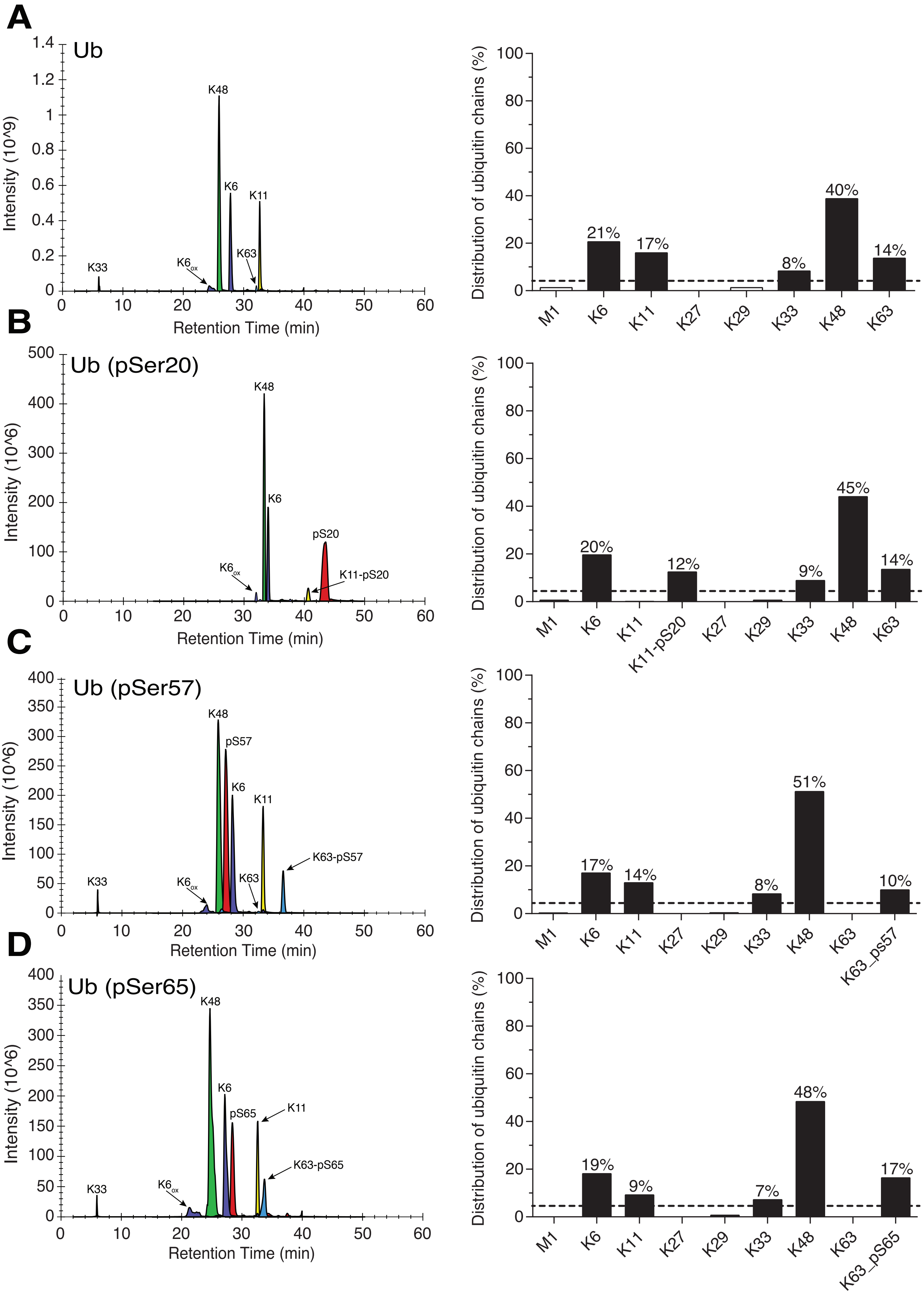

Supplemental Data 1.11

Abbreviation	*Peptide Sequence*	*Parent z*	*Light (m/z)*	*Heavy (m/z)*
M1(ox)	GGM$_{ox}$QIFVK	2	448.2389	451.2458
K6	MQIFVK$_{GG}$TLTGK	2	690.3894	693.898
K6	MQIFVK$_{GG}$TLTGK	3	460.5954	462.9344
K6(ox)	M$_{ox}$QIFVK$_{GG}$TLTGK	2	698.3869	701.8954
K6(ox)	M$_{ox}$QIFVK$_{GG}$TLTGK	3	465.927	468.2661
K11	TLTGK$_{GG}$TITLEVEPSDTIENVK	2	1201.6367	1204.6436
K11	TLTGK$_{GG}$TITLEVEPSDTIENVK	3	801.4269	803.4315
K27	TITLEVEPSDTIENVK$_{GG}$AK	2	1051.6698	1054.6478
K27	TITLEVEPSDTIENVK$_{GG}$AK	3	701.4489	703.4343
K29	AK$_{GG}$IQDK	1	816.4574	822.4775
K29	AK$_{GG}$IQDK	2	408.7323	411.7424
K33	IQDK$_{GG}$EGIPPDQQR	3	546.6129	548.6175
K48	LIFAGK$_{GG}$QLEDGR	2	730.8964	734.405
K48	LIFAGK$_{GG}$QLEDGR	3	487.6001	489.9391
K63	TLSDYNIQK$_{GG}$ESTLHLVLR	3	748.7376	751.0767
K11_pS20	TLTGK$_{GG}$TITLEVEPpSDTIENVK	2	1241.6198	1244.6267
K11_pS20	TLTGK$_{GG}$TITLEVEPpSDTIENVK	3	828.0823	830.0869
K27_pS20	TITLEVEPpSDTIENVK$_{GG}$AK	2	1091.038	1094.0449
K27_pS20	TITLEVEPpSDTIENVK$_{GG}$AK	3	727.6944	729.699
TITLE	TITLEVEPSDTIENVK	2	894.4673	897.4742
TITLE	TITLEVEPSDTIENVK	3	596.6473	598.6519
TITLE_pS20	TITLEVEPpSDTIENVK	2	934.4505	937.4574
TITLE_pS20	TITLEVEPpSDTIENVK	3	623.3027	625.3073
K63_pS57	TLpSDYNIQK$_{GG}$ESTLHLVLR	3	775.3931	777.7321
K63_pS57	TLpSDYNIQK$_{GG}$ESTLHLVLR	4	581.7966	583.5509
TL_pS57-EST	TLpSDYNIQKESTLHLVLR	2	1105.5645	1109.0731
TL_pS57-EST	TLpSDYNIQKESTLHLVLR	3	737.3787	739.7178
TLS-EST	TLSDYNIQKESTLHLVLR	2	1065.5813	1070.5854
TLS-EST	TLSDYNIQKESTLHLVLR	3	710.7233	714.0594
TL_pS57	TLpSDYNIQK	1	1161.5187	1168.5359
TL_pS57	TLpSDYNIQK	2	581.263	584.7716
TLS	TLSDYNIQK	1	1081.5524	1088.5696
TLS	TLSDYNIQK	2	541.2798	544.7884
K63_pS65	TLSDYNIQK$_{GG}$EpSTLHLVLR	3	775.3931	777.7321
K63_pS65	TLSDYNIQK$_{GG}$EpSTLHLVLR	4	581.7966	5835509
TLS-EpS65	TLSDYNIQKEpSTLHLVLR	2	1105.5645	1109.0731
TLS-EpS65	TLSDYNIQKEpSTLHLVLR	3	737.3787	739.7178
TLS-EST	TLSDYNIQKESTLHLVLR	2	1065.5813	1070.5854
TLS-EST	TLSDYNIQKESTLHLVLR	3	710.7233	714.0594
EST_pS65	EpSTLHLVLR	2	574.2972	577.8058
EST_pS65	EpSTLHLVLR	3	383.2006	385.5396
EST	ESTLHLVLR	2	534.314	539.3182
EST	ESTLHLVLR	3	356.5451	359.8812

3. Supplemental Data 1

Supplemental Data 1, related to Figure 3 and 5. Targeted mass spectrometry data and peptide sequences.

Supplemental Data 1.1 related to Figure 3 and 5. Targeted mass spectrometry using AQUA peptides on purified trimers.
M1 (assembled with Ube1, UBE2L3 and HOIP) on either Ub **(A)**, Ub (pSer20) **(B)**, Ub (pSer57) **(C)** or Ub (pSer65) **(D).**

Supplemental Data 1.2 related to Figure 3 and 5. Targeted mass spectrometry using AQUA peptides on purified trimers.
K6 chains (assembled with Ube1, UBE2L3, NleL) on either UbK48R **(A)**, UbK48R (pSer20) **(B)**, UbK48R (pSer57) **(C)** or UbK48R (pSer65) **(D)**.

Supplemental Data 1.3 related to Figure 3 and 5. Targeted mass spectrometry using AQUA peptides on total reactions.
Reactions with Ube1, UBE2L3 and NleL with either Ub **(A)**, Ub (pSer20) **(B)**, Ub (pSer57) **(C)** or Ub (pSer65) **(D).**

Supplemental Data 1.4 related to Figure 3 and 5. Targeted mass spectrometry using AQUA peptides on purified trimers.
K11 chains (assembled with Ube1, UBE2S-UBP and AMSH) on either Ub **(A)**, Ub (pSer20) **(B)**, Ub (pSer57) **(C)** or Ub (pSer65) **(D).**

Supplemental Data 1.5 related to Figure 3 and 5. Targeted mass spectrometry using AQUA peptides on purified trimers.
K29 chains (assembled with Ube1, UBE2D3, UBE3C and vOTU) on either Ub **(A)**, Ub (pSer57) **(B)** or Ub (pSer65) **(C)**.

Supplemental Data 1.6 related to Figure 3 and 5. Targeted mass spectrometry using AQUA peptides on total reactions.
Reactions with Ube1, UBE2D3 and UBE3C with either Ub **(A)**, Ub (pSer20) **(B)**, Ub (pSer57) **(C)** or Ub (pSer65) **(D).**

Supplemental Data 1.7 related to Figure 3 and 5. Targeted mass spectrometry using AQUA peptides on purified trimers.
K33 chains (assembled with Ube1, UBE2D1, AREL1, OTUB1 and Cezanne-EK) on either Ub **(A)**, Ub (pSer20) **(B)**, Ub (pSer57) **(C)** or Ub (pSer65) **(D).**

Supplemental Data 1.8 related to Figure 3 and 5. Targeted mass spectrometry using AQUA peptides on purified trimers.
K48 chains (assembled with Ube1, UBE2R1 or UBE2K) on either Ub **(A)**, Ub (pSer20) **(B)**, Ub (pSer57) **(C)**, Ub (pSer65) with UBE2R1 **(D)** or with UBE2K **(E).**

Supplemental Data 1.9 related to Figure 3 and 5. Targeted mass spectrometry using AQUA peptides on purified trimers.

K63 chains (assembled with Ube1, UBE2N and UBE2V1) on either Ub **(A)**, Ub (pSer20) **(B)**, Ub (pSer57) **(C)** or Ub (pSer65) **(D)**.

Supplemental Data 1.10 related to Figure 3 and 5. Targeted mass spectrometry using AQUA peptides on total reactions.
Reaction with Ube1, UBE2L3 and HHARI with either Ub **(A)**, Ub (pSer20) **(B)**, Ub (pSer57) **(C)** or Ub (pSer65) **(D)**.

Supplemental Data 1.11 related to Figure 3 and 5. List of isotopically labelled peptides used to monitor polyUb and phosphorylated polyUb linkages.
Signature peptides representing isopeptide-linked polyUb linkages are denoted by a subscript "GG" adjacent to the modified lysine. Signature peptides representing phosphorylated peptides are denotes by a "p" adjacent to modified serine. Isotopically labelled amino acids are denoted in blue and the oxidation state of Met-containing peptides is denoted by "ox". For each peptide, the optimal precursor ions to the HCD collision energy of 30% were selected for the PRM analysis.
