# Supplementary material for: Synthesis of Isomeric Phosphoubiquitin Chains Reveals that Phosphorylation Controls Deubiquitinase Activity and Specificity
Source: Cell Rep. 2016 Jul 14;16(4):1180–93. doi: 10.1016/j.celrep.2016.06.064 (PMC4967478; doi:10.1016/j.celrep.2016.06.064)
Supplement: Document S2. Article plus Supplemental Information [file mmc4.pdf]

Cell Reports

Resource

Synthesis of Isomeric Phosphoubiquitin Chains Reveals that Phosphorylation Controls Deubiquitinase Activity and Specificity

Graphical Abstract

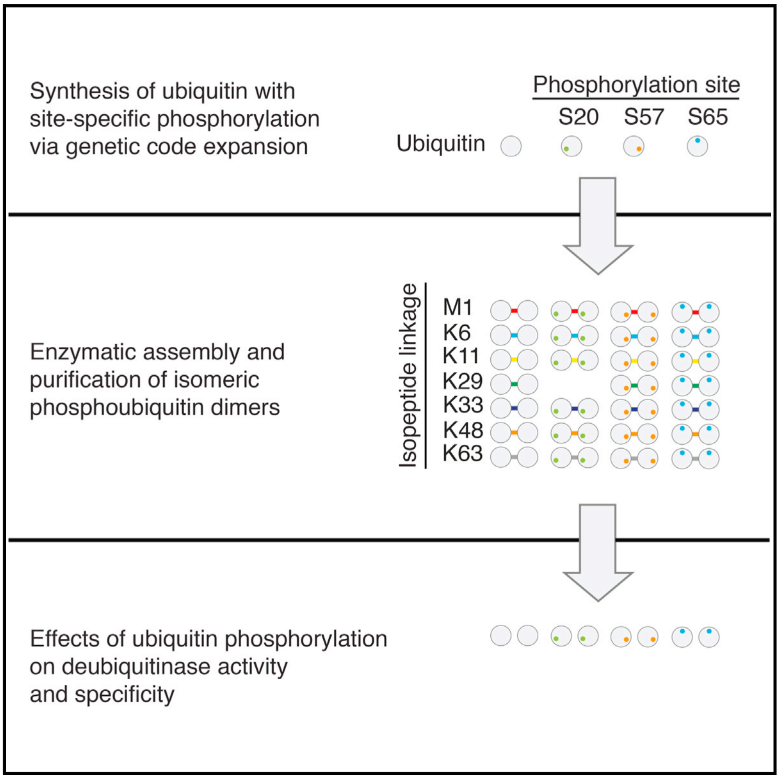

Authors

Nicolas Huguenin-Dezot, Virginia De Cesare, Julien Peltier, ..., Yogesh Kulathu, Matthias Trost, Jason W. Chin

Correspondence

m.trost@dundee.ac.uk (M.T.), chin@mrc-lmb.cam.ac.uk (J.W.C.)

In Brief

Huguenin-Dezot et al. combine genetic code expansion and enzymatic assembly to synthesize 20 isomeric phosphoubiquitin chains with distinct Ser phosphorylation and/or isopeptide linkage sites. They discover that ubiquitin phosphorylation can control E3 ligase specificity and deubiquitinase specificity.

Highlights

- Milligram quantities of ubiquitin phosphorylated at Ser 20, 57, or 65 are purified
- Twenty isomeric phosphoubiquitin dimers are assembled and purified
- UBE3C chain synthesis specificity is controlled by Ser 20 ubiquitin phosphorylation
- Phosphorylation of ubiquitin controls deubiquitinase activity and linkage specificity

Accession Numbers

5K9P

Huguenin-Dezot et al., 2016, Cell Reports *16*, 1180–1193
July 26, 2016 © 2016 MRC Laboratory of Molecular Biology.
http://dx.doi.org/10.1016/j.celrep.2016.06.064

OPEN ACCESS
CellPress

Cell Reports
Resource

Synthesis of Isomeric Phosphoubiquitin Chains Reveals that Phosphorylation Controls Deubiquitinase Activity and Specificity

Nicolas Huguenin-Dezot,[1] Virginia De Cesare,[2] Julien Peltier,[2] Axel Knebel,[2] Yosua Adi Kristaryianto,[2] Daniel T. Rogerson,[1] Yogesh Kulathu,[2] Matthias Trost,[2,*] and Jason W. Chin[1,*]
[1]Medical Research Council Laboratory of Molecular Biology, Francis Crick Avenue, CB2 OQH Cambridge, UK
[2]Medical Research Council Protein Phosphorylation and Ubiquitylation Unit, University of Dundee, DD1 5EH Dundee, UK
*Correspondence: m.trost@dundee.ac.uk (M.T.), chin@mrc-lmb.cam.ac.uk (J.W.C.)
http://dx.doi.org/10.1016/j.celrep.2016.06.064

SUMMARY

Ubiquitin is post-translationally modified by phosphorylation at several sites, but the consequences of these modifications are largely unknown. Here, we synthesize multi-milligram quantities of ubiquitin phosphorylated at serine 20, serine 57, and serine 65 via genetic code expansion. We use these phosphoubiquitins for the enzymatic assembly of 20 isomeric phosphoubiquitin dimers, with different sites of isopeptide linkage and/or phosphorylation. We discover that phosphorylation of serine 20 on ubiquitin converts UBE3C from a dual-specificity E3 ligase into a ligase that primarily synthesizes K48 chains. We profile the activity of 31 deubiquitinases on the isomeric phosphoubiquitin dimers in 837 reactions, and we discover that phosphorylation at distinct sites in ubiquitin can activate or repress cleavage of a particular linkage by deubiquitinases and that phosphorylation at a single site in ubiquitin can control the specificity of deubiquitinases for distinct ubiquitin linkages.

INTRODUCTION

The post-translational modification of proteins with ubiquitin (Ub) modulates an expanding array of cellular processes (Komander and Rape, 2012). Ub is attached to a target protein through the formation of an isopeptide bond between a lysine in the target protein and the C terminus of the Ub. A cascade of enzymes (E1s, E2s, and E3s) directs protein ubiquitination: Ub is first activated, as a thioester conjugate to a cysteine residue in an E1-activating enzyme, and is then transferred to the active site cysteine of an E2-conjugating enzyme, before it is conjugated to the target protein with the help of an E3 ligase. The E3 may either activate direct transfer of Ub from the E2 (RING and U-box E3 ligases), or it may transiently accept Ub from the E2 to form an E3-Ub intermediate before transfer of Ub to the target amine (RBR and HECT E3 ligases) (Berndsen and Wolberger, 2014). In human cells there are two E1s, ~40 E2s, and >600 E3s (Clague et al., 2015).

The action of the E1, E2, and E3 cascade is counteracted by deubiquitinases (DUBs) that cleave the isopeptide bond between the target protein and Ub. There are five established families of DUBs: Ub-specific proteases (USPs), Ub C-terminal hydrolases (UCHs), ovarian tumor proteases (OTUs), Josephins, and JAMMs (Komander et al., 2009). The interplay between the specificity of the E1, E2, and E3 systems and the specificity of DUBs defines the ubiquitinated proteome, and factors that alter the activity and specificity of these enzymes reconfigure the ubiquitinated proteome and alter biological outcomes.

Many proteins have been identified as targets of ubiquitination. Proteins can be mono-ubiquitinated or modified with Ub polymers, in which Ub is linked via an isopeptide bond between the C terminus of one monomer and a lysine residue (K6, K11, K27, K29, K33, K48, or K63) or via a peptide bond to the N terminus (M1) of another monomer.

Ub linkages have been identified in cells at varying abundances (Kulathu and Komander, 2012). Because of the low abundance of Ub chains (Kaiser et al., 2011) and the challenge of purifying specific Ub linkage isomers from cells, the development of methods for the synthesis of well-defined Ub chains (Faggiano et al., 2016; Kumar et al., 2010; Virdee et al., 2010) has been crucial for characterizing the structural and biochemical properties of Ub chains. The characterization of atypical Ub chains has often preceded an understanding of their physiological significance, and biochemical and structural data for Ub chains of unknown function continue to inform in vivo experiments aimed at addressing physiological significance (Bremm et al., 2010; Hospenthal et al., 2013; Kristariyanto et al., 2015; Michel et al., 2015).

Ub is a target for post-translational modifications, including acetylation (Ohtake et al., 2015) and phosphorylation (Kane et al., 2014; Kazlauskaite et al., 2014; Koyano et al., 2014). Several sites of phosphorylation (Thr7, Thr12, Thr14, Ser20, Ser57, Tyr59, Ser65, and Thr66) have been identified on Ub within mammalian cells (Herhaus and Dikic, 2015).

We recently reported the directed evolution of SepRS/$tRNA_{CUA}$ pairs (Rogerson et al., 2015) that function with a mutant of EF-Tu (Park et al., 2011) for the efficient genetic encoding of

 © 2016 MRC Laboratory of Molecular Biology.
This is an open access article under the CC BY license (http://creativecommons.org/licenses/by/4.0/).

phosphoserine and its non-hydrolyzable analog in response to an amber codon, which can be introduced into a gene of interest at a desired position (Rogerson et al., 2015). Here we use this approach to synthesize phosphoubiquitin isomers bearing phosphorylation at the three serines in Ub that are modified in vivo (serine 20, serine 57, and serine 65).

The physiological significance for serine 65 phosphorylation is the most well-established of any phosphorylation in Ub. Ser65 phosphorylation on Ub is installed by PINK1, which also phosphorylates the E3 ligase Parkin (Kane et al., 2014; Kazlauskaite et al., 2014; Kondapalli et al., 2012; Koyano et al., 2014; Lai et al., 2015; Shiba-Fukushima et al., 2012). Mutations in PINK1 and Parkin are the most common cause of autosomal recessive Parkinson's disease (Corti et al., 2011; Valente et al., 2004). It appears that phosphorylation of both Parkin and Ub is required to activate Parkin and to ubiquitinate and clear damaged mitochondria from cells (Kumar et al., 2015; Lazarou et al., 2015). Ser65 phosphorylation is detected as <1.5% of global Ub in human cells, but the fraction of phosphorylated Ub at the mitochondria can reach 10%–20%. These observations further demonstrate that low global levels of phosphoubiquitin species are consistent with physiologically relevant function.

Ub phosphorylated at Ser65 may be prepared by enzymatic phosphorylation with PINK1 (Wauer et al., 2015). However, for the other serine phosphorylation sites on Ub, the kinases that install the phosphorylation are unknown, and the molecular consequences of Ub phosphorylation remain uncharacterized. Serine 57 is the major (most abundant) phosphorylation site on Ub (Swaney et al., 2015), and it has been found repeatedly in independent studies in yeast and mammalian cells (Bennetzen et al., 2010; Bian et al., 2014; Malik et al., 2009; Phanstiel et al., 2011; Sharma et al., 2014; Swaney et al., 2015; Villén et al., 2007; Zhou et al., 2013). Recently, serine 57 phosphorylation was shown to be globally upregulated in response to oxidative stress (Swaney et al., 2015). Serine 20 phosphorylation has been detected repeatedly in human, mouse, and rat (Choudhary et al., 2009; Lundby et al., 2012; Manes et al., 2011).

Here we used genetic code expansion to express and purify multi-milligram quantities of Ub bearing homogeneous phosphorylation at Ser20 (Ub [pSer20]), Ser57 (Ub [pSer57]), and Ser65 (Ub [pSer65]) from *E. coli* and to solve the structure of Ub (pSer20). We determined the consequences of each phosphorylation for E1 activation and E2 conjugation by 18 E2s. We assembled and purified 20 phosphorylated Ub dimers, with distinct sites of peptide linkage (M1, K6, K11, K29, K33, K48, and K63) and phosphorylation (Ser20, Ser57, and Ser65), using the E2/E3 combinations reported for the synthesis of unmodified Ub chains. In the process of assembling and characterizing phosphoubiquitin chains, we discovered that phosphorylation of serine 20 on Ub converts UBE3C from a dual-specificity E3 ligase into a ligase that primarily synthesizes K48 chains. DUB assays with phosphorylated Ub dimers revealed that (1) phosphorylation at distinct sites on Ub can control DUB-mediated cleavage of a given Ub linkage, and that (2) phosphorylation at each site in Ub can control the specificity with which DUBs cleave different Ub linkages.

RESULTS

Production of Ub (pSer20), Ub (pSer57), and Ub (pSer65)

We expressed each phosphorylated Ub variant from the corresponding Ub (Ser XX TAG)-His6 gene, where XX defines the site of serine phosphorylation in the protein, TAG is the amber codon, and His6 encodes the hexahistidine tag. Phosphoserine was directed into the protein using an evolved SepRS/$tRNA_{CUA}$ pair, an approach that we previously demonstrated allows the production of homogeneous and site-specifically phosphorylated Ub and other phosphoproteins (Rogerson et al., 2015). Ub (pSer20), Ub (pSer57), and Ub (pSer65) were purified with yields of 1.5, 0.5, and 24 mg/l, respectively (Figure 1A). We also expressed and purified the corresponding K48R mutants of Ub required for the assembly of K6-linked Ub chains (Hospenthal et al., 2013). Electrospray ionization mass spectrometry (ESI-MS) confirmed the phosphorylation of each Ub (Figure 1B), and targeted MS confirmed the site of phosphorylation (Kirkpatrick et al., 2006) (Data S1). While a previous report also claimed to have made phosphoubiquitin species using an unevolved SepRS/$tRNA_{CUA}$ pair (Ordureau et al., 2015; Park et al., 2011), no characterization of phosphoserine incorporation was reported. Moreover, we previously demonstrated that the unevolved synthetase/tRNA pair is 20 times less efficient than the evolved pair we used here (Rogerson et al., 2015), and others previously demonstrated that the unevolved SepRS/$tRNA_{CUA}$ pair incorporates natural amino acids into proteins in response to the amber codon, leading to heterogeneous protein products (Heinemann et al., 2012), which make interpreting biochemical experiments challenging.

We obtained crystals for Ub (pSer20), which diffracted to 1.55 Å (Table S1) and showed clear density for the phosphate group (Figure 1C). This underscores the quantity and purity of proteins obtained using the evolved SepRS/$tRNA_{CUA}$ pair (Rogerson et al., 2015). The fold of the phosphorylated protein is comparable to that of unphosphorylated Ub (root-mean-square deviation [RMSD] to 1UBQ [Vijay-Kumar et al., 1987] residues 1–72 is 0.8 Å; Figure 1D), though the charge distribution on one face of the molecule is perturbed (Figure 1E).

Phosphorylation at Ser20, Ser57, or Ser65 Does Not Affect the Loading of E2s with Ub

To test whether phosphorylation at serine in position 20, 57, or 65 influenced the first two steps in the ubiquitination cascade, E2-charging reactions containing an E1 (Ube1), ATP, and 18 different E2s were performed (Figures 2A–2R). We did not observe differences in charging efficiency among Ub (pSer20), Ub (pSer57), Ub (pSer65), and unmodified Ub. We conclude that phosphorylation at Ser20, Ser57, or Ser65 does not affect the first two steps of the ubiquitination cascade with the E2s tested. Our results define the effects of Ser20 and Ser57 phosphorylation of Ub on E2 charging, and they are consistent with previous observations, using a subset of the E2s we have investigated (Wauer et al., 2015), for Ser65 phosphorylation.

Synthesis of Isomeric Phosphoubiquitin Chains

Next we asked whether Ub (pSer20), Ub (pSer57), and Ub (pSer65) chains linked through M1, K6, K11, K29, K33, K48, or

OPEN ACCESS
CellPress

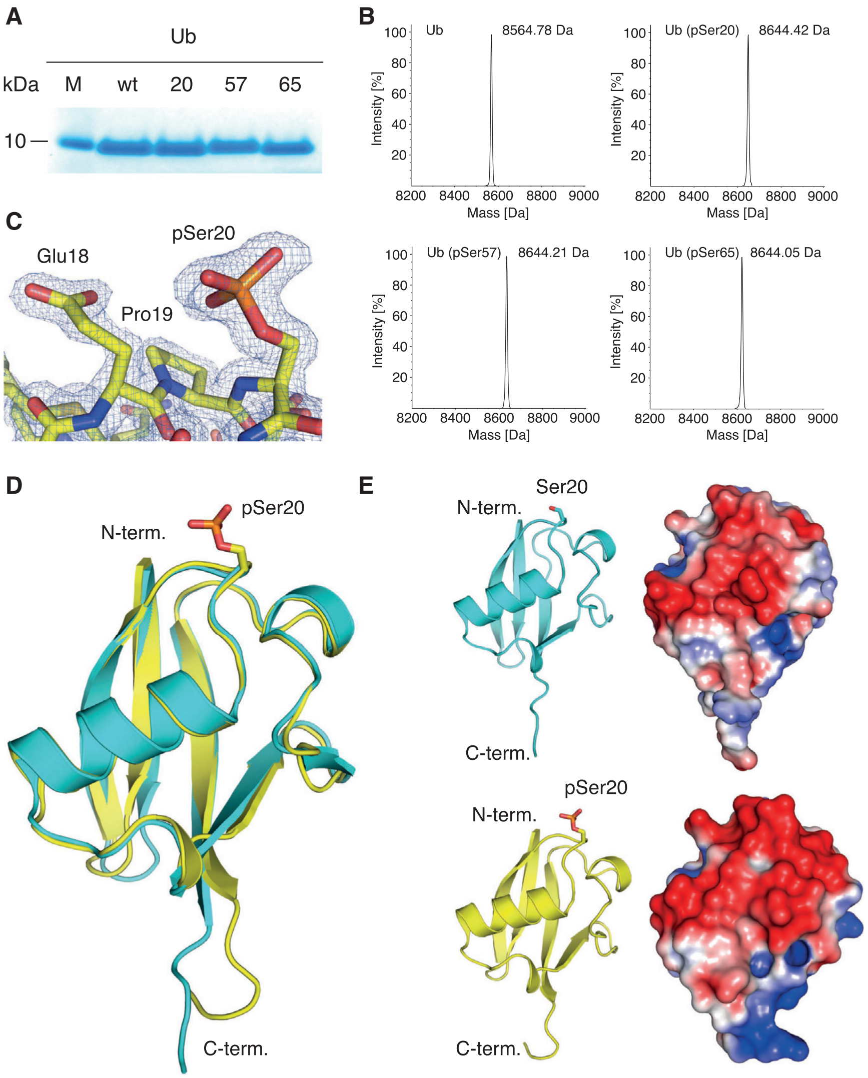

Figure 1. Production and Characterization of Ub (pSer20), Ub (pSer57), and Ub (pSer65)

(A) Coomassie staining of equal amounts of unmodified Ub and phosphorylated Ub. 20, 57, and 65 designate the phosphorylated serine residue on Ub.

(B) ESI-MS demonstrates the quantitative incorporation of pSer into Ub (Ub: expected 8,564.8 Da, observed 8,564.78 Da; Ub (pSer20): expected 8,644.78 Da, observed 8,644.42 Da; Ub (pSer57): expected 8,644.78 Da, observed 8,644.21 Da; and Ub (pSer65): expected 8,644.78 Da, observed, 8,644.05 Da).

(C) 2FoFc map around the phosphate of Ub (pSer20) contoured at 1.3 sigma is shown.

(D) Alignment of the structure of Ub (pSer20) (yellow) and a Ub reference structure (PDB: 1UBQ; Vijay-Kumar et al., 1987) (light blue), RMSD for residues 1–72 = 0.8 Å (calculated with DALI [Hasegawa and Holm, 2009]). The phosphate atom is displayed in orange and oxygen atoms are in red.

(E) Electrostatic surface potential changes on the surface of Ub, unmodified (top) and pSer20 (bottom) in similar orientation. Potentials were calculated with the APBS plug-in within PyMOL, using established phosphoserine parameters (Steinbrecher et al., 2012).

See also Table S1.

K63 on Ub can be formed using combinations of enzymes that are known to assemble these chains with unmodified Ub (Faggiano et al., 2016). We followed the extent of Ub chain formation as a function of time by SDS-PAGE (Figure 3) and the composition of the chain formed by targeted MS, with isotopically labeled, absolute quantitation (AQUA) peptide standards (Kirkpatrick et al., 2006) (henceforth referred to as targeted MS) (Data S1).

For the enzymes that assemble M1-linked linear Ub chains (Ube1, UBE2L3, and HOIP), the extent of chain formation was not attenuated with Ub (pSer20) and Ub (pSer57) (Figure 3A). However, we observed a decrease in chain formation for Ub (pSer65), consistent with previous observations (Wauer et al., 2015). Phosphorylation at position 20 of Ub increased the fraction of K11 linkages formed (from undetectable levels with unmodified Ub to 9% with Ub [pSer20]), while phosphorylation at position 65 increased the fraction of K33 linkages formed to 10% with Ub (pSer65) (Data S1).

For the enzymes that assemble K6 chains (Ube1, UBE2L3, and NleL), using K48R mutants of Ub to avoid forming K48 chains, we observed no substantial differences in the extent of Ub chain formation with the K48R Ub species tested (Figure 3B). However, K11 linkages were formed with Ub (K48R, pSer20) (7%) and K63 linkages were formed with Ub (K48R, pSer65) (10%) (Data S1).

K11- and K63-linked Ub chains can be formed by Ube1 and UBE2S-UBP. The addition of AMSH removes K63 chains leaving pure K11-linked Ub. We observed no substantial effect on the extent of chain formation with Ub (pSer20), Ub (pSer57), or Ub (pSer65) (Figure 3C), and Ub (pSer20) and Ub (pSer57) predominantly formed K11 chains (Data S1). However, Ub (pSer65) led to 33% K11 linkages and 62% K63 linkages (Data S1). These observations are consistent with our DUB assays (Figure 5), which revealed that AMSH does not cleave K63-linked Ub (pSer65) dimers (Figure 5G). We conclude that Ube1 and UBE2S-UBP form a mixture of K11- and K63-linked Ub (pSer65), which is not further modified by AMSH.

A mixture of K29- and K48-linked chains can be assembled by Ube1, UBE2D3, and UBE3C. Addition of the DUB vOTU cleaves K48 chains in this mixture, leaving pure K29 chains (Kristariyanto et al., 2015). We observed a striking decrease in the extent of chain formation for Ub (pSer20) when incubated with Ube1, UBE2D3, UBE3C, and vOTU (Figure 3D). Further investigation revealed that UBE3C forms Ub (pSer20) chains in the absence of vOTU (Figures 4A and S1), and these chains contain K48 linkages, but not K29 linkages (Figures 4A–4C). Our results reveal that Ub that is phosphorylated on Ser20 is a substrate for the

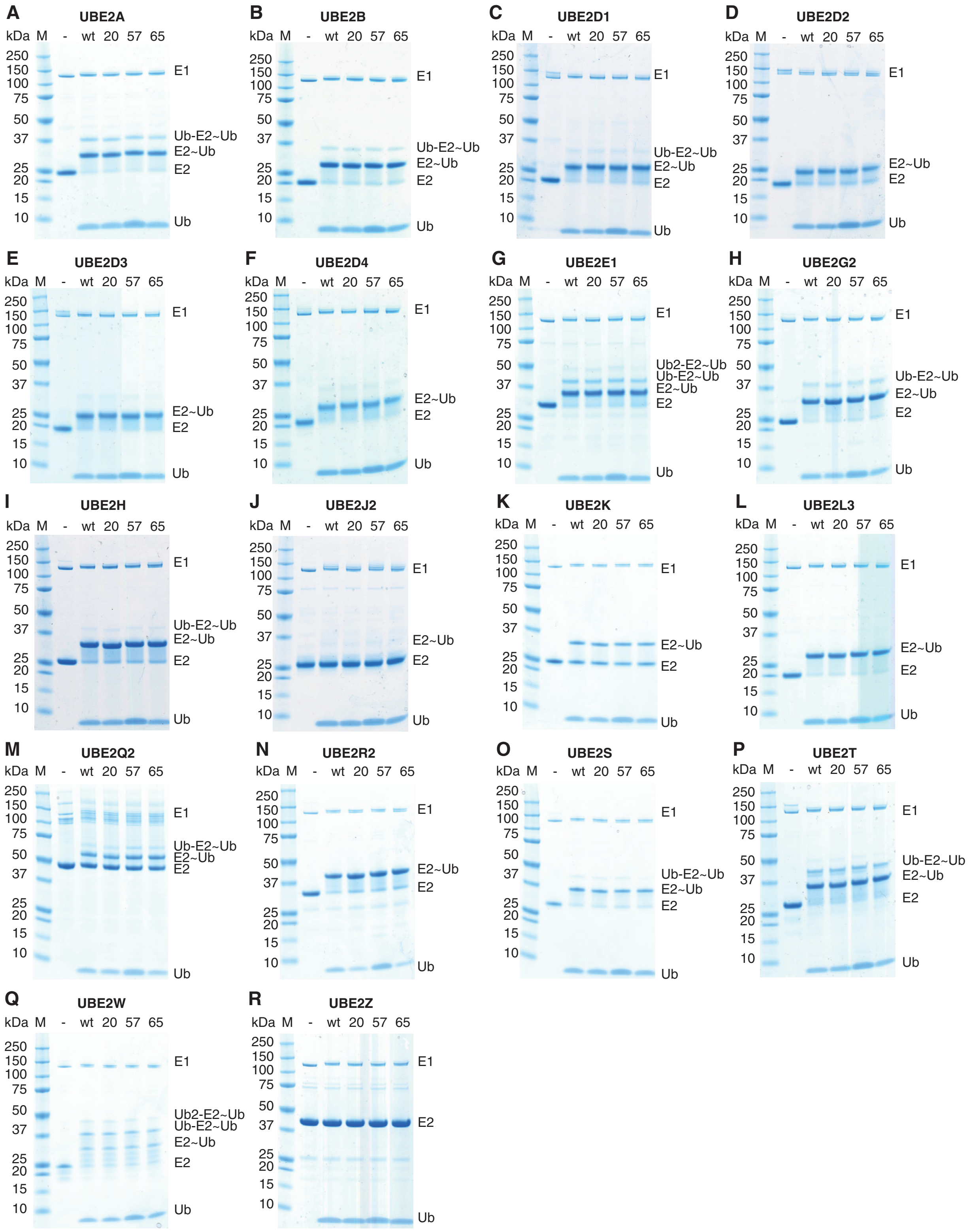

Figure 2. E1 Activation and E2 Charging Are Not Affected by Serine Phosphorylation in Ub

(A–R) Commassie staining of E2-charging reactions containing an E1 (Ube1), ATP, 18 different E2s, and Ub, Ub (pSer20), Ub (pSer57), or Ub (pSer65) after 1 hr (A–J, L, P, and R) or 5 min (K, M–O, and Q) incubation at 30°C. Ub, Ubiquitin; E2∼Ub, Thioester-linked E2-Ub pair; Ub-E2∼Ub, Thioester-linked E2-Ub pair with one covalently E2 attached Ub; 20, 57, and 65 designate the phosphorylated serine residue on Ub. See also Figure S2 and Table S2.

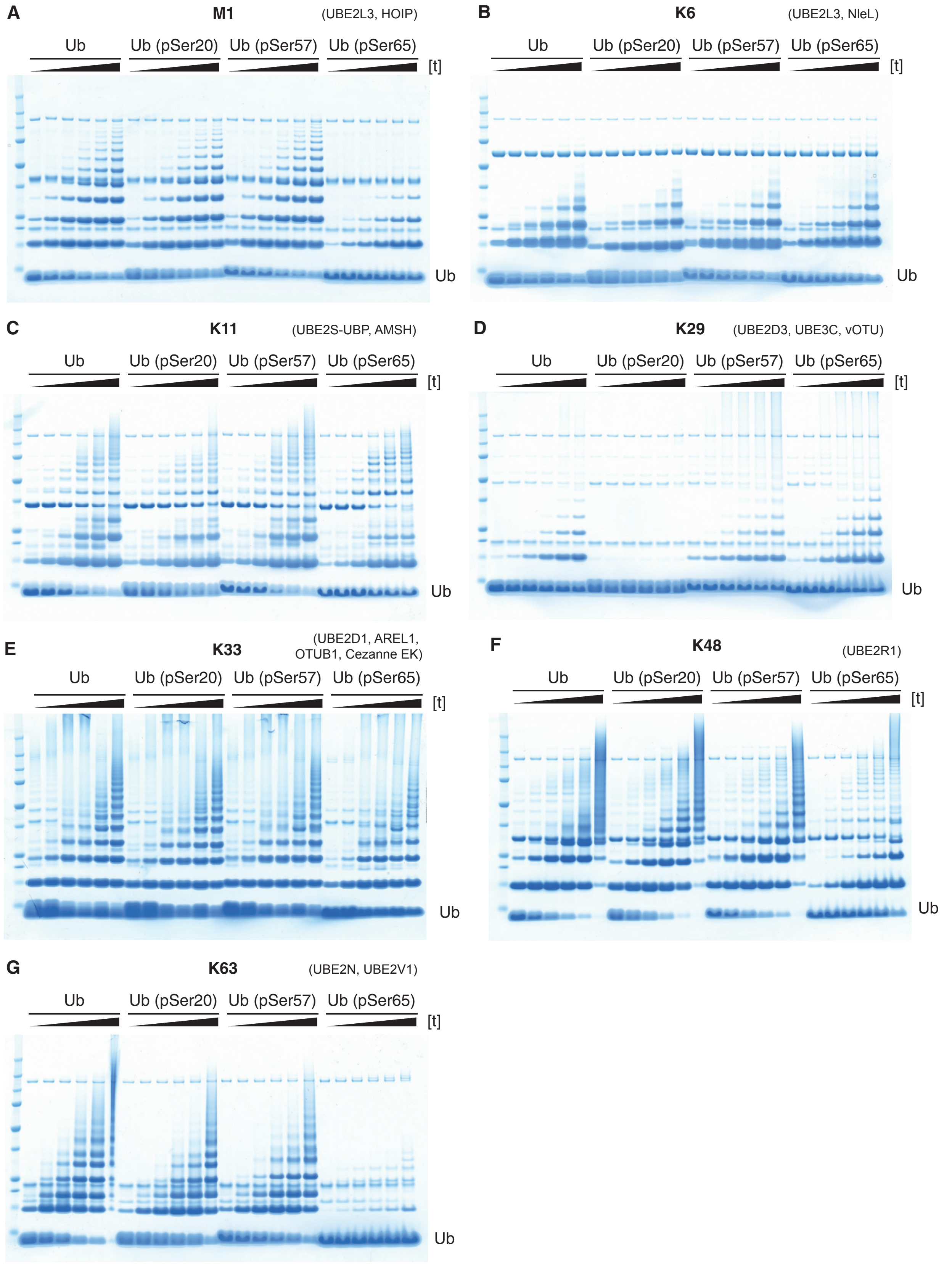

(legend on next page)

K48 ligase activity of UBEC3, but it is not a substrate for the K29 ligase activity of UBE3C. We conclude that Ser20 phosphorylation on Ub changes UBE3C from a dual-specificity ligase that makes K48- and K29-linked chains with unmodified Ub to a ligase that makes predominantly Ser20 phosphorylated K48-linked chains with Ser20 phosphorylated Ub (Figure 6B). In contrast, phosphorylation at Ser57 or Ser65 of Ub has more modest effects on UBE3C ligase activity (Data S1).

A mixture of K33-, K11-, and K48-linked chains can be assembled with Ube1, UBE2D1, and AREL1. To create pure K33 chains, the DUBs Cezanne-EK and OTUB1 are added to hydrolyze K11 and K48 chains, respectively, and leave pure K33 chains (Michel et al., 2015). The phosphorylation sites investigated did not substantially influence chain formation with these enzymes (Figure 3E). Moreover, most of the Ub linkages formed were at K33 for Ub (pSer20), Ub (pSer57), or Ub (pSer65) and Ub (Data S1). However, consistent with our observation that K11-linked Ub (pSer20) is a worse substrate for Cezanne than Ub (Figure 5C), we observed 7% of K11 linkages for Ub (pSer20).

We used an E1/E2 combination (Ube1 and UBE2R1) that assembles K48 chains with Ub for chain assembly with each of the phosphorylated Ubs. We found that chain assembly was attenuated for Ub (pSer65) (Figures 3F and S2). Phosphorylation of Ser65 in Ub led to a decrease in the fraction of K48 chains formed and an increase in the formation of K63 linkages (from undetectable levels for Ub to 12% of linkages for Ub [pSer65]; Data S1), in qualitative agreement with previous work (Wauer et al., 2015). We found that Ser20 phosphorylation also decreased the fraction of K48 linkages and led to an increase in K6 linkages with Ube1 and UBE2R1 (Data S1).

We used an E1/E2 combination (Ube1, UBE2N, and UBE2V1) that assembles K63 chains with unmodified Ub for chain assembly with each of the phosphorylated Ubs. We observed a modest attenuation of chain assembly for Ub (pSer20) and Ub (pSer57) and a more dramatic attenuation of chain assembly for Ub (pSer65) (Figure 3G). However, phosphorylation at the sites tested did not affect the linkage composition (Data S1). We conclude that phosphorylation of Ub affects the kinetics of K63 chain formation with these enzymes but does not substantively affect the composition of the chains formed.

Phosphorylation of Ub at Ser20, Ser57, and Ser65 Controls DUB Specificity

Next we addressed the consequences of Ub phosphorylation for DUB activity and specificity. We purified dimers of Ub, dimers of Ub (pSer20), dimers of Ub (pSer57), and dimers of Ub (pSer65) from each linkage assembly reaction (Figure S3), yielding 27 dimeric substrates for DUB profiling. To profile activity of DUBs on phosphoubiquitin dimers, we adapted a MALDI-TOF assay that previously was used to profile DUB activity, specificity, and inhibition on unmodified Ub dimers (Ritorto et al., 2014). We followed the extent of phosphorylated diubiquitin cleavage by the appearance of the monomeric phosphoubiquitin species in MALDI-TOF, using quantitatively phosphorylated ^{15}N-labeled Ub as an internal standard.

We tested the cleavage of each of the 27 purified Ub dimers with 31 DUBs in 837 Ub dimer/DUB combinations (Figures 5 and S4). The DUBs profiled included members of the USP, OTU, and JAMM families that can be purified and assayed in vitro, and they cover about a third of the DUBs encoded in the human genome. A potential caveat in interpreting some of the DUB data comes from the observation that some of the reactions used to assemble the Ub dimers led to small amounts of other linkages (Figure S4; Data S1). However, because we also have data on the cleavage of the other linkages by the same DUB and most Ub phosphorylations inhibit DUB cleavage, the contaminating linkages were not cleaved in most cases. Therefore, it was still possible to clearly interpret the DUB specificity data.

DUBs did not cleave the unphosphorylated Ub dimer for 88 of the Ub dimer linkage/DUB combinations tested. Phosphorylation of Ub at Ser20, Ser57, or Ser65 did not activate DUB-mediated cleavage of the Ub dimers that were not cleaved when Ub was unmodified (Figure S4). For the remaining 479 Ub dimer/DUB combinations, we compared the activity of the DUB on each phosphorylated Ub dimer to the activity of the DUB on the unmodified Ub dimer of the same linkage (Figure 5). Most DUBs tested were less able to cleave the phosphorylated Ub dimers. Ser65 phosphorylation led to the greatest reduction of Ub dimer cleavage by most of the DUBs tested (Figure 5).

Our data define the role of Ub phosphorylation at Ser20, Ser57, and Ser65 in regulating the DUB-mediated cleavage of each Ub linkage (Figures 5 and 6). CYLD had comparable activity on Ub (pSer65) linear Ub dimers and unmodified linear dimers of Ub, but it was much less active on linear dimers of Ub (pSer20) or Ub (pSer57) (Figures 5A and 6C).

USP8 cleaved K6-linked dimers of Ub (pSer20) and Ub (pSer57) more efficiently than K6-linked dimers of Ub, and phosphorylation of Ub at residue 65 substantially reduced K6 dimer cleavage by USP8 (Figures 5B and 6D). In contrast, the cleavage

Figure 3. The Synthesis of Ub Chains Is Differentially Affected by Phosphorylation at Different Sites in Ub

Chain assembly time course reactions are shown. All reactions contained Ube1 and the E2s, E3s, and DUBs indicated. The protein ladder is Precision Plus All Blue Prestained Protein Standards (BioRad).

(A) M1 chain assembly. Time (left to right): 1, 5, 10, 20, 30, and 40 min.

(B) K6 chain assembly. UbK48R, UbK48R (pSer20), UbK48R(pSer57), or UbK48R(pSer65) were used for these reactions. Time (left to right): 1, 5, 10, 15, 30, and 60 min.

(C) K11 chain assembly. The DUB AMSH was added after the 6 hr time point. Time (left to right): 5, 15, 40, 200, and 360 min and overnight.

(D) K29 chain assembly. Time (left to right): 5, 15, 60, 180, and 360 min and overnight.

(E) K33 chain assembly. The DUBs OTUB1 and Cezanne EK were added to the reaction after the 6 hr time point. Time (left to right): 0.5, 1, 4, 6, and 8 hr and overnight.

(F) K48 chains were assembled with Ube1 and UBE2R1. Time (left to right): 15, 45, 120, 240, and 360 min and overnight.

(G) K63 chain assembly. Time (left to right): 15 min; 1, 2, 4, and 6 hr; and overnight.

See also Figure S3, Table S2, and Data S1.

OPEN
ACCESS
CellPress

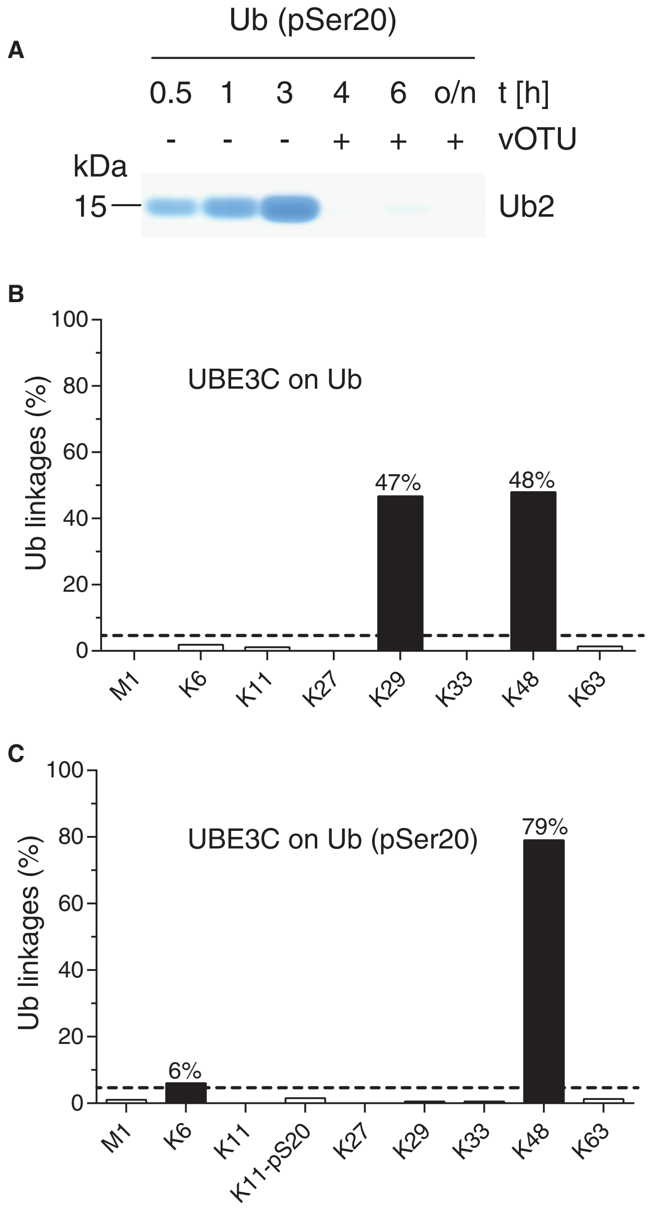

Figure 4. UBE3C Is Converted from a Dual-Specificity Ligase to a K48-Specific Ligase by Phosphorylation of Serine 20 in Ub

(A) Formation of a Ub(20pSer) dimer by Ube1, UBE2D3, and UBE3C followed by Coomassie staining. No more Ub dimer is detectable after the addition of the DUB vOTU after the 3-hr time point.

(B and C) Targeted MS using AQUA peptides on purified trimers formed by Ube1, UBE2D3, and UBE3C, using either unmodified Ub (B) or Ub (pSer20) (C). See also Figure S1.

of K6-linked Ub dimers by USP16 and USP21 was activated by Ser65 phosphorylation, but not by phosphorylation at Ser20 or Ser57 (Figures 5B and 6D).

The cleavage of K11-linked Ub dimers by OTUD3 was activated by phosphorylation of Ser20 in Ub, but it was inhibited by phosphorylation at Ser57 or Ser65 (Figure 5C). USP4 and USP5 cleaved K11-linked dimers of Ub with Ser57 or Ser65 phosphorylation, while USP6 cleaved K11-linked Ub dimers with Ser20 or Ser57 phosphorylation as efficiently as unmodified K11-linked dimers of Ub, but it was less efficient at cleaving K11-linked dimers of Ub (pSer65) (Figure 5C). However, the results for K11 chains with Ser65 phosphorylation must be interpreted with caution, because these chains contained a substantial amount of K63-linked Ub (pSer65) (Figure S4; Data S1).

The cleavage of K29-linked dimers by USP2 was activated by phosphorylation at Ser57, but the cleavage of K29-linked dimers of Ub by USP2 was deactivated by Ser65 phosphorylation (Figures 5D and 6E). The USP16-mediated cleavage of K33-linked Ub dimers was specifically enhanced by phosphorylation at Ser65 of Ub (Figures 5E and 6F).

The cleavage of K48-linked dimers of Ub by OTU family DUBs (OTUB1 and OTUB2) and several USP family DUBs (USP4, USP16, USP21, and VCPIP1) was activated by phosphorylation of Ser65, but it was deactivated by phosphorylation at Ser20 or Ser57 (Figures 5F and 6G). The cleavage of K63-linked Ub by USP8 and USP36 was severely attenuated by phosphorylation of Ser65 in Ub, but phosphorylation at Ser20 or Ser57 had little effect on cleavage of K63-linked Ub by these enzymes (Figures 5G and 6H).

Our data also define how each Ub phosphorylation alters the specificity with which DUBs cleave different Ub linkages (Figures 5 and 6). Ser20 phosphorylation increased OTUD3-mediated cleavage of K11-linked Ub, but it decreased OTUD3-mediated cleavage of K48 linkage (Figure 6I). Ser20 phosphorylation increased USP8-mediated cleavage of K6-linked Ub, but it decreased the USP8-mediated cleavage of K11 and K48 linkages (Figure 6J).

Ser57 phosphorylation increased USP2-mediated cleavage of K29-linked Ub, but it decreased USP2-mediated cleavage of K6 and K48 linkages (Figure 6K). Ser57 phosphorylation increased USP6-mediated cleavage of K11-linked Ub, but it decreased USP6-mediated cleavage of K6, K33, K48, and K63 linkages (Figure 6L). Ser57 phosphorylation increased USP8-mediated cleavage of K6-linked Ub, but it decreased USP8-mediated cleavage of K33 and K48 linkages (Figure 6M).

Ser65 phosphorylation increased OTUB2-mediated cleavage of K48-linked Ub, but it decreased OTUB2-mediated cleavage of K63 linkages (Figure 6N). Ser65 phosphorylation increased USP4-mediated cleavage of K11- and K48-linked Ub, but it decreased USP4-mediated cleavage of K6-, K29-, and K33-linked Ub (Figure 6O).

DISCUSSION

We report the synthesis of multi-milligram quantities of Ub phosphorylated at position 20, 57, and 65 and the structure of Ub phosphorylated at Ser20. We report the assembly of K6-, K11-, K33-, K48-, and K63-linked Ub bearing phosphorylation at Ser20, Ser57, or Ser65 and the assembly of K29-linked Ub bearing Ser57 or Ser65 phosphorylation. While we have only sampled a small subset of possible E3 ligase activities on phosphoubiquitin species, our data demonstrate that the site-specific phosphorylation of Ub can control the extent and isomeric composition of Ub chains synthesized and that phosphorylation at distinct sites on Ub have distinct effects on Ub polymer

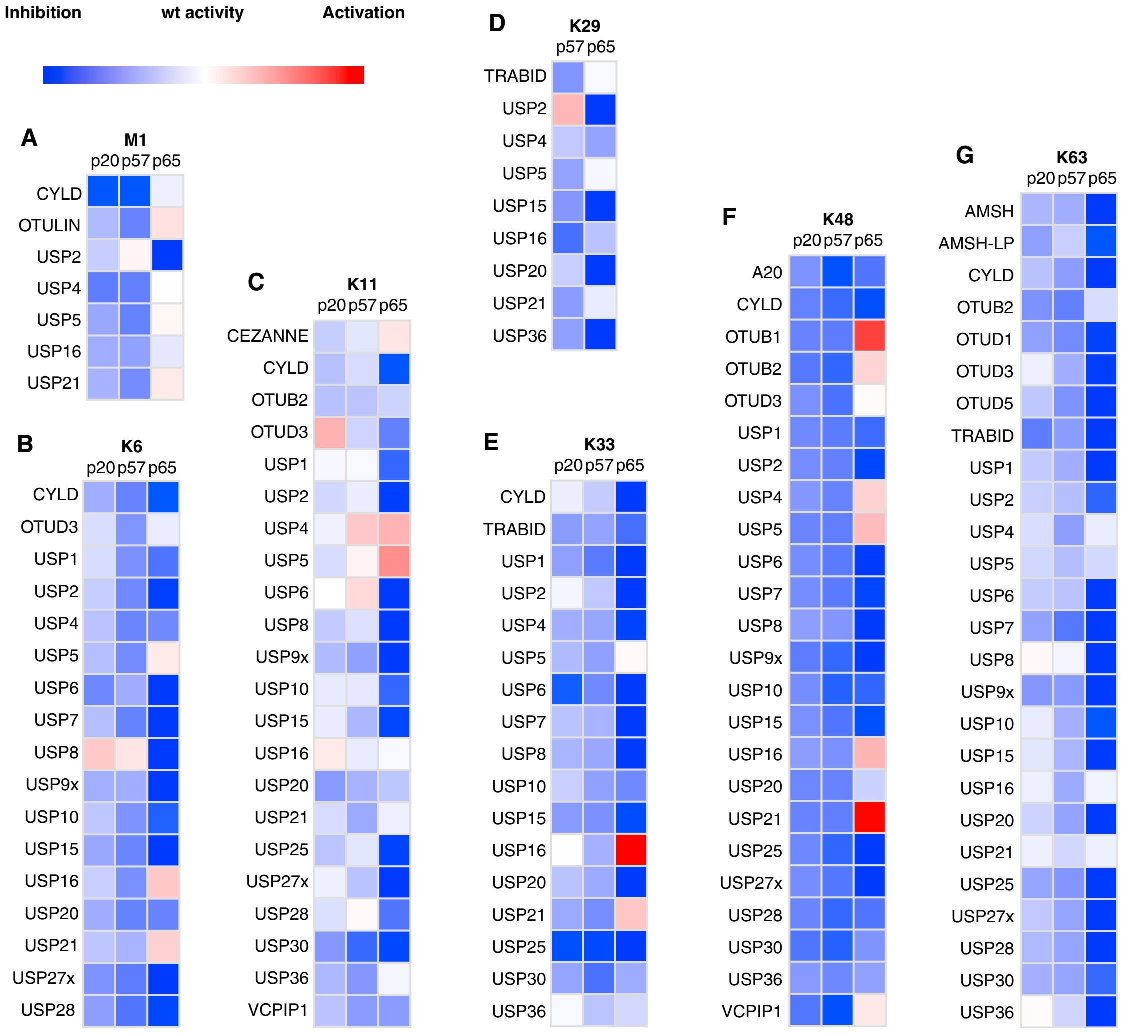

Figure 5. Profiling DUB Activity on Isomeric Phospho-Ub Dimers

(A–G) Purified dimers of Ub, Ub (pSer20), Ub (pSer57), and Ub (pSer65) from each linkage assembly reaction (Figure 3) were used to profile deubiquitnase acivity using MALDI-TOF MS with an ^{15}N-labeled Ub internal standard. The activity of each DUB on each phosphorylated Ub dimer, relative to an unmodified Ub dimer of the same linkage, is color coded from blue (no cleavage) under the assay conditions to white (same activity as unmodified Ub) to red (increased cleavage compared to unmodified Ub). (A) M1 Ub dimers, (B) K6 Ub dimers, (C) K11 Ub dimers, (D) K29 Ub dimers, (E) K33 Ub dimers, (F) K48 Ub dimers, and (G) K63 Ub dimers are shown.

See also Figures S3 and S4, Tables S2 and S3, and Data S1.

synthesis. The most striking observation from the chain assembly experiments is the change in linkage specificity of UBE3C upon phosphorylation at Ser20 of Ub; this enzyme assembles K29 and K48 linkages with comparable efficiency when using Ub as a substrate, but it primarily assembles K48 chains with a Ub (pSer20) substrate (Figure 6B).

UBE3C associates with the proteasome (Chu et al., 2013). In response to proteolytic stress, UBE3C is reported to ubiquitinate Rpn13 (a proteasome-resident Ub-binding protein) with K29-linked chains and decrease the proteasome's ability to bind and degrade Ub-conjugated proteins (Besche et al., 2014). Our results suggest that the phosphorylation of Ser20 in Ub may shut down the formation of K29-linked Ub chains formed by UBE3C, and this may enable the proteasome to resume proteolysis and aid cellular recovery following proteolytic stress.

Our data provide a wealth of new information on the consequences of Ub phosphorylation on DUB activity. Previous work investigated the effects of Ser65 phosphorylation on a single preferred substrate of five DUBs (Wauer et al., 2015), which does not illuminate the effects of phosphorylation on DUB isopeptide linkage specificity. In contrast, we have defined the effects of phosphorylation at Ser65 (Figure 7A) on the isopeptide linkage specificity of 31 DUBs. Moreover, we have defined the consequences of Ser20 and Ser57 phosphorylation (Figure 7A) for the DUB-mediated cleavage of each Ub linkage. Our data reveal that (1) phosphorylation at distinct sites in Ub can have different effects on the cleavage of a particular Ub linkage isomer

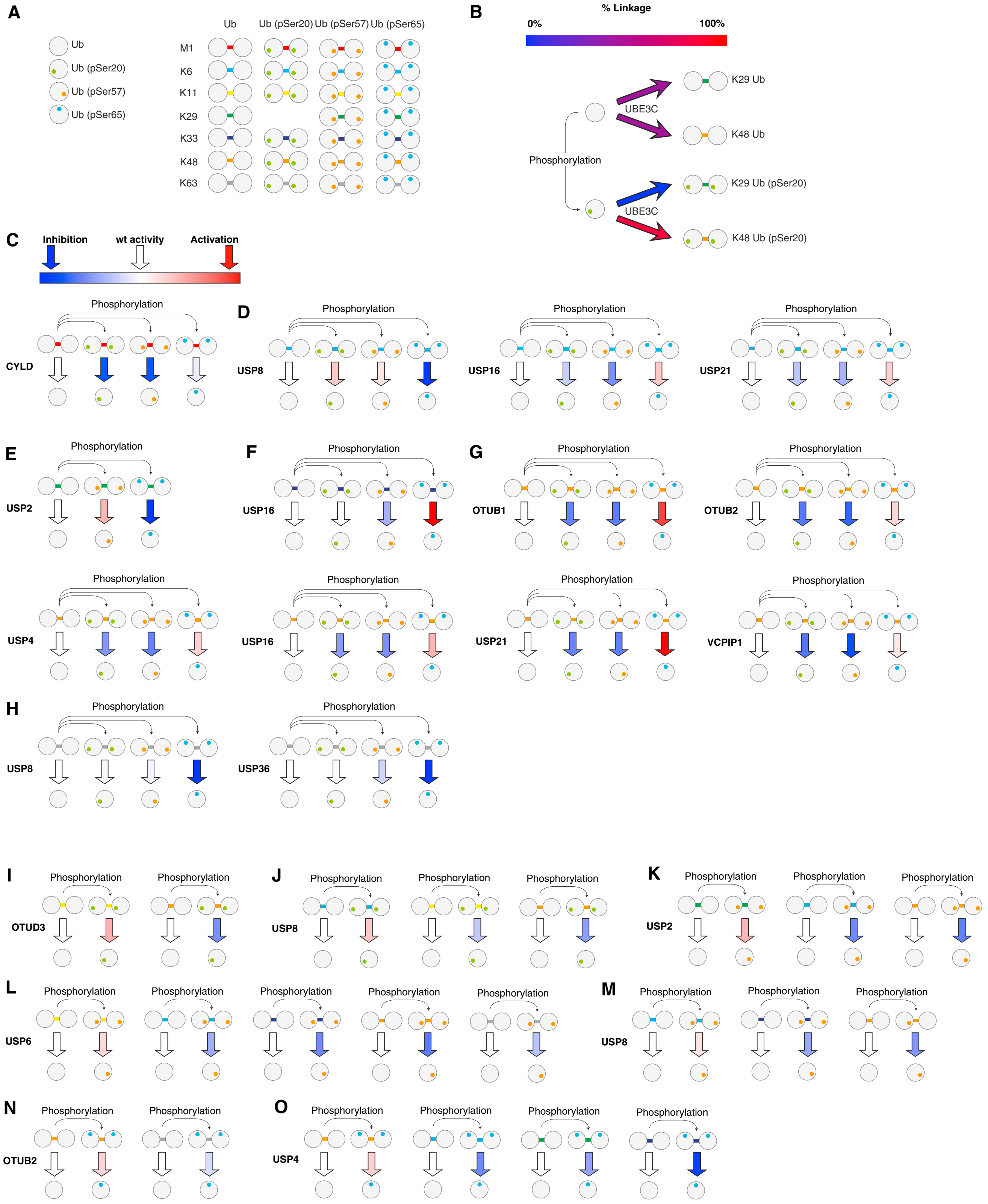

(legend on next page)

by a DUB, and that (2) phosphorylation at a single site in Ub can have a different effect on the cleavage of different Ub linkage isomers by a particular DUB.

DUBs bind to their substrates through a distal Ub-binding site (which binds the Ub that yields a free carboxylate upon [iso]peptide cleavage) and a proximal binding site (which yields a free amine upon cleavage) (Mevissen et al., 2013). The distal binding site binds Ub in a well-defined orientation, while the proximal site may allow Ub binding in several orientations to facilitate the cleavage of different linkage isomers. For several DUBs (Figure 6), we observed that the cleavage of a particular Ub linkage isomer is affected differently by phosphorylation at distinct sites on Ub in the dimer. Phosphorylation may affect the binding of Ub to the proximal binding site in a DUB, the distal binding site, or to both sites.

There are structural data for four DUBs with Ub in both the proximal and the distal binding sites (OTUB1 [Juang et al., 2012], AMSH-LP [Sato et al., 2008], OTULIN [Keusekotten et al., 2013], and CYLD [Sato et al., 2015]). In the structure of OTUB1 and AMSH, which cleave K48 and K63 linkages, respectively, all the serine phosphorylation sites in the distal Ub are solvent exposed and would not be predicted to directly affect binding or enzymatic activity (Figure 7B). In contrast, Ser20 and Ser57, but not Ser65, are close to the interface of the proximal Ub with OTUB1 (Figure 7C), and all sites of serine phosphorylation are close to the interface between AMSH-LP and the proximal Ub (Figure 7D).

Interestingly, there is a correlation between those phosphorylation sites that are close to the interface in the proximal Ub bound to these enzymes and their inhibition (Figures 5F and 6G). This is consistent with phosphorylation in the proximal Ub controlling the activity by inhibiting binding or activity (Figure 7E). The three phosphorylation sites in the Ub bound to the distal binding site of OTULIN are solvent exposed, consistent with phosphorylation of the distal Ub not controlling OTULIN activity. Ser20 and 57 in the proximal Ub are close to the distal Ub bound to OTULIN, while Ser65 on the proximal Ub is solvent exposed (Figure S5A). We propose that the OTULIN inhibition on M1 linkages observed for Ser20 and Ser57 phosphorylation (Figure 5A), but not for Ser65 phosphorylation, results from the simultaneous binding of the distal Ub and phosphorylated proximal Ub being incompatible on OTULIN. For CYLD, the three phosphorylation sites in the Ub bound to the distal binding site of CYLD are solvent exposed, and Ser20 and Ser57 also are solvent exposed in the proximal Ub, while Ser65 interacts with a flexible loop on CYLD (Figure S5B). Thus, the current structures of CYLD bound to diubiquitin do not provide an explanation of how phosphorylation controls Ub linkage cleavage.

For many DUBs tested, phosphorylation at one site inhibits the cleavage of all the Ub linkage isomers tested, with Ser65 phosphorylation of Ub leading to the greatest inhibition of DUB activity in most cases. The simplest explanation for these observations is that each phosphorylation has a defined effect on the activity of each DUB, by controlling Ub binding, regardless of the linkage isomer, at the distal binding site. This model is supported by the structure of Ub bound to the distal site of USP2 (Figure 7F), an enzyme that is inhibited from cleaving all Ub linkages by phosphorylation of Ser65 (Figure 5). Ser65 of the distal Ub is buried at the interface with USP2, and we suggest that phosphorylation of USP2 may control the cleavage of all Ub linkages by inhibiting binding of the distal Ub (Figure 7G).

For some DUBs (OTUD3, USP8, USP2, USP6, OTUB2, and, USP4) (Figures 6I–6O), the phosphorylation of Ub at a particular site can enhance the cleavage of a Ub linkage isomer, while phosphorylation at the same site in a different Ub linkage isomer can reduce the cleavage mediated by the same DUB. The differential cleavage of site-specifically phosphorylated Ub dimers as a function of linkage isomer is consistent with the phosphorylation affecting binding in the proximal Ub-binding site, where different isomers are bound in different orientations, rather than in the distal Ub-binding site, where Ub is bound in a common orientation. Consistent with this hypothesis, the structures of USP2, USP8, and OTUB2 with Ub bound in the distal binding site reveal that the relevant serine residues (Ser57 for USP2, Figure 6K; Ser20 and 57 for USP8, Figures 6J and 6M; and Ser65 for OTUB2, Figure 6N) are solvent exposed in the distal binding sites (Figure 7H). We conclude that specific phosphorylations on Ub may control the linkage specificity of DUBs by regulating the binding of selected isomers in the proximal binding site of the DUB (Figure 7I).

We demonstrate that the cleavage of a Ub linkage type may be regulated by phosphorylation of Ub and that phosphorylation at different sites in Ub can have distinct effects on the cleavage of a linkage. Since phosphorylation of serine residues in Ub commonly inhibits DUB activity but has less effect on the subset of Ub chain synthesis enzymes tested, we suggest that phosphorylation may regulate the levels of Ub chains, primarily through effects on DUB activity and specificity.

EXPERIMENTAL PROCEDURES

Purification of E1, E2, E3, DUB, ^{15}N-Ub, and Ub oligomers were by standard methods (Faggiano et al., 2016; Ritorto et al., 2014) and are described in the Supplemental Experimental Procedures. MS, MALDI-TOF assays (Ritorto

Figure 6. Synthesis of Phosphorylated Ub and Isomeric Phospho-Ub Chains Reveals the Effects of Ub Phosphorylation on E3 Ligase and DUB Specificity

(A) The phosphoubiquitin species synthesized. Ub is symbolized by a light gray circle on which each phosphorylated residue is symbolized by a smaller green (Ub [pSer20]), orange (Ub [pSer57]), or blue (Ub [pSer65]) circle. Each linkage is symbolized by a colored line as follows: M1, red; K6, blue; K11, yellow; K29, green; K33, dark blue; K48, orange; and K63, gray.

(B) Phosphorylation of Ser20 converts UBE3C from a dual-specificity E3 ligase to a K48-specific E3 ligase.

(C–H) Phosphorylation at Ser20, Ser57, and Ser65 of Ub has distinct effects on the cleavage of Ub linkages. The arrows indicate the efficiency of linkage cleavage for the indicated phosphoubiquitin dimer and are color coded as in Figure 5.

(I–O) Phosphorylation controls the specificity of Ub isomer cleavage by DUBs.

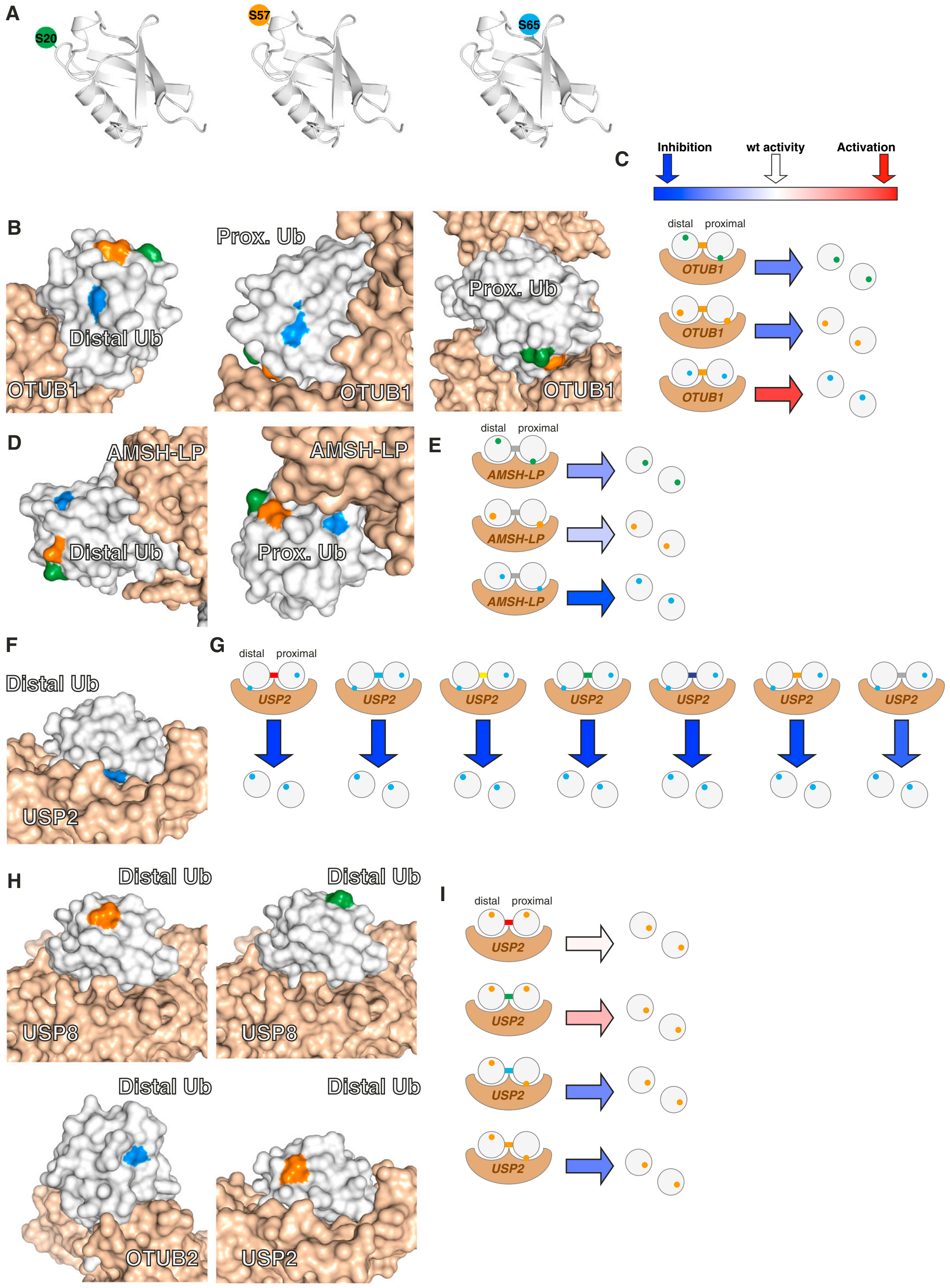

Figure 7. Structural Models for the Control of DUB Specificity by Phosphorylation

(A) Cartoon representation of Ub. The positions of the phosphate groups are represented by a green (pSer20), orange (pSer57), or blue (pSer65) circle. The same color scheme is used throughout the figure.

(B) The structure of OTUB1 in complex with proximal and distal Ub linked through a K48 isopeptide bond (PDB: 4DDG [Juang et al., 2012]). The position of each phosphorylation on the proximal and distal Ub is color coded.

(legend continued on next page)

et al., 2014), and targeted parallel reaction monitoring MS are detailed in the Supplemental Experimental Procedures.

Expression and Purification of Phosphorylated Ub Variants

BL21 ΔserB(DE3) cells containing pKW2 EF-Sep and pNHD-Ub (described in Rogerson et al., 2015) containing Ub-His_6, UbK48R-His_6, Ub20TAG-His_6, Ub20TAG:K48R-His_6, Ub57TAG-His_6, Ub57TAG:K48R-His_6, Ub65TAG-His_6, or Ub65TAG:K48R-His_6 were grown overnight at 37°C in terrific broth (TB) media containing 50 μg/ml chloramphenicol and 25 μg/ml tetracycline. The culture was diluted 1:100 into fresh TB media containing 25 μg/ml chloramphenicol and 12.5 μg/ml tetracycline and incubated at 37°C; once the OD_{600} reached 0.5, dexfosfoserine (pSer) (Bachem) was added to a final concentration of 2 mM. After 30 min of further incubation, additional pSer was added to the culture to reach a final concentration of 4 mM, and the culture was induced using 1 mM isopropyl β-D-1-thiogalactopyranoside (IPTG) at 37°C for 6 hr.

Cells were harvested by centrifugation, resuspended in 50 mM Tris-HCl (pH 7.4), 150 mM NaCl, 30 mM imidazole, 0.5 mg/ml lysozyme (Sigma), and 50 μg/ml DNase (Sigma), and lysed by sonication. The lysate was clarified by centrifugation at 39,000 × *g* for 30 min and filtration through a 0.4 μm polyethersulfone (PES) membrane. Ub was purified using nickel affinity chromatography (HisTrap HP column, GE Healthcare) with a linear gradient of imidazole (30–500 mM). The protein was dialysed overnight against 10 mM Tris-HCl at 4°C, the C-terminal His_6-tag was removed by incubating the sample with 15 μg/ml UCHL3 at 37°C for 5 hr, and Ub was further purified by ion exchange chromatography (MonoS 4.6/100 PE column, GE Healthcare), using an NaCl gradient (0–500 mM) in 50 mM ammonium acetate (pH 4.5). Pure fractions were confirmed by ESI-MS and pooled before overnight dialysis against 20 mM Tris-HCl (pH 7.4). The sample was then concentrated to ~15 mg/ml using Amicon Ultra-15 (3 kDa molecular weight cut off [MWCO]) centrifugal filter device (Millipore). All purifications were performed at 4°C.

Crystallographic Analysis of Ub (pSer20)

Purified Ub (pSer20) in 20 mM Tris (pH 7.4) was crystallized at 10 mg/ml in a hanging-drop setup using the vapor diffusion method. Crystals grew in 45% (w/v) PEG 400 and 100 mM Tris-HCl (pH 7.0), and they were vitrified in the same mother liquor. Data were collected at the ID-29 beamline at the European Synchrotron Radiation Facility (ESRF). The structure was determined by molecular replacement in Phaser (McCoy et al., 2007), using a search model of Ub (PDB: 1UBQ [Vijay-Kumar et al., 1987]) that lacked the last five flexible C-terminal residues. Model building and refinement were carried out in Coot (Emsley et al., 2010) and Phenix (Adams et al., 2010, 2011), respectively. The final statistics are shown in Table S1.

ACCESSION NUMBERS

The accession number for the crystal structure reported in this paper is PDB: 5K9P.

SUPPLEMENTAL INFORMATION

Supplemental Information includes Supplemental Experimental Procedures, five figures, three tables, and one data file and can be found with this article online at http://dx.doi.org/10.1016/j.celrep.2016.06.064.

AUTHOR CONTRIBUTIONS

J.W.C. defined the direction of research. N.H.-D. designed and performed all biochemical experiments and performed crystallographic analyses. J.P. performed MS with AQUA peptide standards experiments and V.D.C. performed DUB experiments under the supervision of M.T. A.K. provided reagents for biochemical experiments and purified ^{15}N-labelled Ub (pSer65). Y.A.K. provided reagents and advice for Ub chain purification together with Y.K. D.T.R performed initial cloning and expression of Ub(TAG) constructs with N.H.-D. All authors analyzed the data. N.H.-D. and J.W.C. wrote the paper with input from all authors.

ACKNOWLEDGMENTS

This work was supported by the Medical Research Council, UK (MC_U105181009 and MC_UP_A024_1008 to J.W.C. and MC_UU_12016/5 to M.T.). We are grateful to Laia Pedro-Roig, Syed Arif Abdul Rehman, Domagoj Baretić, Alex Bernt, Ksenia Rostislavleva, Satpal Virdee, Olga Perisic, Roger Williams, Leo James, Garib Murshudov, and Dario Alessi for assistance and/or advice. M.T. is grateful to the pharmaceutical companies supporting the Division of Signal Transduction Therapy (DSTT) (Astra Zeneca, Boehringer-Ingelheim, GlaxoSmithKline, Janssen Pharmaceutica, Merck KGaA, and Pfizer) and would like to thank the DNA cloning, protein production, DNA sequencing facility, and mass spectrometry teams of the MRC Protein Phosphorylation and Ubiquitylation Unit for their support.

Received: March 17, 2016
Revised: May 5, 2016
Accepted: June 14, 2016
Published: July 14, 2016

REFERENCES

Adams, P.D., Afonine, P.V., Bunkóczi, G., Chen, V.B., Davis, I.W., Echols, N., Headd, J.J., Hung, L.-W., Kapral, G.J., Grosse-Kunstleve, R.W., et al. (2010). PHENIX: a comprehensive Python-based system for macromolecular structure solution. Acta Crystallogr. D Biol. Crystallogr. *66*, 213–221.

Adams, P.D., Afonine, P.V., Bunkóczi, G., Chen, V.B., Echols, N., Headd, J.J., Hung, L.-W., Jain, S., Kapral, G.J., Grosse Kunstleve, R.W., et al. (2011). The Phenix software for automated determination of macromolecular structures. Methods *55*, 94–106.

(C) The site of phosphorylation on the proximal, but not distal, Ub may control the specificity of K48-linked Ub dimer cleavage by OTUB1. Ub is shown as a circle, and Ub linkages and phosphorylations are color coded as in Figure 6. Each DUB is shown in wheat. Arrow coloring is taken from Figure 5 (blue is inhibition and red is activation of DUB activity with respect to a non-phosphorylated Ub substrate).

(D) The structure of AMSH-LP in complex with proximal and distal Ub linked through a K63 isopeptide bond (PDB: 2ZNV [Sato et al., 2008]). Each phosphorylation on the proximal and distal Ub is color coded.

(E) The site of phosphorylation on the proximal, but not distal, Ub may control the specificity of K63-linked Ub dimer cleavage by AMSH-LP. Ub linkages are color coded as in Figure 6.

(F) The structure of USP2 in complex with Ub bound at the distal site (PDB: 2HD5 [Renatus et al., 2006]). The site of Ser65 phosphorylation is colored blue.

(G) Phosphorylation on the distal Ub in USP2 may inhibit cleavage of all Ub linkages. Linkages are color coded as in Figure 6.

(H) The structures of USP8 (PDB: 3N3K [Ernst et al., 2013]), OTUB2 (PDB:4FJV [Altun et al., 2015]), and USP2 (PDB: 2HD5 [Renatus et al., 2006]) bound to distal Ub. The sites of phosphorylation that activate the cleavage of one linkage by a DUB, while inhibiting the cleavage of another linkage by the same DUB, are shown for each complex with the distal Ub. The sites of phosphorylation lie far from the protein interface between the DUBs and the distal Ub, suggesting that, for these enzymes, specificity is controlled through the proximal Ub.

(I) Linkage specificity may be controlled by site-specific phosphorylation controlling proximal Ub binding in different orientations, which may be inhibitory or activating.

See also Figure S5.

Altun, M., Walter, T.S., Kramer, H.B., Herr, P., Iphöfer, A., Boström, J., David, Y., Komsany, A., Ternette, N., Navon, A., et al. (2015). The human otubain2-ubiquitin structure provides insights into the cleavage specificity of poly-ubiquitin-linkages. PLoS ONE *10*, e0115344.

Bennetzen, M.V., Larsen, D.H., Bunkenborg, J., Bartek, J., Lukas, J., and Andersen, J.S. (2010). Site-specific phosphorylation dynamics of the nuclear proteome during the DNA damage response. Mol. Cell. Proteomics *9*, 1314–1323.

Berndsen, C.E., and Wolberger, C. (2014). New insights into ubiquitin E3 ligase mechanism. Nat. Struct. Mol. Biol. *21*, 301–307.

Besche, H.C., Sha, Z., Kukushkin, N.V., Peth, A., Hock, E.-M., Kim, W., Gygi, S., Gutierrez, J.A., Liao, H., Dick, L., and Goldberg, A.L. (2014). Autoubiquitination of the 26S proteasome on Rpn13 regulates breakdown of ubiquitin conjugates. EMBO J. *33*, 1159–1176.

Bian, Y., Song, C., Cheng, K., Dong, M., Wang, F., Huang, J., Sun, D., Wang, L., Ye, M., and Zou, H. (2014). An enzyme assisted RP-RPLC approach for in-depth analysis of human liver phosphoproteome. J. Proteomics *96*, 253–262.

Bremm, A., Freund, S.M.V., and Komander, D. (2010). Lys11-linked ubiquitin chains adopt compact conformations and are preferentially hydrolyzed by the deubiquitinase Cezanne. Nat. Struct. Mol. Biol. *17*, 939–947.

Choudhary, C., Olsen, J.V., Brandts, C., Cox, J., Reddy, P.N.G., Böhmer, F.D., Gerke, V., Schmidt-Arras, D.-E., Berdel, W.E., Müller-Tidow, C., et al. (2009). Mislocalized activation of oncogenic RTKs switches downstream signaling outcomes. Mol. Cell *36*, 326–339.

Chu, B.W., Kovary, K.M., Guillaume, J., Chen, L.-C., Teruel, M.N., and Wandless, T.J. (2013). The E3 ubiquitin ligase UBE3C enhances proteasome processivity by ubiquitinating partially proteolyzed substrates. J. Biol. Chem. *288*, 34575–34587.

Clague, M.J., Heride, C., and Urbé, S. (2015). The demographics of the ubiquitin system. Trends Cell Biol. *25*, 417–426.

Corti, O., Lesage, S., and Brice, A. (2011). What genetics tells us about the causes and mechanisms of Parkinson's disease. Physiol. Rev. *91*, 1161–1218.

Emsley, P., Lohkamp, B., Scott, W.G., and Cowtan, K. (2010). Features and development of Coot. Acta Crystallogr. D Biol. Crystallogr. *66*, 486–501.

Ernst, A., Avvakumov, G., Tong, J., Fan, Y., Zhao, Y., Alberts, P., Persaud, A., Walker, J.R., Neculai, A.-M., Neculai, D., et al. (2013). A strategy for modulation of enzymes in the ubiquitin system. Science *339*, 590–595.

Faggiano, S., Alfano, C., and Pastore, A. (2016). The missing links to link ubiquitin: Methods for the enzymatic production of polyubiquitin chains. Anal. Biochem. *492*, 82–90.

Hasegawa, H., and Holm, L. (2009). Advances and pitfalls of protein structural alignment. Curr. Opin. Struct. Biol. *19*, 341–348.

Heinemann, I.U., Rovner, A.J., Aerni, H.R., Rogulina, S., Cheng, L., Olds, W., Fischer, J.T., Söll, D., Isaacs, F.J., and Rinehart, J. (2012). Enhanced phosphoserine insertion during Escherichia coli protein synthesis via partial UAG codon reassignment and release factor 1 deletion. FEBS Lett. *586*, 3716–3722.

Herhaus, L., and Dikic, I. (2015). Expanding the ubiquitin code through post-translational modification. EMBO Rep. *16*, 1071–1083.

Hospenthal, M.K., Freund, S.M.V., and Komander, D. (2013). Assembly, analysis and architecture of atypical ubiquitin chains. Nat. Struct. Mol. Biol. *20*, 555–565.

Juang, Y.C., Landry, M.C., Sanches, M., Vittal, V., Leung, C.C., Ceccarelli, D.F., Mateo, A.R., Pruneda, J.N., Mao, D.Y., Szilard, R.K., et al. (2012). OTUB1 co-opts Lys48-linked ubiquitin recognition to suppress E2 enzyme function. Mol. Cell *45*, 384–397.

Kaiser, S.E., Riley, B.E., Shaler, T.A., Trevino, R.S., Becker, C.H., Schulman, H., and Kopito, R.R. (2011). Protein standard absolute quantification (PSAQ) method for the measurement of cellular ubiquitin pools. Nat. Methods *8*, 691–696.

Kane, L.A., Lazarou, M., Fogel, A.I., Li, Y., Yamano, K., Sarraf, S.A., Banerjee, S., and Youle, R.J. (2014). PINK1 phosphorylates ubiquitin to activate Parkin E3 ubiquitin ligase activity. J. Cell Biol. *205*, 143–153.

Kazlauskaite, A., Kondapalli, C., Gourlay, R., Campbell, D.G., Ritorto, M.S., Hofmann, K., Alessi, D.R., Knebel, A., Trost, M., and Muqit, M.M.K. (2014). Parkin is activated by PINK1-dependent phosphorylation of ubiquitin at Ser65. Biochem. J. *460*, 127–139.

Keusekotten, K., Elliott, P.R., Glockner, L., Fiil, B.K., Damgaard, R.B., Kulathu, Y., Wauer, T., Hospenthal, M.K., Gyrd-Hansen, M., Krappmann, D., et al. (2013). OTULIN antagonizes LUBAC signaling by specifically hydrolyzing Met1-linked polyubiquitin. Cell *153*, 1312–1326.

Kirkpatrick, D.S., Hathaway, N.A., Hanna, J., Elsasser, S., Rush, J., Finley, D., King, R.W., and Gygi, S.P. (2006). Quantitative analysis of in vitro ubiquitinated cyclin B1 reveals complex chain topology. Nat. Cell Biol. *8*, 700–710.

Komander, D., and Rape, M. (2012). The ubiquitin code. Annu. Rev. Biochem. *81*, 203–229.

Komander, D., Clague, M.J., and Urbé, S. (2009). Breaking the chains: structure and function of the deubiquitinases. Nat. Rev. Mol. Cell Biol. *10*, 550–563.

Kondapalli, C., Kazlauskaite, A., Zhang, N., Woodroof, H.I., Campbell, D.G., Gourlay, R., Burchell, L., Walden, H., Macartney, T.J., Deak, M., et al. (2012). PINK1 is activated by mitochondrial membrane potential depolarization and stimulates Parkin E3 ligase activity by phosphorylating Serine 65. Open Biol. *2*, 120080.

Koyano, F., Okatsu, K., Kosako, H., Tamura, Y., Go, E., Kimura, M., Kimura, Y., Tsuchiya, H., Yoshihara, H., Hirokawa, T., et al. (2014). Ubiquitin is phosphorylated by PINK1 to activate parkin. Nature *510*, 162–166.

Kristariyanto, Y.A., Abdul Rehman, S.A., Campbell, D.G., Morrice, N.A., Johnson, C., Toth, R., and Kulathu, Y. (2015). K29-selective ubiquitin binding domain reveals structural basis of specificity and heterotypic nature of k29 polyubiquitin. Mol. Cell *58*, 83–94.

Kulathu, Y., and Komander, D. (2012). Atypical ubiquitylation - the unexplored world of polyubiquitin beyond Lys48 and Lys63 linkages. Nat. Rev. Mol. Cell Biol. *13*, 508–523.

Kumar, K.S.A., Spasser, L., Erlich, L.A., Bavikar, S.N., and Brik, A. (2010). Total chemical synthesis of di-ubiquitin chains. Angew. Chem. Int. Ed. Engl. *49*, 9126–9131.

Kumar, A., Aguirre, J.D., Condos, T.E.C., Martinez-Torres, R.J., Chaugule, V.K., Toth, R., Sundaramoorthy, R., Mercier, P., Knebel, A., Spratt, D.E., et al. (2015). Disruption of the autoinhibited state primes the E3 ligase parkin for activation and catalysis. EMBO J. *34*, 2506–2521.

Lai, Y.-C., Kondapalli, C., Lehneck, R., Procter, J.B., Dill, B.D., Woodroof, H.I., Gourlay, R, Peggie, M., Macartney, T.J., Corti, O., et al. (2015). Phosphoproteomic screening identifies Rab GTPases as novel downstream targets of PINK1. EMBO J. *34*, 2840–2861.

Lazarou, M., Sliter, D.A., Kane, L.A., Sarraf, S.A., Wang, C., Burman, J.L., Sideris, D.P., Fogel, A.I., and Youle, R.J. (2015). The ubiquitin kinase PINK1 recruits autophagy receptors to induce mitophagy. Nature *524*, 309–314.

Lundby, A., Secher, A., Lage, K., Nordsborg, N.B., Dmytriyev, A., Lundby, C., and Olsen, J.V. (2012). Quantitative maps of protein phosphorylation sites across 14 different rat organs and tissues. Nat. Commun. *3*, 876.

Malik, R., Lenobel, R., Santamaria, A., Ries, A., Nigg, E.A., and Körner, R. (2009). Quantitative analysis of the human spindle phosphoproteome at distinct mitotic stages. J. Proteome Res. *8*, 4553–4563.

Manes, N.P., Dong, L., Zhou, W., Du, X., Reghu, N., Kool, A.C., Choi, D., Bailey, C.L., Petricoin, E.F., Liotta, L.A., and Popov, S.G. (2011). Discovery of mouse spleen signaling responses to anthrax using label-free quantitative phosphoproteomics via mass spectrometry. Mol. Cell. Proteomics *10*, M110.000927.

McCoy, A.J., Grosse-Kunstleve, R.W., Adams, P.D., Winn, M.D., Storoni, L.C., and Read, R.J. (2007). Phaser crystallographic software. J. Appl. Cryst. *40*, 658–674.

Mevissen, T.E., Hospenthal, M.K., Geurink, P.P., Elliott, P.R., Akutsu, M., Arnaudo, N., Ekkebus, R., Kulathu, Y., Wauer, T., El Oualid, F., et al. (2013). OTU deubiquitinases reveal mechanisms of linkage specificity and enable ubiquitin chain restriction analysis. Cell *154*, 169–184.

Michel, M.A., Elliott, P.R., Swatek, K.N., Simicek, M., Pruneda, J.N., Wagstaff, J.L., Freund, S.M.V., and Komander, D. (2015). Assembly and specific recognition of k29- and k33-linked polyubiquitin. Mol. Cell *58*, 95–109.

Ohtake, F., Saeki, Y., Sakamoto, K., Ohtake, K., Nishikawa, H., Tsuchiya, H., Ohta, T., Tanaka, K., and Kanno, J. (2015). Ubiquitin acetylation inhibits polyubiquitin chain elongation. EMBO Rep. *16*, 192–201.

Ordureau, A., Heo, J.-M., Duda, D.M., Paulo, J.A., Olszewski, J.L., Yanishevski, D., Rinehart, J., Schulman, B.A., and Harper, J.W. (2015). Defining roles of PARKIN and ubiquitin phosphorylation by PINK1 in mitochondrial quality control using a ubiquitin replacement strategy. Proc. Natl. Acad. Sci. USA *112*, 6637–6642.

Park, H.-S., Hohn, M.J., Umehara, T., Guo, L.-T., Osborne, E.M., Benner, J., Noren, C.J., Rinehart, J., and Söll, D. (2011). Expanding the genetic code of Escherichia coli with phosphoserine. Science *333*, 1151–1154.

Phanstiel, D.H., Brumbaugh, J., Wenger, C.D., Tian, S., Probasco, M.D., Bailey, D.J., Swaney, D.L., Tervo, M.A., Bolin, J.M., Ruotti, V., et al. (2011). Proteomic and phosphoproteomic comparison of human ES and iPS cells. Nat. Methods *8*, 821–827.

Renatus, M., Parrado, S.G., D'Arcy, A., Eidhoff, U., Gerhartz, B., Hassiepen, U., Pierrat, B., Riedl, R., Vinzenz, D., Worpenberg, S., and Kroemer, M. (2006). Structural basis of ubiquitin recognition by the deubiquitinating protease USP2. Structure *14*, 1293–1302.

Ritorto, M.S., Ewan, R., Perez-Oliva, A.B., Knebel, A., Buhrlage, S.J., Wightman, M., Kelly, S.M., Wood, N.T., Virdee, S., Gray, N.S., et al. (2014). Screening of DUB activity and specificity by MALDI-TOF mass spectrometry. Nat. Commun. *5*, 4763.

Rogerson, D.T., Sachdeva, A., Wang, K., Haq, T., Kazlauskaite, A., Hancock, S.M., Huguenin-Dezot, N., Muqit, M.M.K., Fry, A.M., Bayliss, R., and Chin, J.W. (2015). Efficient genetic encoding of phosphoserine and its nonhydrolyzable analog. Nat. Chem. Biol. *11*, 496–503.

Sato, Y., Yoshikawa, A., Yamagata, A., Mimura, H., Yamashita, M., Ookata, K., Nureki, O., Iwai, K., Komada, M., and Fukai, S. (2008). Structural basis for specific cleavage of Lys 63-linked polyubiquitin chains. Nature *455*, 358–362.

Sato, Y., Goto, E., Shibata, Y., Kubota, Y., Yamagata, A., Goto-Ito, S., Kubota, K., Inoue, J., Takekawa, M., Tokunaga, F., and Fukai, S. (2015). Structures of CYLD USP with Met1- or Lys63-linked diubiquitin reveal mechanisms for dual specificity. Nat. Struct. Mol. Biol. *22*, 222–229.

Sharma, K., D'Souza, R.C.J., Tyanova, S., Schaab, C., Wiśniewski, J.R., Cox, J., and Mann, M. (2014). Ultradeep human phosphoproteome reveals a distinct regulatory nature of Tyr and Ser/Thr-based signaling. Cell Rep. *8*, 1583–1594.

Shiba-Fukushima, K., Imai, Y., Yoshida, S., Ishihama, Y., Kanao, T., Sato, S., and Hattori, N. (2012). PINK1-mediated phosphorylation of the Parkin ubiquitin-like domain primes mitochondrial translocation of Parkin and regulates mitophagy. Sci. Rep. *2*, 1002.

Steinbrecher, T., Latzer, J., and Case, D.A. (2012). Revised AMBER parameters for bioorganic phosphates. J. Chem. Theory Comput. *8*, 4405–4412.

Swaney, D.L., Rodríguez-Mias, R.A., and Villén, J. (2015). Phosphorylation of ubiquitin at Ser65 affects its polymerization, targets, and proteome-wide turnover. EMBO Rep. *16*, 1131–1144.

Valente, E.M., Abou-Sleiman, P.M., Caputo, V., Muqit, M.M.K., Harvey, K., Gispert, S., Ali, Z., Del Turco, D., Bentivoglio, A.R., Healy, D.G., et al. (2004). Hereditary early-onset Parkinson's disease caused by mutations in PINK1. Science *304*, 1158–1160.

Vijay-Kumar, S., Bugg, C.E., and Cook, W.J. (1987). Structure of ubiquitin refined at 1.8 A resolution. J. Mol. Biol. *194*, 531–544.

Villén, J., Beausoleil, S.A., Gerber, S.A., and Gygi, S.P. (2007). Large-scale phosphorylation analysis of mouse liver. Proc. Natl. Acad. Sci. USA *104*, 1488–1493.

Virdee, S., Ye, Y., Nguyen, D.P., Komander, D., and Chin, J.W. (2010). Engineered diubiquitin synthesis reveals Lys29-isopeptide specificity of an OTU deubiquitinase. Nat. Chem. Biol. *6*, 750–757.

Wauer, T., Swatek, K.N., Wagstaff, J.L., Gladkova, C., Pruneda, J.N., Michel, M.A., Gersch, M., Johnson, C.M., Freund, S.M., and Komander, D. (2015). Ubiquitin Ser65 phosphorylation affects ubiquitin structure, chain assembly and hydrolysis. EMBO J. *34*, 307–325.

Zhou, H., Di Palma, S., Preisinger, C., Peng, M., Polat, A.N., Heck, A.J.R., and Mohammed, S. (2013). Toward a comprehensive characterization of a human cancer cell phosphoproteome. J. Proteome Res. *12*, 260–271.

Cell Reports, Volume *16*

Supplemental Information

Synthesis of Isomeric Phosphoubiquitin Chains Reveals that Phosphorylation Controls Deubiquitinase Activity and Specificity

Nicolas Huguenin-Dezot, Virginia De Cesare, Julien Peltier, Axel Knebel, Yosua Adi Kristaryianto, Daniel T. Rogerson, Yogesh Kulathu, Matthias Trost, and Jason W. Chin

1. Supplemental Figures and Legends

Supplemental Figure 1

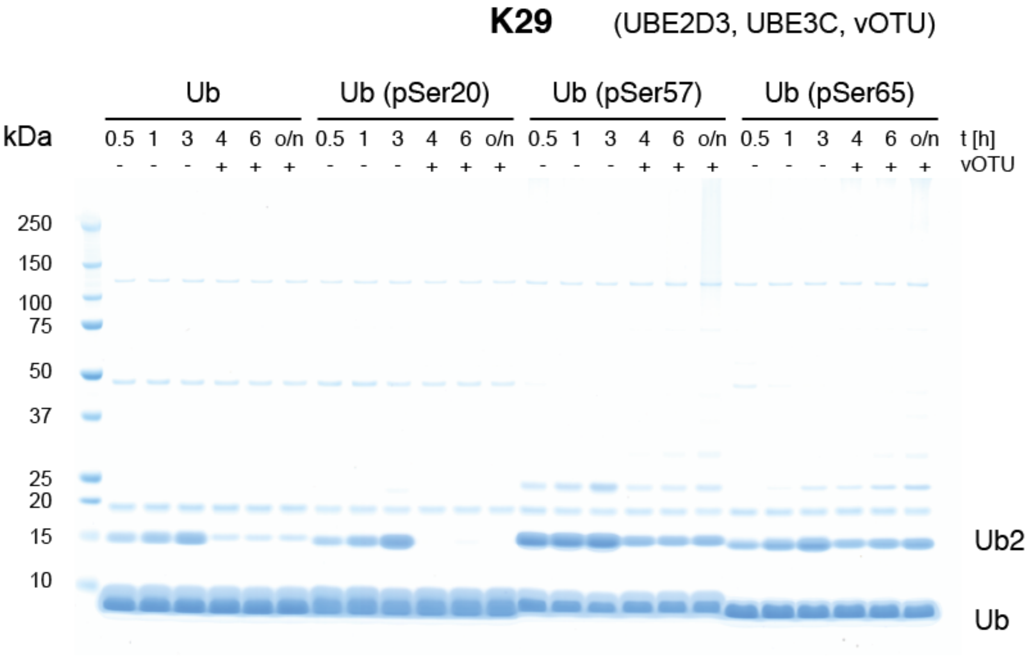

Figure S1, related to Figure 4. K29 chain assembly with phosphoubiquitins.
Full gel from **Figure 4**. K29 chains assembled with Ube1, UBE2D3, UBE3C and followed by coomassie staining. The DUB vOTU was added after the 3h time point. No more Ub dimer is detectable after addition of vOTU for Ub (20pSer).

Supplemental Figure 2

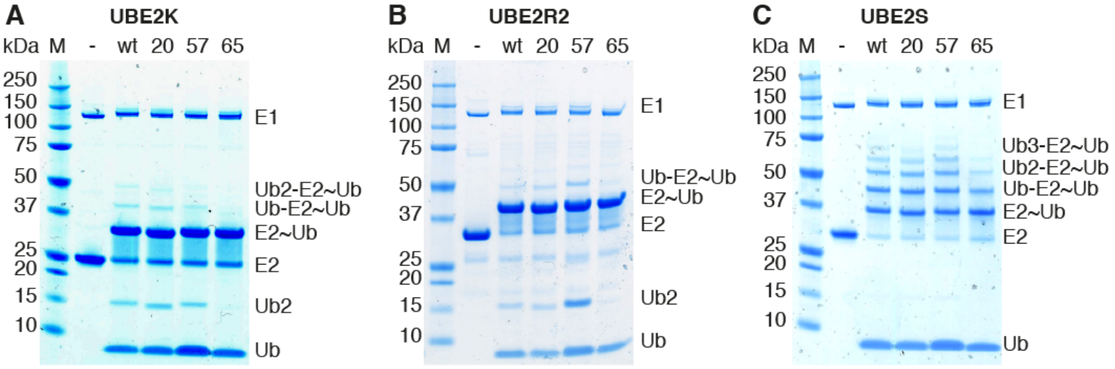

Figure S2, related to Figure 2. E2 charging with phosphoubiquitins.
Coommassie staining of reaction E2-charging reactions containing an E1 (Ube1), ATP, UBE2K **(A)** or UBE2R2 **(B)** or UBE2S **(C)** and either Ub, Ub (pSer20), Ub57(pSer) or Ub65(pSer) after 1h incubation at 30˚C. Ub: Ubiquitin. E2~Ub: Thioester-linked E2-ubiquitin pair. Ub-E2~Ub: Thioester-linked E2-ubiquitin pair with one covalently E2 attached ubiquitin. 20, 57 and 65 respectively designate the phosphorylated serine residue on ubiquitin.

Supplemental Figure 3

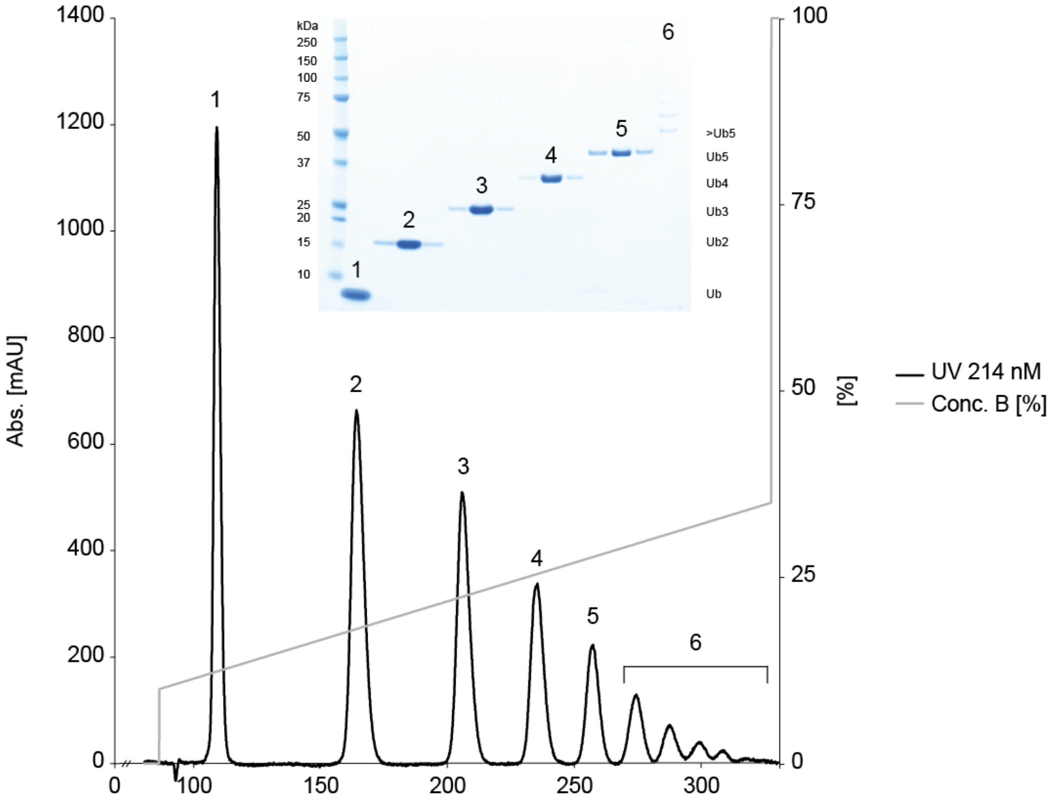

Figure S3, related to Figures 3 and 5. Example of ion-exchange chromatogram and purification of M1-linked Ub (pSer20) oligomers.
1: monomer, **2:** dimer, **3:** trimer, **4:** tetramer, **5:** pentamer, **6:** hexamer and higher molecular weight.

Supplemental Figure 4

A

M1				
**Phosphorylation**	–	**pSer20**	**pSer57**	**pSer65**
**Correct linkage**	96%	84%	94%	79%
**Other linkages**	–	9% K11	–	10% K33
**Deubiquitinase**	**% max**	**Activity on phosphorylated vs unphosphorylated dimers (%)**		
A20				
AMSH				
AMSH-LP				
CEZANNE				
CYLD	42 ± 7	20 ± 2	20 ± 4	92 ± 4
OTUB1				
OTUB2				
OTUD1				
OTUD3				
OTUD5				
OTULIN	92 ± 5	66 ± 5	41 ± 3	114 ± 5
TRABID				
USP1				
USP10				
USP15				
USP16	93 ± 4	61 ± 5	54 ± 7	88 ± 1
USP2	13 ± 8	76 ± 2	105 ± 8	0 ± 0
USP20				
USP21	97 ± 3	63 ± 12	45 ± 5	110 ± 3
USP25				
USP27x				
USP28				
USP30				
USP36				
USP4	31 ± 5	38 ± 5	39 ± 5	101 ± 3
USP5	100 ± 6	57 ± 8	40 ± 8	104 ± 4
USP6				
USP7				
USP8				
USP9x				
VCPIP1				

B

K6				
**Phosphorylation**	–	**pSer20**	**pSer57**	**pSer65**
**Correct linkage**	96%	89%	86%	77%
**Other linkages**	–	7% K11	–	11% K63
**Deubiquitinase**	**% max**	**Activity on phosphorylated vs unphosphorylated dimers (%)**		
A20				
AMSH				
AMSH-LP				
CEZANNE				
CYLD	95 ± 13	60 ± 4	41 ± 4	22 ± 1
OTUB1				
OTUB2				
OTUD1				
OTUD3	79 ± 5	84 ± 13	49 ± 2	91 ± 1
OTUD5				
OTULIN				
TRABID				
USP1	83 ± 6	83 ± 10	47 ± 4	33 ± 1
USP10	34 ± 8	72 ± 6	48 ± 3	25 ± 2
USP15	75 ± 2	57 ± 7	42 ± 4	0 ± 0
USP16	93 ± 5	76 ± 7	47 ± 3	127 ± 10
USP2	73 ± 10	75 ± 6	43 ± 3	9 ± 1
USP20	100 ± 3	60 ± 7	40 ± 9	41 ± 1
USP21	82 ± 4	72 ± 6	63 ± 1	123 ± 4
USP25	0			
USP27x	76 ± 15	48 ± 5	38 ± 6	0 ± 0
USP28	41 ± 4	53 ± 7	34 ± 2	13 ± 1
USP30				
USP36				
USP4	86 ± 8	70 ± 9	42 ± 6	43 ± 1
USP5	77 ± 8	68 ± 7	45 ± 4	111 ± 10
USP6	46 ± 9	43 ± 5	60 ± 3	0 ± 0
USP7	84 ± 5	69 ± 2	40 ± 1	0 ± 0
USP8	12 ± 7	127 ± 17	113 ± 7	0 ± 0
USP9x	16 ± 16	61 ± 10	60 ± 18	0 ± 0
VCPIP1				

C

K11				
**Phosphorylation**	–	**pSer20**	**pSer57**	**pSer65**
**Correct linkage**	95%	81%	95%	33%
**Other linkages**	–	Mix	–	62% K63
**Deubiquitinase**	**% max**	**Activity on phosphorylated vs unphosphorylated dimers (%)**		
A20				
AMSH				
AMSH-LP				
CEZANNE	48 ± 7	75 ± 9	88 ± 11	113 ± 2
CYLD	62 ± 25	69 ± 5	82 ± 3	19 ± 1
OTUB1				
OTUB2	21 ± 4	69 ± 3	71 ± 4	78 ± 1
OTUD1				
OTUD3	34 ± 9	136 ± 14	79 ± 9	39 ± 3
OTUD5				
OTULIN				
TRABID				
USP1	49 ± 4	96 ± 8	97 ± 9	28 ± 1
USP10	40 ± 3	90 ± 3	89 ± 10	28 ± 2
USP15	74 ± 3	90 ± 4	65 ± 5	11 ± 1
USP16	44 ± 4	111 ± 12	91 ± 18	97 ± 0
USP2	53 ± 17	80 ± 4	91 ± 5	8 ± 3
USP20	74 ± 2	51 ± 4	63 ± 2	72 ± 2
USP21	77 ± 8	82 ± 3	58 ± 3	92 ± 6
USP25	48 ± 7	71 ± 8	87 ± 14	11 ± 3
USP27x	18 ± 6	93 ± 8	71 ± 10	0 ± 0
USP28	43 ± 21	85 ± 8	103 ± 14	34 ± 1
USP30	64 ± 7	50 ± 5	29 ± 3	12 ± 8
USP36	100 ± 9	65 ± 9	49 ± 4	96 ± 3
USP4	41 ± 5	94 ± 13	127 ± 20	136 ± 15
USP5	47 ± 3	82 ± 4	106 ± 11	152 ± 9
USP6	39 ± 6	100 ± 10	119 ± 10	0 ± 0
USP7				
USP8	49 ± 6	74 ± 4	85 ± 5	0 ± 0
USP9x	61 ± 5	66 ± 3	54 ± 6	0 ± 0
VCPIP1	77 ± 4	71 ± 6	52 ± 5	52 ± 1

D

K29			
**Phosphorylation**	–	**pSer57**	**pSer65**
**Correct linkage**	–	75%	91%
**Other linkages**	–	17% K48	–
**Deubiquitinase**	**% max**	**Activity on phosphorylated vs unphosphorylated dimers (%)**	
A20			
AMSH			
AMSH-LP			
CEZANNE			
CYLD			
OTUB1			
OTUB2			
OTUD1			
OTUD3			
OTUD5			
OTULIN			
TRABID	100 ± 9	49 ± 4	98 ± 4
USP1			0
USP10			
USP15	33 ± 5	50 ± 3	3 ± 6
USP16	89 ± 8	32 ± 3	70 ± 2
USP2	23 ± 8	135 ± 11	0 ± 0
USP20	21 ± 17	76 ± 13	0 ± 0
USP21	83 ± 4	52 ± 2	90 ± 4
USP25			
USP27x			
USP28			
USP30			
USP36	30 ± 15	54 ± 8	0 ± 0
USP4	38 ± 4	73 ± 3	55 ± 3
USP5	82 ± 11	55 ± 6	96 ± 6
USP6			
USP7			
USP8			
USP9x			
VCPIP1			

E

K33				
**Phosphorylation**	–	**pSer20**	**pSer57**	**pSer65**
**Correct linkage**	99%	86%	99%	87%
**Other linkages**	–	9% K11	–	10% K63
**Deubiquitinase**	**% max**	**Activity on phosphorylated vs unphosphorylated dimers (%)**		
A20				
AMSH				
AMSH-LP				
CEZANNE				
CYLD	12 ± 16	92 ± 15	74 ± 4	0 ± 0
OTUB1				
OTUB2				
OTUD1				
OTUD3				
OTUD5				
OTULIN				
TRABID	51 ± 5	52 ± 5	55 ± 4	32 ± 1
USP1	100 ± 5	54 ± 5	37 ± 7	0 ± 0
USP10	13 ± 7	76 ± 10	54 ± 14	43 ± 8
USP15	37 ± 6	51 ± 5	47 ± 4	16 ± 1
USP16	39 ± 17	100 ± 9	62 ± 5	204 ± 4
USP2	44 ± 4	96 ± 17	73 ± 1	0 ± 0
USP20	21 ± 9	70 ± 8	59 ± 5	0 ± 0
USP21	69 ± 11	58 ± 3	46 ± 5	128 ± 2
USP25	38 ± 10	17 ± 2	14 ± 1	0 ± 0
USP27x				
USP28				
USP30	33 ± 10	56 ± 6	32 ± 16	59 ± 4
USP36	38 ± 8	97 ± 9	71 ± 11	81 ± 5
USP4	23 ± 7	60 ± 5	57 ± 6	11 ± 6
USP5	54 ± 4	66 ± 7	54 ± 4	103 ± 3
USP6	8 ± 8	24 ± 6	43 ± 19	0 ± 0
USP7	17 ± 11	71 ± 2	63 ± 1	0 ± 0
USP8	11 ± 16	64 ± 13	57 ± 8	0 ± 0
USP9x				
VCPIP1				

F

K48				
**Phosphorylation**	–	**pSer20**	**pSer57**	**pSer65**
**Correct linkage**	95%	80%	97%	86%
**Other linkages**	–	8% K6	–	6% K63
**Deubiquitinase**	**% max**	**Activity on phosphorylated vs unphosphorylated dimers (%)**		
A20	61 ± 8	48 ± 5	18 ± 2	34 ± 3
AMSH				
AMSH-LP				
CEZANNE	7			
CYLD	100 ± 8	39 ± 5	29 ± 1	17 ± 1
OTUB1	85 ± 4	41 ± 3	38 ± 2	179 ± 7
OTUB2	36 ± 9	36 ± 2	27 ± 3	121 ± 2
OTUD1				
OTUD3	89 ± 5	47 ± 2	32 ± 2	102 ± 15
OTUD5				
OTULIN				
TRABID				
USP1	93 ± 5	44 ± 3	37 ± 5	30 ± 1
USP10	66 ± 11	45 ± 2	26 ± 6	27 ± 1
USP15	88 ± 13	46 ± 9	34 ± 4	17 ± 1
USP16	83 ± 13	53 ± 8	47 ± 5	135 ± 9
USP2	80 ± 5	45 ± 3	39 ± 4	12 ± 1
USP20	88 ± 8	42 ± 6	40 ± 2	78 ± 3
USP21	96 ± 7	41 ± 5	39 ± 4	191 ± 13
USP25	78 ± 7	43 ± 2	27 ± 2	3 ± 1
USP27x	84 ± 20	45 ± 6	35 ± 3	0 ± 0
USP28	79 ± 4	42 ± 7	29 ± 3	34 ± 2
USP30	87 ± 9	34 ± 2	26 ± 6	48 ± 3
USP36	88 ± 6	51 ± 7	42 ± 5	54 ± 1
USP4	83 ± 8	50 ± 5	41 ± 4	122 ± 5
USP5	81 ± 11	41 ± 2	39 ± 2	133 ± 9
USP6	80 ± 10	45 ± 3	37 ± 4	0 ± 0
USP7	81 ± 10	45 ± 5	37 ± 3	12 ± 0
USP8	75 ± 5	53 ± 3	49 ± 4	0 ± 0
USP9x	41 ± 7	37 ± 2	28 ± 7	0 ± 0
VCPIP1	70 ± 12	35 ± 5	17 ± 2	112 ± 4

G

K63				
**Phosphorylation**	–	**pSer20**	**pSer57**	**pSer65**
**Correct linkage**	91%	93%	91%	94%
**Other linkages**	6% K6	–	5.5% K33	–
**Deubiquitinase**	**% max**	**Activity on phosphorylated vs unphosphorylated dimers (%)**		
A20				
AMSH	54 ± 3	64 ± 7	61 ± 5	0 ± 0
AMSH-LP	42 ± 10	53 ± 2	76 ± 9	20 ± 1
CEZANNE				
CYLD	75 ± 4	70 ± 6	52 ± 11	0 ± 0
OTUB1				
OTUB2	89 ± 4	47 ± 3	39 ± 4	83 ± 7
OTUD1	90 ± 10	54 ± 9	44 ± 12	10 ± 1
OTUD3	75 ± 6	93 ± 6	61 ± 7	8 ± 0
OTUD5	28 ± 6	72 ± 7	48 ± 1	0 ± 0
OTULIN				
TRABID	48 ± 6	38 ± 6	51 ± 2	0 ± 0
USP1	93 ± 5	74 ± 4	61 ± 7	0 ± 0
USP10	55 ± 4	91 ± 12	62 ± 8	21 ± 9
USP15	71 ± 3	88 ± 7	64 ± 5	0 ± 0
USP16	98 ± 13	93 ± 24	59 ± 7	94 ± 4
USP2	80 ± 5	76 ± 2	68 ± 8	27 ± 1
USP20	89 ± 10	78 ± 3	55 ± 9	0 ± 0
USP21	74 ± 6	93 ± 8	81 ± 13	93 ± 1
USP25	71 ± 3	56 ± 22	49 ± 4	0 ± 0
USP27x	73 ± 8	73 ± 2	56 ± 4	0 ± 0
USP28	74 ± 4	66 ± 9	56 ± 9	0 ± 0
USP30	32 ± 6	62 ± 6	56 ± 6	29 ± 5
USP36	53 ± 7	102 ± 5	79 ± 8	0 ± 0
USP4	80 ± 6	83 ± 8	53 ± 6	91 ± 3
USP5	77 ± 5	81 ± 4	68 ± 6	81 ± 2
USP6	83 ± 6	74 ± 2	70 ± 4	0 ± 0
USP7	100 ± 8	55 ± 3	36 ± 9	0 ± 0
USP8	53 ± 10	104 ± 16	95 ± 10	0 ± 0
USP9x	32 ± 5	50 ± 5	51 ± 8	0 ± 0
VCPIP1				

Figure S4, related to Figure 5. Quantification of DUB activity on phosphorylated ubiquitin dimers.

Purified dimers of Ub, Ub (pSer20), Ub (pSer57), and Ub (pSer65) from each linkage assembly reaction (**Figure 3**) were used to profile deubiquitnase activity using MALDI-TOF mass spectrometry with a ^{15}N-labelled Ub / phosphoubiquitin internal standard. **(A)** M1 Ub dimers, **(B)** K6 Ub dimers, **(C)** K11 Ub dimers, **(D)** K29 Ub dimers, **(E)** K33 Ub dimers, **(F)** K48 Ub dimers, **(G)** K63 Ub dimers. Relevant quantities of other linkages are indicated. The activity of each DUB is normalised to the activity of the most active DUB within the set of active DUBs (against a specific linkage) and displayed as yellow bars. The activity of the DUBs towards phosphorylated Ub dimers is expressed in % of the activity towards unmodified Ub dimers and colour coded from blue (no cleavage) to white (same activity as on unmodified Ub) to red (increased cleavage compared to unmodified Ub).

Supplemental Figure 5

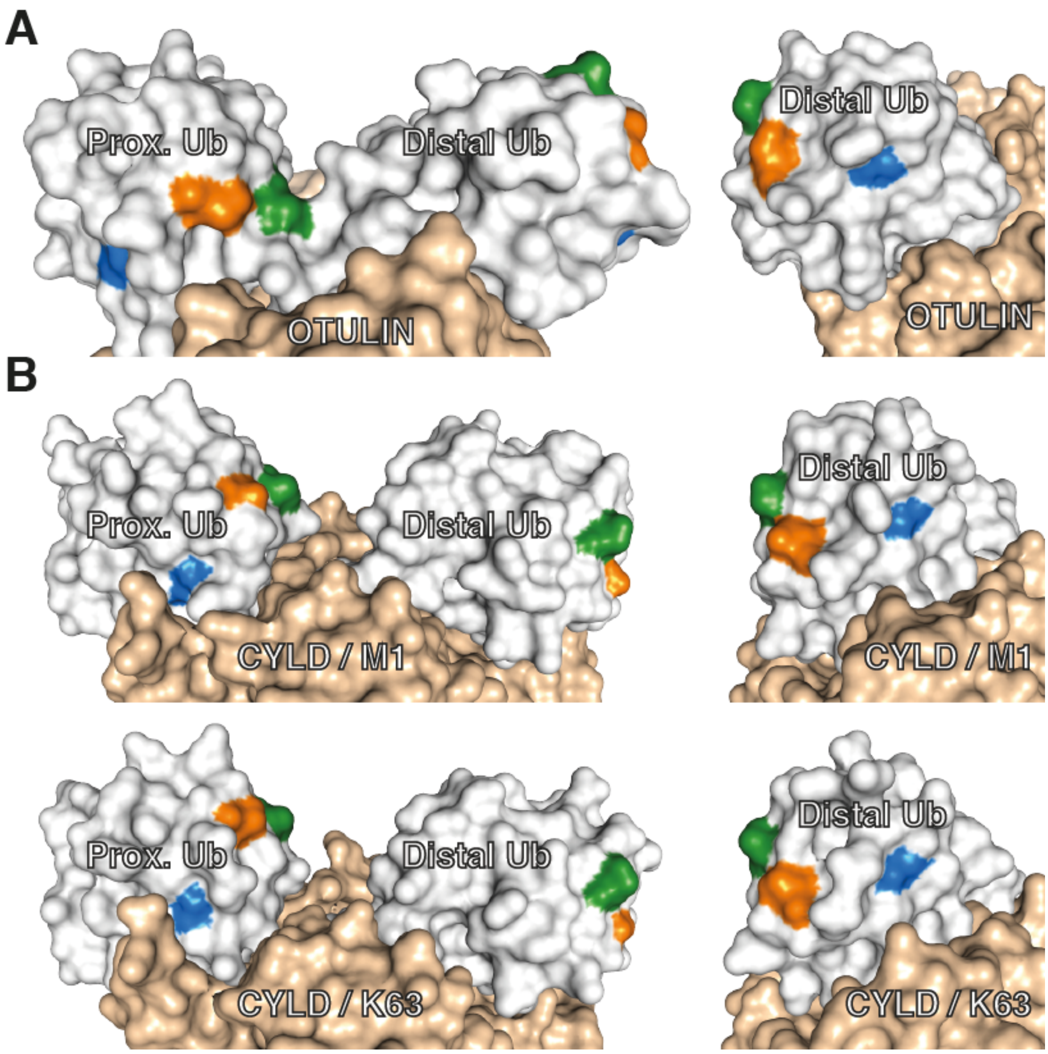

Figure S5, related to Figure 7. Structural models for the control of deubiquitinase specificity by phosphorylation.

(A) Structure of OTULIN in complex with a linear Ub dimer (PDB ID: 3ZNS (Keusekotten et al., 2013)). The position of each phosphorylation on the proximal and distal Ub is colour coded (**Figure 7A**). **(B)** Structure of CYLD in complex with a linear Ub dimer (PDB ID: 3WXF (Sato et al., 2015)) and a K63-linked ubiquitin dimer (PDB: 3WXG (Sato et al., 2015)). The position of each phosphorylation on the proximal and distal Ub is colour coded.

2. Supplemental Tables and Legends

Table S1, related to Figure 1. Data collection and refinement statistics for Ub (20pSer)

Table S2, related to Figures 2, 3 and 5. List of E2, E3 and DUB constructs used.

Table S3, related to Figure 5. DUB dilution table.

Supplemental Table 1, related to Figure 1. Data collection and refinement statistics for Ub (20pSer)

Ub20pSer	
**Wavelength [Å]**	0.96999
**Resolution range [Å]**	33.5 - 1.55 (1.58 - 1.55)
**Space group**	P 31 2 1
**Unit cell (a, b, c) (α, ß, γ) [°]**	(49.5205 49.5205 53.642) (90 90 120)
**Total reflections**	11448
**Unique reflections**	10779
**Multiplicity**	3.5 (3.2)
**Completeness (%)**	95.3 (91.3)
**Mean I/σ(I)**	6.4 (1.9)
**Wilson B-factor**	19.596
**Rmerge (all I+ and I-)**	0.102 (0.578)
**R-meas**	0.12 (0.69)
**Reflections used in refinement**	10754
**R-work**	0.1902
**R-free**	0.2194
**Number of non-hydrogen atoms**	691
Protein	638
**Protein residues**	76
**RMS(bonds)**	0.015
**RMS(angles)**	1.421
**Ramachandran favored (%)**	97.44
**Ramachandran allowed (%)**	2.56
**Ramachandran outliers (%)**	0
**Rotamer outliers (%)**	1.4
**Clashscore**	5.35
**Average B-factor**	27.32
Macromolecules	26.28
Solvent	39.82

Supplemental table 3, related to Figure 5. DUB dilution table.

**USP1**	**USP2**	**USP6**	**OTUB2**
240 ng/ul, K63	60 ng/ul, K63	3 ng/ul, K63	30 ng/ul, K63
**USP8**	**USP5**	**USP20**	**OTUD1**
144 ng/ul, K63	24 ng/ul, K63	60 ng/ul, K63	6 ng/ul, K63
**CYLD**	**OTUD5**	**AMSH**	**AMSH-LP**
240 ng/ul, K63	300 ng/ul, K63	60 ng/ul, K63	24 ng/ul, K63
**USP7**	**USP27x**	**Cezanne**	**USP21**
30 ng/ul, K11	120 ng/ul, K11	12 ng/ul, K11	58.4 ng/ul,K11
**USP9x**	**USP28**	**OTUD3**	**USP25**
170 ng/ul, K11	60 ng/ul, K11	60 ng/ul, K11	30 ng/ul, K11
**USP10**	**USP36**	**USP30**	**Otulin**
240 ng/ul, K11	750 ng/ul, K11	430 ng/ul, K48	1.2 ng/ul, M1
**VCPIP**	**A20**	**TRABID**	**OTUB1**
500 ng/ul, K48	60 ng/ul, K48	240 ng/ul, K48	300 ng/ul, K48
**USP4**	**USP16**	**USP15**	
120 ng/ul, K48	60 ng/ul, K48	16 ng/ul, K48	

3. Supplemental Data 1

Supplemental Data 1, related to Figure 3 and 5. Targeted mass spectrometry data and peptide sequences.

Supplemental Data 1.1 related to Figure 3 and 5. Targeted mass spectrometry using AQUA peptides on purified trimers.
M1 (assembled with Ube1, UBE2L3 and HOIP) on either Ub **(A)**, Ub (pSer20) **(B)**, Ub (pSer57) **(C)** or Ub (pSer65) **(D).**

Supplemental Data 1.2 related to Figure 3 and 5. Targeted mass spectrometry using AQUA peptides on purified trimers.
K6 chains (assembled with Ube1, UBE2L3, NleL) on either UbK48R **(A)**, UbK48R (pSer20) **(B)**, UbK48R (pSer57) **(C)** or UbK48R (pSer65) **(D)**.

Supplemental Data 1.3 related to Figure 3 and 5. Targeted mass spectrometry using AQUA peptides on total reactions.
Reactions with Ube1, UBE2L3 and NleL with either Ub **(A)**, Ub (pSer20) **(B)**, Ub (pSer57) **(C)** or Ub (pSer65) **(D).**

Supplemental Data 1.4 related to Figure 3 and 5. Targeted mass spectrometry using AQUA peptides on purified trimers.
K11 chains (assembled with Ube1, UBE2S-UBP and AMSH) on either Ub **(A)**, Ub (pSer20) **(B)**, Ub (pSer57) **(C)** or Ub (pSer65) **(D).**

Supplemental Data 1.5 related to Figure 3 and 5. Targeted mass spectrometry using AQUA peptides on purified trimers.
K29 chains (assembled with Ube1, UBE2D3, UBE3C and vOTU) on either Ub **(A)**, Ub (pSer57) **(B)** or Ub (pSer65) **(C)**.

Supplemental Data 1.6 related to Figure 3 and 5. Targeted mass spectrometry using AQUA peptides on total reactions.
Reactions with Ube1, UBE2D3 and UBE3C with either Ub **(A)**, Ub (pSer20) **(B)**, Ub (pSer57) **(C)** or Ub (pSer65) **(D).**

Supplemental Data 1.7 related to Figure 3 and 5. Targeted mass spectrometry using AQUA peptides on purified trimers.
K33 chains (assembled with Ube1, UBE2D1, AREL1, OTUB1 and Cezanne-EK) on either Ub **(A)**, Ub (pSer20) **(B)**, Ub (pSer57) **(C)** or Ub (pSer65) **(D).**

Supplemental Data 1.8 related to Figure 3 and 5. Targeted mass spectrometry using AQUA peptides on purified trimers.
K48 chains (assembled with Ube1, UBE2R1 or UBE2K) on either Ub **(A)**, Ub (pSer20) **(B)**, Ub (pSer57) **(C)**, Ub (pSer65) with UBE2R1 **(D)** or with UBE2K **(E).**

Supplemental Data 1.9 related to Figure 3 and 5. Targeted mass spectrometry using AQUA peptides on purified trimers.

K63 chains (assembled with Ube1, UBE2N and UBE2V1) on either Ub **(A)**, Ub (pSer20) **(B)**, Ub (pSer57) **(C)** or Ub (pSer65) **(D)**.

Supplemental Data 1.10 related to Figure 3 and 5. Targeted mass spectrometry using AQUA peptides on total reactions.
Reaction with Ube1, UBE2L3 and HHARI with either Ub **(A)**, Ub (pSer20) **(B)**, Ub (pSer57) **(C)** or Ub (pSer65) **(D)**.

Supplemental Data 1.11 related to Figure 3 and 5. List of isotopically labelled peptides used to monitor polyUb and phosphorylated polyUb linkages.
Signature peptides representing isopeptide-linked polyUb linkages are denoted by a subscript "GG" adjacent to the modified lysine. Signature peptides representing phosphorylated peptides are denotes by a "p" adjacent to modified serine. Isotopically labelled amino acids are denoted in blue and the oxidation state of Met-containing peptides is denoted by "ox". For each peptide, the optimal precursor ions to the HCD collision energy of 30% were selected for the PRM analysis.

4. Supplemental Experimental Procedures

E2 charging assay

E2-charging assays were carried out in 1 mL reactions by incubating 0.21 μM UBE1, 5 μM E2 and 5.8 μM Ub in 50 mM HEPES pH 7.4, 7.5 mM NaCl, 2 mM magnesium acetate, and 0.2 mM ATP. Reactions were incubated for 5 or 60 min at 30°C and stopped by addition of non-reducing LDS sample buffer (Invitrogen). Samples were analyzed by SDS-PAGE using 4–12% Bis-Tris gels (Invitrogen) and visualised using InstantBlue (Expedeon).

Ub chain assembly and purification

Ub chains were assembled in buffer containing 40 mM Tris pH 7.5, 10 mM $MgCl_2$, 0.6 mM DTT and 10 mM ATP. M1 chains: 0.8 mM Ub, 1 μM UBE1, 10 μM UBE2L3 and 10 μM HOIP. K6 chains: 1 mM UbK48R, 0.5 μM UBE1, 9.5 μM UBE2L3 and 12.4 μM NleL. K11 chains: 1.2 mM Ub, 1 μM UBE1, 40 μM UBE2S-UBP and 2 μM AMSH (added after 6 h). K29 chains: 1 mM Ub, 0.64 μM UBE1, 9.5 μM UBE2D3, 3 μM UBE3C and 2 μM vOTU. K33 chains: 1.1 mM Ub, 0.5 μM UBE1, 9 μM UBE2D1, 6.2 μM KIAA0317 and 5 μM OTUB1 + 20 μM Cezanne EK (added after 6 h). K48 chains: 0.95 mM Ub, 1 μM UBE1, 25 μM UBE2R1 or 25 μM UBE2K. K63 chains: 0.9 mM Ub, 1 μM UBE1, 10 μM UBE2N and 20 μM UBE2V1. Reactions were incubated at 30°C and terminated either by addition of reducing LDS sample buffer (for time course experiments) or dilution in 50 mM ammonium acetate pH 4.5 (for further purification). For time course experiments, samples were analyzed by SDS-PAGE. For Ub chains purification, the reactions were diluted in 50 mM ammonium acetate, pH 4.5 (to precipitate the enzymes) and filtered through a 0.22 μm PES membrane before loading on an ion exchange chromatography (ResourceS column, GE Healthcare). Ub chains were eluted using a gradient from 0 to 1 M NaCl in 50 mM ammonium acetate pH 4.5. Purity of each oligomer was assessed by running the peak edge and centre fractions on SDS-PAGE gels. Pure fractions for each oligomer were pooled, concentrated and buffer exchanged with 20 mM tris-HC pH 7.5.

Purification of deubiquitinases, E1s, E2s and E3s and DUBs

E1 and E2s: UBE1 was expressed as a 6His-tagged fusion protein in Sf21 cells and purified using Ni-NTA agarose. UBE2J2 and UBE2Z were expressed as a GST-tagged fusion protein in BL21 cells and affinity purified via its tag. All other E2 conjugating enzymes were expressed as His-tagged fusion proteins in BL21 cells and affinity purified over Ni-NTA-agarose **(Table S2A)**.

Expression and purification of E3 ligases: AREL1, HOIP, NleL and HHARI were expressed as GST-fusion proteins in BL21 DE3 cells, purified over GSH-agarose and recovered by protease treatment (TEV-protease or Prescission Protease) to sever to bond to the GST-tag. UBE3C (641 –end) was expressed as a GST-tagged fusion protein in Sf21 cells and essentially purified like the other ligases **(Table S2B)**.

Expression of DUB enzymes was performed as previously described (Ritorto et al., 2014). The proteins were produced in BL21 DE3 cells or Sf21 insect cells using standard procedures **(Table S2C)**.

Expression and purification of ^{15}N-Ub (pSer65)

Untagged full-length human Ub (1-76) was cloned into the pET-24 vector (DU20027) and expressed in E. coli BL21 (DE3) cells grown in 50 mM Na_2HPO_4, 22

mM KH_2PO_4, 8.5 mM NaCl, 1 g/L (18.3 mM) $^{15}NH_4Cl$, 3 g/L glucose, 0.1 mM $CaCl_2$, 1 mM $MgSO_4$, 5 µM $ZnCl_2$, 1 x MEM Vitamin solution (Life Technologies), trace minerals, 50 µg/L kanamycin. The cells were grown to OD_{600} = 0.6 and induced with 1 mM IPTG for 3h at 37ºC. The cells were collected in MilliQ water and frozen in liquid nitrogen. After thawing, sonicating and sedimenting the insoluble material the soluble phase was diluted with MilliQ water to 80 mL/L equivalent of culture. The pH was adjusted to 4.5 with perchloric acid and the proteins were left to precipitate at 4ºC overnight. The insoluble phase was sedimented by centrifugation at 38000 x *g* for 20 min. The supernatant containing Ub and a small amount of contaminants was subjected to chromatography on a Source 15 S column (8 mL bed) in 10 mM Ammonium acetate (pH 4.5) and eluted with a gradient of NaCl in 10 mM Ammonium acetate. The equivalent of 1 L culture was separated in each run. Ub elutes at around 20 mS/cm conductivity. The protein was concentrated and washed in 10 mM HEPES pH 7.5 using Millipore filter Unit (3000 MW cut off). 5 mg of ^{15}N-Ub was phosphorylated with 0.2 mg GST-PINK1 (pediculus humanus) at 30ºC in the presence of 50 mM Tris pH 7.5, 50 mM NaCl, 0.5 mM ATP, 10 mM $MgCl_2$. The kinase was removed with Q-Sepharose FF, and the phospho Ub was desalted and purified to homogeneity on a MonoQ column. Phospho-Ub can bind to MonoQ in 5 mM Tris pH 7.5 (conductivity < 2 mS/cm) and eluted with a shallow gradient of NaCl at about 7 mS/cm. Unphosphorylated Ub does not bind to Q at this pH.

MALDI TOF DUB assays

Analysis of DUB assays was performed similar to described previously (Ritorto et al., 2014), 31 human DUBs were freshly diluted in the reaction buffer (40 mM Tris–HCl, pH 7.6, 5 mM DTT, 0.005% (w/v) BSA) at different concentrations **(Table S3)**. All diubiquitin isomers (phosphorylated and unmodified) were diluted to 0.2 µg/µl in the Ub dimer buffer (40 mM Tris–HCl, pH 7.6, 0.005% (w/v) BSA) and used as substrates at a fixed concentration (1.5 µM). Reaction buffer, DUBs and Ub dimers were aliquoted in a 384 well plate; the plate was sealed and kept in ice until use. A Mosquito nanoliter pipetting system (Labcyte, Sunnyvale, CA, USA) was programmed to aliquot 1.92 µL of reaction buffer and 1.2 µL of enzyme in duplicate from a new master plate. Enzymes were pre-incubated in the reaction buffer for 5 min and afterwards 0.48 µL of diubiquitin topoisomers were added to the reaction mixture with a 3 times mixing cycle to assure homogeneity within the reaction volume. The reaction was sealed and incubated for 60 min at room temperature. The reaction was stopped by adding 10% TFA to a final concentration of 2% (v/v). Pipetting time was calculated in order to avoid incubation time biases between the first and the last column of the plate. 1.050 µL of each reaction was transferred to a new plate and spiked with 0.15 µL of 16 µM ^{15}N-labelled unmodified or Ser65 phosphorylated Ub as internal standard and mixed 1:1 with freshly prepared 2,5-dihydroxyacetophenone (DHAP) matrix (7.6 mg of 2,5-DHAP in 375 mL ethanol and 125 mL of an aqueous 12 mg/mL diammonium hydrogen citrate). Sample and matrix were mixed 20 times and 200 nL of the mixture was spotted in duplicate onto MTP AnchorChip 1,536 TF targets (600 mm anchor, Bruker Daltonics).

Mass spectrometry data was acquired on an UltrafleXtreme MALDI-TOF mass spectrometer (Bruker Daltonics) with Compass 1.3 control and processing software. The sample carrier was taught before each analysis to optimize and centre laser shooting. Internal calibration was performed before each analysis using the ^{15}N-Ub peak $[M+H]^+$ average = 8,569.3). Samples were run in automatic mode (AutoXecute, Bruker Daltonics). Ionization was achieved by a 2-kHz smartbeam-II solid state laser

with a fixed initial laser power of 60% (laser attenuator offset 68%, range 30%) and detected by the FlashDetector at detector gain of x10. Reflector mode was used with optimized voltages for reflector-1 (26.45 kV) and reflector-2 (13.40 kV), ion sources (IonSource-1: 25.0 kV, IonSource-2: 22.87 kV) and pulsed ion extraction (320 ns). An amount of 3,500 shots were summed up in 'random walk' and with 'large' smartbeam laser focus. Spectra were automatically calibrated on the ^{15}N-Ub m/z and processed using smoothing (Savitzky–Golay algorithm) and baseline subtraction ('TopHat') for reproducible peak annotation on non-resolved isotope distributions: one cycle, 0.2 m/z for the width. For area calculation, the complete isotopic distribution was taken into account. For comparison between phosphoubiquitin isomers, equal ionization efficiencies in MALDI were assumed. An in-house made script was used to report ^{15}N/phospho-^{15}N and monoubiquitin/phospho-monoubiquitin areas; plotting of graphs, calculation of standard deviation and coefficient of variation (%) were processed in Microsoft Excel.

Targeted mass spectrometry using AQUA peptides

Sample preparation
10 µg of each purified Ub trimers as well as 10 µg of K63-linked Ub dimer phosphorylated at position 65 were run on SDS-PAGE gels and briefly stained using InstantBlue (Expedeon). The bands were excised and cut into small pieces. Gel pieces were sequentially washed in water, 50% (v/v) acetonitrile, 0.1 M ammonium bicarbonate and overnight in 50% (v/v) acetonitrile in 25 mM ammonium bicarbonate at 4˚C. Gel pieces were dehydrated by addition of acetonitrile and solvents evaporated using a SpeedVac Concentrator (Eppendorf). The gel pieces were then rehydrated in 25 mM triethylammonium bicarbonate buffer pH 8.5 (Sigma-Aldrich) containing 4 µg of trypsin (Pierce) per 10 µg of Ub and incubated overnight at 30˚C with intermittent shaking. An equivalent volume of acetonitrile was added to the solution and incubated at room temperature for an additional 15 min. The solution was transferred to a new tube, flash frozen, and sublimated using a SpeedVac Concentrator. Simultaneously the gel pieces were incubated with 50% (v/v) acetonitrile in 2.5% (v/v) formic acid. This solution was added to the lyophilized first extract, flash frozen and evaporated using a SpeedVac Concentrator.

Preparation of Ub-AQUA Peptide Mixtures
Concentrated stocks of isotopically labelled internal standard (heavy) peptides and light synthetic peptides (M1, K6, K11, K27, K29, K33, K48, K63) were purchased from Cell Signaling Technologies, whereas phosphorylated and GG modified peptides were purchased from CRB Cambridge Research biochemical **(Data S1)**. All stock solutions were stored at -80°C, working stock solutions of individual peptides were prepared at 25 pmol/µL in 2% (v/v) ACN, 0.1% (v/v) FA and used to prepare an experimental mixture consisting of 25 peptides at 1 pmol/µL in 2% (v/v) ACN, 0.1% (v/v) FA. Experimental mixtures were frozen at -80°C in duplicate use aliquots for direct addition to samples.

Absolute quantitation by Parallel Reaction Monitoring (PRM)
PRM quantitation was performed on an Orbitrap Fusion mass spectrometer (Thermo-Fisher Scientific) with an Easy-Spray source coupled to an Ultimate 3000 Rapid Separation LC system (Thermo Fischer Scientific). Samples were loaded via a 5 µL full loop injection directly onto an EASY-Spray column (15 cm x 75 µm ID, PepMap

C18, 3 µm particles, 100 Å pore size, Thermo-Fisher Scientific) and separated by reverse phase chromatography at a flow rate of 1.00 µL/min where solvent A was 98% (v/v) H_2O, 2% (v/v) ACN, (v/v) 0.1% FA and solvent B was 98% (v/v) ACN, 2% (v/v) H_2O, 0.1% (v/v) FA. Upon LC direct injection, peptides were resolved with an isocratic gradient of 0.1% of solvent B over 10 min, followed by a step from 0.1% to 25.5% of solvent B over 41 min, 5 min of high organic wash (90% solvent B) and 12 min re-equilibration at 0.1% of solvent B. The Orbitrap Fusion mass spectrometer was operated in targeted mode "tMS2" for the detection of light and synthetic heavy peptides. The included m/z values were selected by the quadrupole, with 4 m/z isolation window, a maximum injection time of 100 ms and a maximum AGC target of 5e4. HCD fragmentation was performed at 30% collision energy for all included peptides and MS/MS fragments were detected in the Orbitrap mass analyzer at a FMWH resolution of 30,000 (at m/z 200). Peak integration of MS/MS spectra and quantification of Ub peptides were performed on Skyline (version 3.5.0.9191) (http://proteome.gs.washington.edu/software/skyline) (MacLean et al., 2010). Confident peak integration corresponding to the extracted ion chromatogram of MS/MS fragments for a selected precursor was adjusted manually to avoid potential interferences and quantitation was performed by integrating areas of peaks corresponding to the endogenous and heavy *m/z* values.

5. Supplemental References

Keusekotten, K., Elliott, P.R., Glockner, L., Fiil, B.K., Damgaard, R.B., Kulathu, Y., Wauer, T., Hospenthal, M.K., Gyrd-Hansen, M., Krappmann, D., et al. (2013). OTULIN antagonizes LUBAC signaling by specifically hydrolyzing Met1-linked polyubiquitin. Cell *153*, 1312–1326.

MacLean, B., Tomazela, D.M., Shulman, N., Chambers, M., Finney, G.L., Frewen, B., Kern, R., Tabb, D.L., Liebler, D.C., and MacCoss, M.J. (2010). Skyline: an open source document editor for creating and analyzing targeted proteomics experiments. Bioinformatics *26*, 966–968.

Ritorto, M.S., Ewan, R., Perez-Oliva, A.B., Knebel, A., Buhrlage, S.J., Wightman, M., Kelly, S.M., Wood, N.T., Virdee, S., Gray, N.S., et al. (2014). Screening of DUB activity and specificity by MALDI-TOF mass spectrometry. Nat Comms *5*, 4763.

Sato, Y., Goto, E., Shibata, Y., Kubota, Y., Yamagata, A., Goto-Ito, S., Kubota, K., Inoue, J.-I., Takekawa, M., Tokunaga, F., et al. (2015). Structures of CYLD USP with Met1- or Lys63-linked diubiquitin reveal mechanisms for dual specificity. Nat Struct Mol Biol *22*, 222–229.
